# Effect of Brominated Epoxy Resin Content on Thermophysical and Mechanical Properties of Intumescent Fire-Protective Coatings

**DOI:** 10.3390/polym18040484

**Published:** 2026-02-14

**Authors:** Vladimir Kukushkin, Vyacheslav Subbotin, Nikolay Yashin, Victor Avdeev

**Affiliations:** Faculty of Chemistry, Lomonosov Moscow State University, Moscow 119991, Russia; vladimir.kukushkin@chemistry.msu.ru (V.K.); subbotin.v@ograx.ru (V.S.); avdeev@highp.chem.msu.ru (V.A.)

**Keywords:** intumescent fire-protective materials, epoxy binders, combustion, brominated epoxy resin, thermal degradation, fire-retardant efficacy

## Abstract

Intumescent fire-protective coatings based on epoxy binders are widely used to enhance the fire resistance of steel structures due to their high adhesion, mechanical strength, and durability. However, epoxy binders undergo exothermic thermo-oxidative degradation, which can adversely affect fire-protective performance. In this study, the effect of brominated epoxy resin content on the fire-retardant behavior of intumescent coatings was investigated using two systems: one initially supporting flame propagation and one inherently self-extinguishing. For the initially combustible coating, partial substitution of the epoxy diane resin with a brominated analogue at 12.5% resulted in complete self-extinguishing behavior according to UL-94, while higher substitution levels (≥50%) caused a 20–28% reduction in fire-protective efficacy as assessed by BS 476. For the initially non-combustible coating, a decrease in fire-protective performance of 15–20% was observed regardless of the substitution degree. Thermal analysis showed that coatings containing brominated resins exhibit an onset of thermal degradation approximately 80 °C lower than halogen-free analogues. FTIR and SEM analyses revealed that brominated resins alter the thermolysis mechanism, promoting the formation of oxygen-containing degradation products and a more heterogeneous, irregularly porous foamed char, thereby reducing its thermal insulation capacity. Overall, brominated epoxy resins exert a dual effect, improving self-extinguishing behavior while impairing fire-protective efficacy under prolonged thermal exposure. Brominated resin contents in the range of 10–50% represent a practical compromise, enabling self-extinguishing behavior while maintaining acceptable fire-protective performance.

## 1. Introduction

Fires represent a major technological risk, and therefore, requirements for structural and equipment components emphasize not only operational characteristics (e.g., mechanical strength, recyclability, environmental impact) but also fire resistance and safety [1]. Fire protection can be achieved through flame-retardant or self-extinguishing materials, as well as measures that reduce fire damage, including intumescent fire-protective (IFP) coatings. IFP coatings can substantially enhance the fire resistance of structural elements even at relatively small thicknesses by forming an expanded, low-thermal-conductivity char layer under thermal stress [2,3,4]. Typically, IFP materials are dispersed systems, in which bloating agents, carbonizing agents, and fire retardants are distributed in a polymeric binder that cures on the substrate via physical and chemical transformations [5].

Among commonly used binders, epoxy resins are popular due to their ease of processing, durability under atmospheric exposure, and favorable mechanical properties such as hardness, adhesion, and impact resistance [5]. However, these systems can exhibit flammability, smoke generation, and toxic decomposition products [6]. Strategies to reduce flammability include the incorporation of fire-retardant additives or the chemical modification of the polymer matrix with fire-retardant fragments, which can render the coating self-extinguishing upon removal of the flame source [7,8].

The flammability of polymer matrices is commonly reduced by incorporating flame retardants such as phosphates [9], borates [10], hydrates, hydroxides, and metal oxides [11,12]. The type of fire retardant and its proportion relative to the binder and to other additives can shift polymer decomposition toward char formation, while decreasing the release of volatile combustible products. This affects not only the thermal behavior during oxidative degradation but also the composition, thermal and mechanical stability, and thermal conductivity of the char layer formed from IFP materials [13,14]. However, the amount of fire-retardant additives is limited, as excessive loading can deteriorate the thermomechanical properties and durability of the coating [15].

An additional key factor governing the effectiveness of intumescent systems is the synchronization of physicochemical transformations in the intumescent system. If the decomposition of the epoxy matrix (which serves as the char-forming agent) is not coordinated with action of the flame-retardant additives, the resulting foam char may exhibit cracks, cavities, or insufficient expansion, thereby reducing its thermal insulation efficiency [16]. In this context, the ratio of some fire retardants such as ammonium polyphosphate, which functions as an acid donor and gas-forming agent, to boric acid or zinc borate, which promote carbonization and char cohesion, is critical for maintaining char integrity and effective fire protection [17,18].

The thermomechanical stability of a polymer is closely related to its structural characteristics at both the monomer level (type and arrangement of functional groups) and at the macromolecular level (degree of polymerization and crosslinking density). These features determine the temperature range in which the formation of the foam char layer proceeds most effectively [15,19,20]. Consequently, modification of the chemical structure of the binder itself represents a promising strategy for tuning flammability, physicomechanical and thermophysical properties, and the composition of degradation products beyond what can be achieved solely through additive incorporation [21,22]. Incorporating flame-retardant fragments directly into the polymer chain also minimizes undesirable effects, such as migration or premature decomposition of additive flame retardants, which is particularly important for IFP coatings and contributes to improved service life [23].

Such structural modifications can be realized by increasing network rigidity through aromatic fragments (altering the C:H ratio) [24] or by introducing phosphorus [25], boron [26], and halogen-containing moieties [16]. These fragments inhibit radical reactions during combustion and thermal degradation, thereby directing polymer decomposition toward char formation. A widely used approach is the incorporation of halogen atoms into the polymer backbone. During thermal decomposition, halogenated polymers release radicals (e.g., Cl•, Br•) that scavenge high-energy H• and HO• radicals in the gas phase, suppressing chain-branching reactions and inhibiting flame propagation [27].

The effectiveness of halogens follows the trend F < Cl < Br < I [27]. In practice, iodine-containing compounds are generally too unstable, whereas fluorinated polymers are often too thermally stable to generate sufficient reactive F• radicals under fire conditions. Brominated flame retardants are therefore regarded as particularly effective, offering favorable decomposition behavior and strong flame inhibition at relevant temperatures. This has led to their widespread use as alternatives to many chlorinated analogues in commercial applications [28,29,30]. Regulatory considerations further influence this choice: chlorine-containing formulations face stricter restrictions and phase-out due to concerns over the formation of toxic dioxins and furans during combustion or waste incineration, whereas brominated compounds are regulated under substantially simpler requirements [9,31,32]. For instance, brominated compounds are widely used in high-performance applications, including composite materials in commercial aircraft [33].

**Figure 1:**
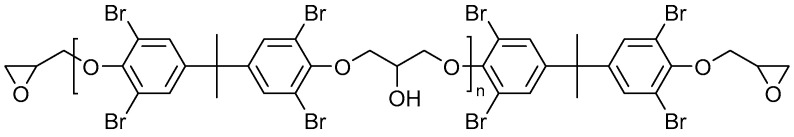
DGETBBA epoxy resin.

A prominent flame-retardant polymer is the brominated diane epoxy resin derived from tetrabromobisphenol A (TBBA). corresponding to the brominated diglycidyl ether (DGETBBA, Figure 1). TBBA-based resins are extensively employed as a reactive or additive flame retardant in epoxy-based composites to reduce flammability and achieve high UL 94 ratings [15,34,35,36,37]. However, to date, TBBA-containing intumescent formulations specifically designed to enhance the fire resistance of structural elements have not been reported, and systematic experimental data on the effect of TBBA on the fire-protective performance of intumescent coatings remains unavailable.

Varying the bromine content of a halogenated polymer binder is a key strategy for reducing the flammability of epoxy-based intumescent coatings. At the same time, bromine content strongly affects the degradation mechanism of the polymer matrix, the structure of the resulting char [16], and, consequently, the coating’s fire-retardant performance and mechanical properties [35]. The bromine content in fire-protective formulations can be adjusted either by modifying the degree of bromination of the epoxy resin or hardener [38], or by blending a brominated epoxy resin with a halogen-free or partially halogenated resin [35,39]. Accordingly, the present study systematically investigates how the content of a bromine-containing epoxy binder influences fire-retardant performance, flammability, adhesion to the substrate, thermal expansion factor, and thermal degradation behavior of epoxy-based intumescent fire-protective coatings.

## 2. Materials and Methods

### 2.1. Materials

The present study focused on two intumescent fire-protective (IFP) formulations using epoxy resins based on bisphenol A as the binder: NPEL-128 and NPEB-400 (Nan Ya Plastics Corporation, Kaohsiung City, Taiwan). NPEL-128 is a liquid polymer derived from the diglycidyl ether of bisphenol A (DGEBA), whereas NPEB-400 is a solid, tetrabrominated derivative of bisphenol A (TBBA). The properties of these resins are summarized in Table 1 and Table 2.

### 2.2. IFR Formulations

**Figure 2:**
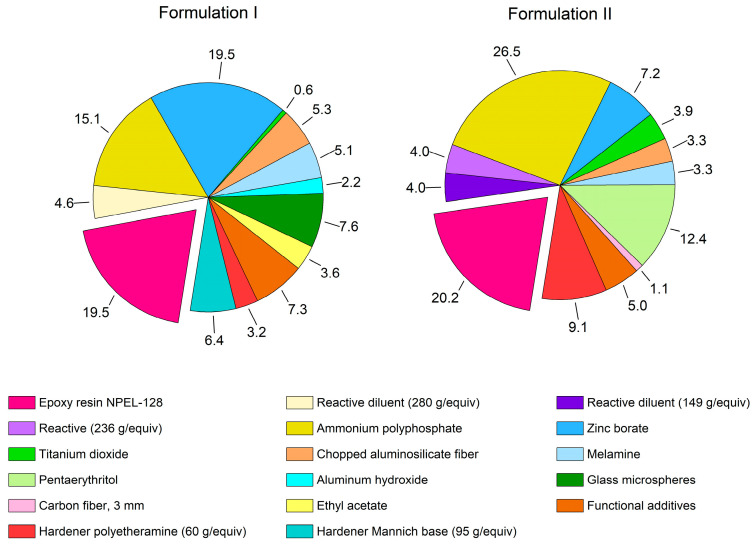
Composition of formulations **I** and **II**.

Two initial two-component formulations, **I** and **II** (Table 3 and Table 4, Figure 2), based on the bromine-free NPEL-128 resin, were employed in this study. In subsequent derivative formulations, this resin was replaced with its TBBA-containing analogue NPEB-400 to investigate the effect of bromine incorporation.

**Figure 3:**
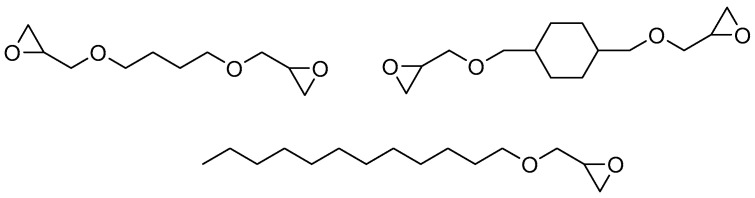
Chemical structures of the epoxy reactive diluents used in this study.

A key feature of using NPEB-400, a bromine-containing solid resin, is that it must be converted to a liquid state for processing into a fire-retardant coating. To achieve this, reactive diluents (Figure 3) were introduced into the system. These diluents are relatively low-viscosity compounds with epoxy groups, which are then incorporated into the polymer matrix under the action of the hardener [40,41]. Ethyl acetate and acetone of technical grade were used as solvents.

The intumescent system of the compositions used ammonium polyphosphate KYLIN APP201 (Shifang Changfeng Chemical Co., Deyang City, China, crystalline phase II, avg. degree of polymerization ≥ 50), zinc borate HT-207 (Jinan Taixing Fine Chemicals Co., Jinan, China, weight fraction B_2_O_3_ 45–80%, ZnO 36–39%, loss on ignition at 450 °C was 13–15%), melamine (Sichuan Golden-Elephant Sincerity Chemical Co., Meishan, China, purity ≥ 99.8%), titanium dioxide TiOx-280 (CRIMEA TITAN, weight fraction of TiO_2_ ≥ 93.5%, rutile form ≥ 98%), chopped aluminosilicate fiber FiberFrax B100 (Unifrax, Tonawanda, NY, USA, weight fraction of Al_2_O_3_ 42–50%, SiO_2_ 50–58%, avg. fiber diameter 2.1 μm), carbon fiber (Haining ANJIE Composite Materials Co., Jiaxing, China, length 3 mm, diameter 7–10 nm), hollow glass microspheres ForeSphere 1500 (Fortisphere, Chantilly, VA, USA, bulk density 0.21–0.27 g/cm^3^, moisture content ≤ 0.5%), pentaerythritol (Metafrax Chemicals, Perm, Russian, purity ≥ 95%, moisture and volatile content ≤ 0.2%). Chemically pure ethyl acetate (Bina Group, Moscow, Russia, purity ≥ 99%, moisture ≤ 0.1% and non-volatile content ≤ 0.001%) and acetone of technical grade (Ruskhimset, Moscow, Russia, purity ≥ 99.75% and moisture ≤ 0.2%) were used as solvents.

The SEM images reveal the morphology, particle size, and distribution of the main powder fire-retardants used in the preparation of the IFP formulations under study, namely ammonium polyphosphate, aluminum hydroxide, melamine, glass microspheres (Figure 4 and Figures S1–S16), titanium dioxide (Figures S17–S21), zinc borate (Figures S22–S26), pentaerythritol (Figures S28–S31), chopped aluminosilicate fiber (Figures S32 and S33), and carbon fiber (Figures S34 and S35). 

### 2.3. Methods for Preparing the IFP Formulations Investigated

#### 2.3.1. Preparation of Formulations **IA** and **IIA** with NPEL-128 Resin

Formulations **IA** and **IIA** were prepared by initially mixing the binder with the functional additives using a laboratory dissolver (Dispermill Vango 100, ATP Engineering B.V., Almere, The Netherlands) at 2000 rpm for 30 min. The powder components were then gradually added and mixed under the same conditions for an additional 30 min. Subsequently, the respective solvents and fillers were incorporated: ethyl acetate and glass microspheres for formulation **IA**, and carbon fiber for formulation **IIA**. Finally, each mixture was homogenized for 5 min at 2000 rpm.

#### 2.3.2. Preparation of Formulations **IA** and **IIA** with NPEB-400 Resin

Formulations **IA** and **IIA** containing the bromine-containing NPEB-400 resin were prepared using a similar procedure to that described for NPEL-128, with the following modifications. The NPEB-400 resin was first pre-crushed in a mortar and then added portionwise after the powder components into the dispersion, which was stirred and heated along with the respective solvent: ethyl acetate for formulation **IA** (Table 1) and acetone (80% NPEB-400 solution) for formulation **IIA**. Stirring continued until the mixture was homogeneous. Subsequently, glass microspheres were added to formulation IA and carbon fiber to formulation **IIA**, and stirring was maintained for an additional 5 min at 2000 rpm. Finally, the remaining solvent in both formulations was evaporated in a drying oven at 80 °C until a constant mass of dry residue was obtained.

#### 2.3.3. Preparation of Formulations **IA** and **IIA** with NPEL-128 and NPEB-400 Resin Mixture

To vary the bromine content, a series of formulations **IA** and **IIA** were prepared by mixing the bromine-free NPEL-128 formulation (Section 2.3.1) with the bromine-containing NPEB-400 formulation (Section 2.3.2). The mixing ratios ranged from neat NPEL-128 (100/0) to neat NPEB-400 (0/100) formulations—100/0, 75/25, 50/50, 25/75, and 0/100—while maintaining a constant total epoxy equivalent weight, as detailed in Table 5.

#### 2.3.4. Preparation of Formulations **IB** and **IIB**

The ingredients of formulation **IB** (Table 4) were mixed into formulation **IA** using a laboratory dissolver immediately before sample preparation. For formulation **IIB**, all ingredients listed in Table 4 were mixed in a laboratory dissolver at 2000 rpm for 30 min.

#### 2.3.5. Preparation of the Compounds for Formulations **I** and **II**

**Figure 5:**
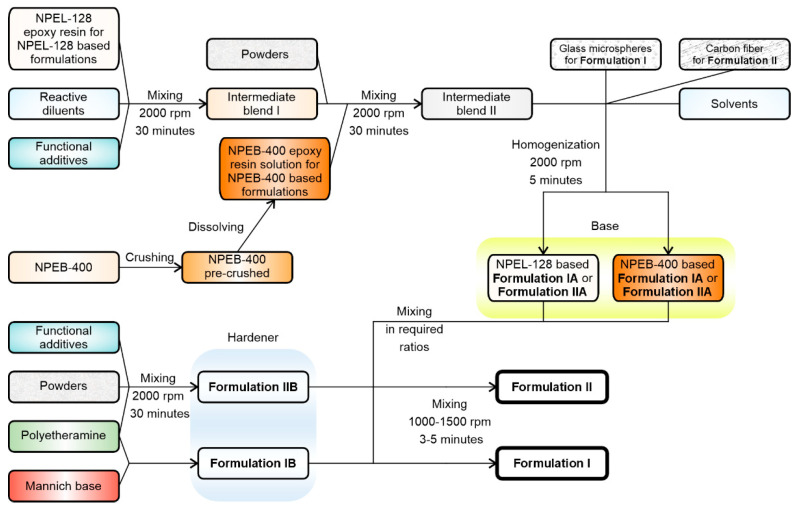
Scheme for the preparation of compounds for formulations **I** and **II**.

For each formulation, the respective components (**IA and IB** or **IIA and IIB**) were mixed in an equivalent mass ratio for 3–5 min at 1000–1500 rpm to ensure homogeneity and maintain the temperature below 60 °C to avoid overheating (Figure 5).

### 2.4. Characterization Methods of IFP Coatings

#### 2.4.1. Flammability Testing and Self-Extinguishment Evaluation

Self-extinguishing properties were evaluated using the UL-94 HB (horizontal burning) and UL-94 V (vertical burning, 20 mm flame application) test methods. To prepare the specimens, the cured compounds were poured into molds and allowed to cure for at least 48 h. The molds were then opened, and the cast specimens were conditioned for an additional 24 h at 23 ± 2 °C and 50 ± 5% relative humidity. Rectangular test specimens with dimensions of 125 ± 5 mm × 13 ± 0.5 mm × 3 ± 0.2 mm were cut from the castings and further conditioned for at least 48 h under the same conditions.

During the UL-94 HB test, the total self-extinguishing time (*T_H_*) was determined as the sum of the burning times of three specimens after the flame passed the 25 mm mark from the point of application. During the UL-94 V test, the total self-extinguishing times after the first (*T_V_*_1_) and second (*T_V_*_2_) flame applications, as well as the afterglow time (*T_V_*_3_), were recorded as the sums for five specimens for each formulation.

#### 2.4.2. Methods for Assessing Fire-Retardant Efficacy

Fire-retardant efficacy was assessed in accordance with the BS 476 standard methodology. The compounds were applied as two-layer coatings using a film applicator onto primed and degreased steel plates measuring 250 × 250 × 5 mm. Each plate was equipped with a thermocouple hole drilled on the unexposed side, which was thermally insulated. After application, the coated specimens were conditioned for at least 168 h at 23 ± 2 °C and 50 ± 5% relative humidity. The dry coating thickness was then measured using a magnetic thickness gauge.

The prepared specimens were tested in a specialized furnace for small-scale steel structures under standard cellulose fire conditions, in accordance with BS 476 (Part 20). The standard temperature–time curve is described by the following equation:

$$T=345\log_{10}(8t+1)+20$$

where *T* is the average furnace temperature (°C) and *t* is the exposure time (min), not exceeding 360 min.

Fire-retardant efficacy was quantified as the time required for the temperature on the unexposed (back) surface of the coated steel plate to reach 500 °C, measured from the onset of heating.

#### 2.4.3. Pull-Off Adhesion Testing

Adhesion to the substrate was evaluated in accordance with ISO 4624:2023 [42]. The formulations were applied onto primed and degreased steel plates measuring 150 × 70 × 4 mm using a film applicator. The coated samples were conditioned for at least 168 h at 23 ± 2 °C and 50 ± 5% relative humidity. Three cleaned and degreased dollies were then bonded to the coating surface using a structural adhesive and conditioned for an additional 24 h under the same conditions. After curing, excess adhesive was removed, and the coating was cut down to the substrate around the perimeter of each dolly. The pull-off adhesion strength was subsequently measured using a universal testing machine (Tinius Olsen 50 ST, Tinius Olsen, Inc., Horsham, PA, USA).

#### 2.4.4. Measurement of the Expansion Factor

Thermal expansion under rapid heating (thermal shock) was evaluated as follows. Square specimens with dimensions of 40 ± 5 mm × 40 ± 5 mm were cut from the cured coatings. The initial dry film thickness, *h*_0_, was measured using a magnetic thickness gauge. The specimens were then placed on a non-combustible, heat-resistant substrate and exposed in a muffle furnace at 600 °C for 5 min. After cooling to ambient temperature, the thickness of the expanded char layer, *h*_1_, was measured using a caliper. The expansion factor was calculated as the ratio *h*_1_*/h*_0_.

#### 2.4.5. Thermal Analysis

The thermal behavior of the prepared IFP materials was characterized for both the final compounds and the binder samples of formulations **I** and **II** (Table 3 and Table 4), based on NPEL-128 and NPEB-400 resins in different ratios (Table 5). The primary techniques employed were thermogravimetric analysis [43] and differential scanning calorimetry [44].

Thermogravimetric Analysis (TGA): TGA of the polymer matrices and corresponding compounds was performed using a thermal analyzer (STA 449, NETZSCH-Gerätebau GmbH, Selb, Germany). Measurements were carried out in corundum crucibles under an air atmosphere with a flow rate of 100 mL/min. Samples were heated from 40 to 900 °C at a rate of 20 °C/min, followed by a 1 h isothermal hold at 900 °C.

Differential Scanning Calorimetry (DSC): DSC analysis of the polymer matrices was conducted in accordance with ISO 11357-2:2020 [45], using a heat-flux differential scanning calorimeter (DSC Q20, TA Instruments, New Castle, DE, USA). Samples were sealed in aluminum crucibles and measured under a high-purity nitrogen atmosphere (≥99.999%) at 100 mL/min. The temperature program ranged from –50 to 180 °C to determine the glass transition temperature (*T_g_*) accurately.

SEM observations were performed using a Vega 3 microscope (Tescan, Brno–Kohoutovice, Czech Republic) equipped with secondary and backscattered electron detectors and operated at an accelerating voltage of 20 kV.

FTIR spectra were recorded using a Tensor 27 spectrometer (Bruker Optik GmbH, Baden-Württemberg, Germany).

## 3. Results and Discussion

The primary property of IFP materials designed for the protection of steel structures is their fire-retardant efficacy. This corresponds to the duration during which a protected structure can maintain its load-bearing capacity, allowing for the evacuation of occupants and fire-fighting operations. Increasing this parameter is often the primary objective in the development of new IFP formulations for metal protection.

The mechanism of IFP materials relies on the coordinated decomposition of their components under thermal shock, leading to the formation of a foamed char layer with low thermal conductivity and high thermal stability under exposure to high temperatures and hot gas flows for 30–150 min. IFPs are polymer-based composite coatings, and increasing the polymer binder content is generally desirable to enhance coating durability and mechanical properties, particularly adhesion to the metal substrate. However, due to the inherent flammability of polymers, such an increase can also lead to higher combustibility, which may impair the formation of a coherent foamed char layer under thermo-oxidative degradation.

In this context, it is useful to leverage experience with brominated epoxy resins to obtain non-flammable polymeric compositions. Incorporating brominated resins is intended not only to suppress combustion of the IFP coating but also to slow the exothermic decomposition processes, thereby delaying heat transfer to the protected metal surface. However, the introduction of bromine into the resin can significantly alter the polymer matrix decomposition mechanism, making the dual objectives of reducing flammability and enhancing fire protection efficiency for steel structures potentially conflicting.

To describe strategies for achieving self-extinguishing behavior in epoxy-based IFP materials, it is essential to consider the thermal-oxidative degradation of their polymer matrix under fire conditions. Thermal-oxidative degradation of polymers, including epoxies, involves coupled processes in the condensed and gas phases. Upon exposure to external heat, the polymer initially decomposes in the condensed phase, releasing flammable volatiles such as lower alkanes, alkenes, acetone, and aldehydes. These volatiles, in the presence of oxygen, are subsequently oxidized to CO and CO_2_, accompanied by a strong exothermic effect. This gas-phase oxidation releases heat, which accelerates further thermal decomposition of the polymer, thereby promoting the propagation of combustion. Overall, this process can be represented as the combustion of hydrocarbon fuel (1), essentially a branched chain of radical reactions, the main steps of which are given in reactions (2)–(7) [46,47].

RH + O_2_ → CO + CO_2_ + H_2_O + Q (1)

RH → R• + H•(2)

RH + O_2_ → RHO• + OH• (3)

RHO• + O_2_ → CO + H• (4)

H• + O_2_ → HO• + 0.5O_2_ (5)

RH + OH• → R• + H_2_O (6)

OH• + CO → CO_2_ + H• (7)

Even trace amounts of highly reactive radicals such as H• and HO•, generated during polymer decomposition, play a critical role in ignition. Replacing these radicals in the gas phase with less reactive species promotes recombination reactions, reducing the concentration of high-energy radicals. This suppresses combustion and can lead to self-extinguishment. Simultaneously, in the condensed phase, flame retardancy is enhanced through the formation of protective barriers that inhibit mass and heat transfer. Additionally, the endothermic generation of non-flammable gases during pyrolysis, such as CO_2_, N_2_, and H_2_O_(g)_, contributes to dilution of flammable volatiles and lowers the temperature in the combustion zone [47].

Since epoxy polymer matrices serve as a carbon source for the insulating char layer through pyrolysis-driven processes such as cyclization, condensation, and recombination [48], any reduction in their flammability can significantly compromise the fire-retardant efficacy. In general, the thermal decomposition of brominated polymers proceeds via a series of radical reactions (8)–(15) [28], which compete with the primary combustion steps (2)–(7).

RBr → R• + Br•(8)

RBr + H• → R• + HBr(9)

R’H + Br• → R’• + HBr(10)

Br• + Br• → Br_2_ (11)

Br_2_ + H• → HBr + Br• (12)

O• + HBr → HO• + Br•(13)

HO• + HBr → H_2_O + Br• (14)

H• + HBr → H_2_ + Br• (15)

Brominated derivatives can generate halogen radicals (Br•) during thermolysis. These radicals readily abstract hydrogen from available molecules, forming HBr (reaction 10) [49]. Simultaneously, if molecular bromine (Br_2_) forms (reaction 11), it reacts with and consumes highly reactive H• radicals produced from non-halogenated polymer fragments (reaction 12). In the gas phase, HBr participates in key reactions (13)–(15), effectively scavenging H• and HO• radicals that sustain the combustion chain reaction. The collective effect of these radical-mediated steps (8)–(15) is referred to as chemical inhibition of combustion. Additionally, HBr acts as a physical inhibitor by diluting the flammable gas mixture and reducing the flame temperature.

Our previous study [50] showed that coatings derived from formulation **I** (halogen-free NPEL-128 resin) support flame propagation under direct fire exposure, whereas coatings from formulation **II** (also based on NPEL-128) are non-flame-spreading. Evidence suggests that the ability of a coating to resist flame spread is strongly influenced by the balance between its flame-retardant components in particular, ammonium polyphosphate/zinc borate (Table 3 and Table 4). The fire-retardant efficacy of formulation **II** exceeds that of formulation **I** by only 3% (Table 6), indicating that both formulations provide nearly equivalent overall fire protection. Consequently, it is of particular interest to investigate the correlations between the self-extinguishing behavior and flammability of IFP coatings and their fire-retardant performance as a function of brominated epoxy resin content.

The purpose of incorporating formulation **II** in this study was to evaluate the effect of brominated resin content on a coating system that is inherently flame-retardant. This approach enables an isolated assessment of the influence—whether beneficial or detrimental—of bromine-mediated flame-retardant mechanisms, specifically the HBr-driven processes (9)–(15), on fire-protective performance. These gas-phase inhibition mechanisms are closely coupled to the thermal degradation of the halogenated polymer and, in turn, induce structural modifications in the polymer matrix of the intumescent coating under thermal stress. Such modifications ultimately govern the thermal insulation efficiency of the resulting foamed char layer.

### 3.1. Formulation **I**

The key properties of the IFP coating derived from formulation **I**—including its self-extinguishing ability, fire-retardant efficacy, degree of thermal expansion, and adhesion—are summarized in Table 7.

**Figure 6:**
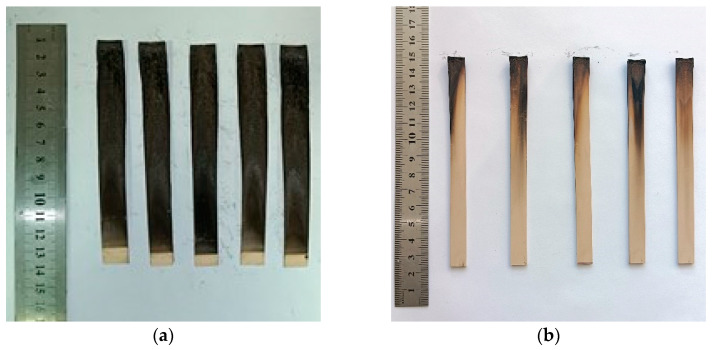
Burned specimens of formulation **I** after UL-94 VB testing, illustrating the self-extinguishing behavior induced by the brominated NPEB-400 resin. NPEL-128/NPEB-400 ratios: (**a**) 100/0 and (**b**) 87.5/12.5.

The UL-94 HB test demonstrated that all samples of formulation **I** were self-extinguishing immediately after removal from the flame, with the flame failing to reach the 25 mm mark, from which the spread rate is calculated. The UL-94 VB test indicated that introducing 2.4–2.5% bromine into the composition was sufficient to achieve a V-0 rating, as all bromine-containing samples self-extinguished within a short period after flame removal (Figure 6).

**Figure 7:**
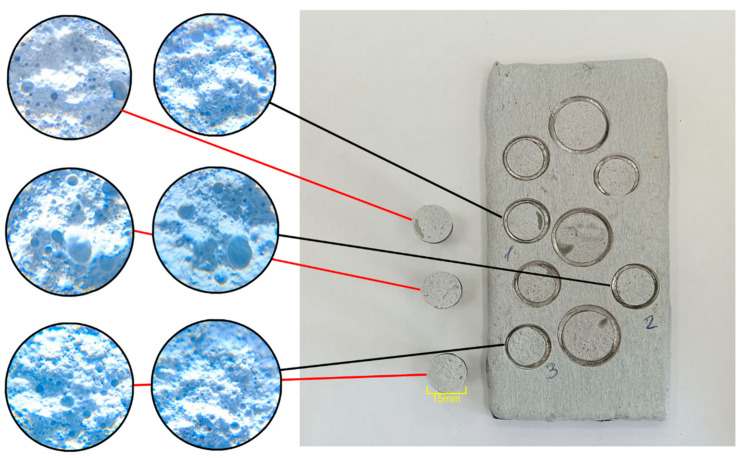
Macroscopic images of adhesion test samples after pull-off, along with microscopic views of their failure surface.

**Figure 8:**
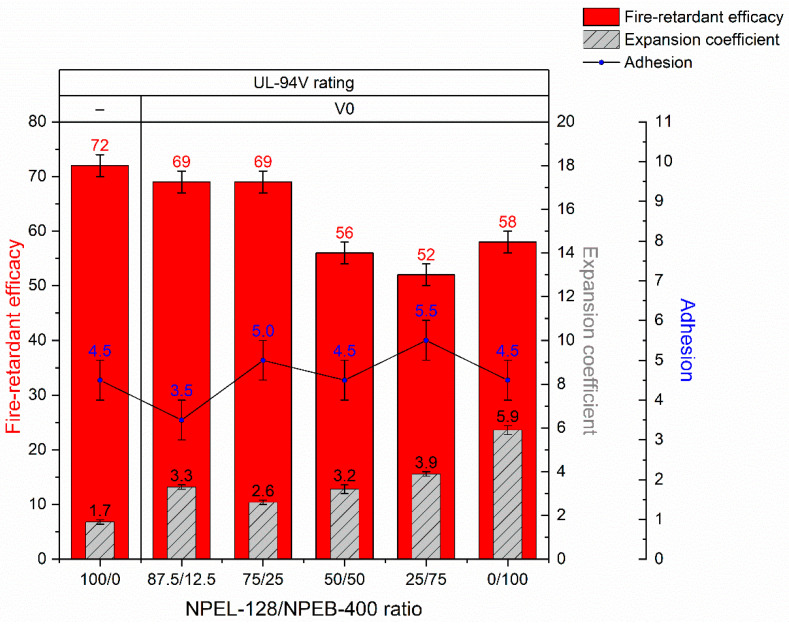
Test results summary graph for IFP coatings obtained from formulation **I**.

Fire-test results indicate that replacing up to 25% of NPEL-128 with the TBBA-containing resin NPEB-400 in formulation **I** does not produce a noticeable change in fire-retardant efficacy, whereas further substitution leads to a reduction in this property. The addition of the halogenated resin does not affect coating adhesion, as all samples exhibited cohesive failure (Figure 7 and Figures S36–S40), indicating adequate bond strength to the primed substrate. An increase in the expansion factor was observed, with the most pronounced effect seen in the 100/0, 87.5/12.5, and 0/100 formulations, while differences at intermediate brominated-resin concentrations were less significant (Figure 8).

**Figure 9:**
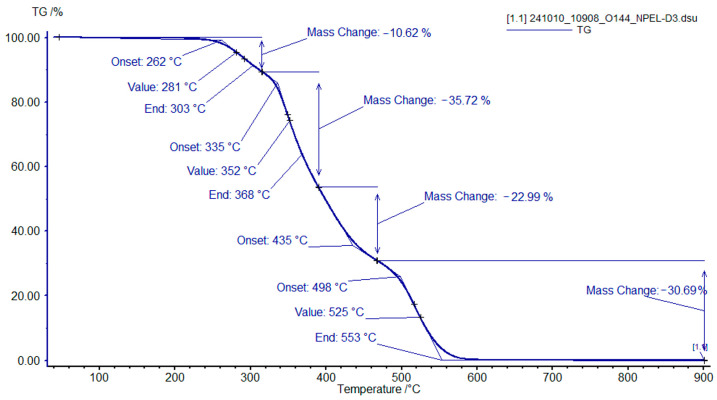
TGA curves of the binder sample with an NPEL-128/NPEB-400 ratio of 100/0 from formulation **I**, recorded in air.

TGA of the cured polymer matrices of formulation **I** revealed three decomposition stages. For the sample containing 100% NPEL-128 resin (Figure 9), the first stage, associated with dehydration and polymer network rearrangement, occurs between 250 and 350 °C. The second stage (350–450 °C) exhibits the largest weight loss, corresponding to cleavage and oxidation of aliphatic fragments. The third stage (450–580 °C) involves the complete oxidation of residual condensed structures.

**Figure 10:**
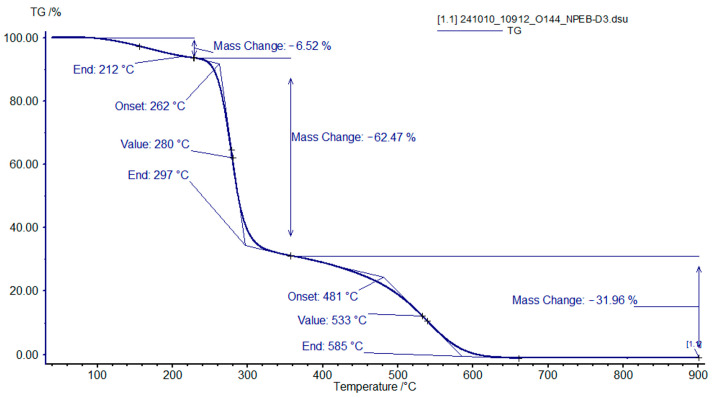
TGA curves of the binder sample with an NPEL-128/NPEB-400 ratio of 0/100 from formulation **I**, recorded in air.

In the case of the brominated analogue NPEB-400 (Figure 10), the first (100–250 °C) and second (250–320 °C) stages initiate and conclude at lower temperatures, while the third stage (320–610 °C) is extended under the same heating rate.

Both polymer binders undergo complete oxidation with no ash residue within the range of 580–610 °C in air.

**Figure 11:**
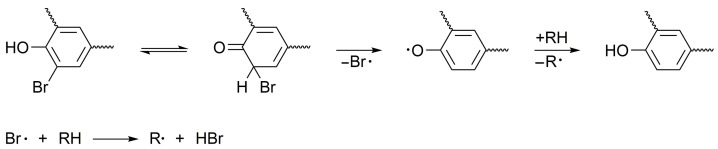
Formation of HBr during thermal destruction of bromine-containing epoxy resin.

The key distinction in the behavior of the formulations based on NPEL-128 and NPEB-400 resins lies in the condensed phase. The amine-crosslinked TBBA resin decomposes at lower temperatures than its non-brominated analog. This is attributed to a catalytic mechanism initiated by the nucleophilic attack of the amino group on the aromatic ring, leading to bromine substitution and accelerated thermal degradation [48]. Subsequent decomposition involves dehydration and dehydrohalogenation, generating hydrogen bromide (HBr) as the primary inhibitory species (Figure 11) [49].

**Figure 12:**
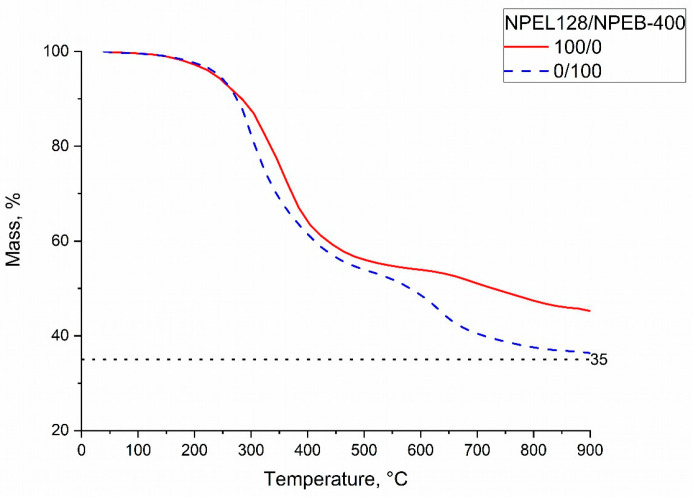
TGA curves of formulation **I** samples with NPEL-128/NPEB-400 resin ratios of 100/0 (red) and 0/100 (blue).

TGA of compounds from formulation **I** with 100/0 (NPEL-128) and 0/100 (NPEB-400) resin ratios (Figure 12) showed that the thermal decomposition of the corresponding IFP materials also occurs in three stages. In both cases, the first stage concludes at approximately 250 °C, followed by the second stage (250–450 °C), during which the largest mass losses are observed. The third stage for the 0/100 sample begins considerably earlier (550 °C) than for the 100/0 sample (650 °C). During this stage, up to 900 °C, the 0/100 sample exhibits lower thermal stability, with a final residue approximately 10% lower than that of the 100/0 sample.

**Figure 13:**
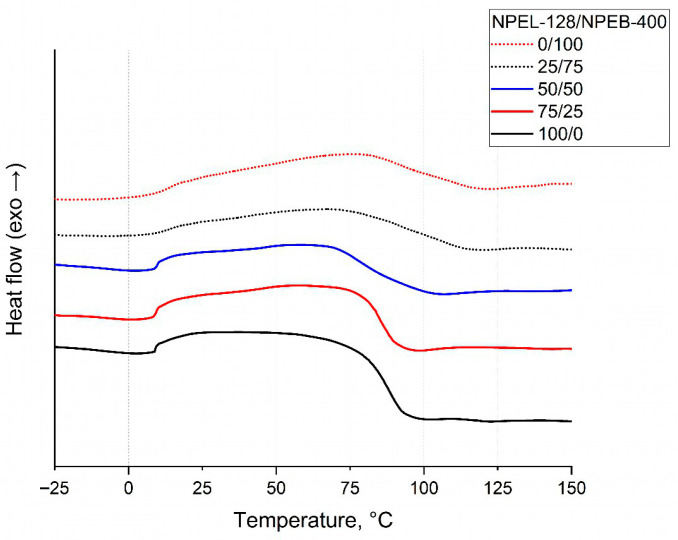
DSC analysis of binder samples from formulation **I** with varying NPEL-128/NPEB-400 ratios.

DSC analysis of the binders revealed an endothermic effect in the range of 75–115 °C. Furthermore, both the temperature range of this effect and its peak temperature were observed to increase with higher bromine content (Figure 13 and Table 8).

### 3.2. Formulation **II**

Table 9 presents the results for the self-extinguishing ability, fire-retardant efficacy, thermal expansion, and adhesion of the IFP coating based on formulation **II**.

As noted above, the study of formulation **II**, which is initially non-flame-spreading without a halogen-containing binder, was of particular interest for evaluating how the introduction of halogen affects the measured properties independently of the material’s inherent flammability.

Substituting 25% of the initial NPEL-128 epoxy polymer in formulation **II** with the TBBA-containing NPEB-400 analogue was expected to preserve the coating’s self-extinguishing behavior, but instead resulted in a noticeable decrease in the fire-protective performance of the IFP coating (from 74 to 59 min). Further substitution had no significant effect on flame-retardant efficacy.

**Figure 14:**
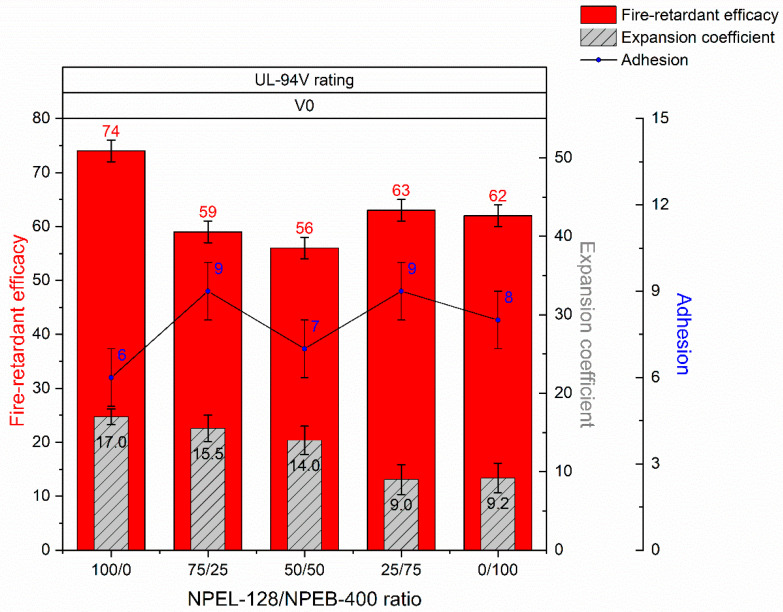
Comparative summary of fire-retardant, mechanical, and thermal performance test results for IFP coatings based on formulation **I**.

Adhesion measurements showed that all samples underwent cohesive failure, while increasing the content of the brominated binder led to a slight increase in tensile strength. The formulation also exhibited a general decrease in expansion coefficient under thermal exposure, particularly when more than 50% of the halogen-containing analogue was incorporated in an equivalent ratio (Figure 14).

**Figure 15:**
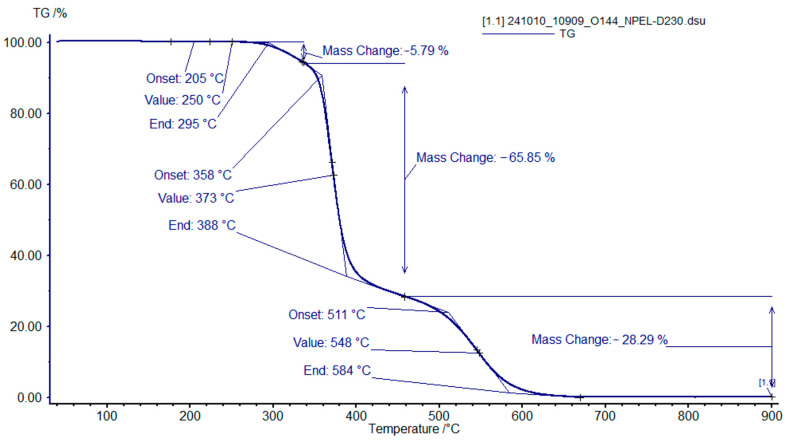
TGA curves of the binder sample with an NPEL-128/NPEB-400 ratio of 100/0 from formulation **II**, recorded in air.

**Figure 16:**
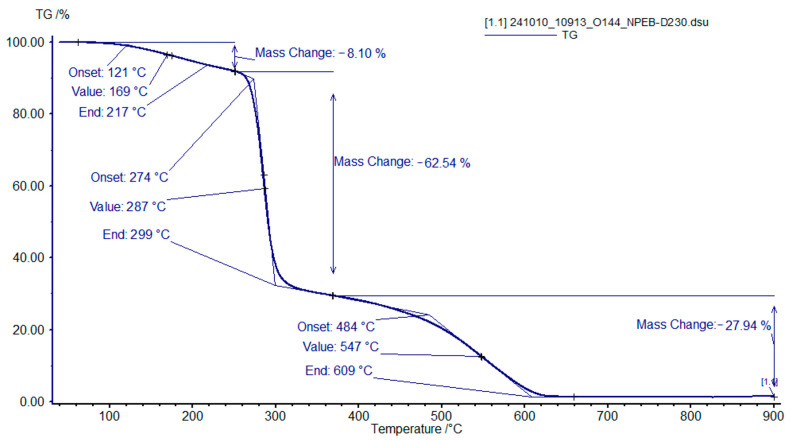
TGA curves of the binder sample with an NPEL-128/NPEB-400 ratio of 0/100 from formulation **II**, recorded in air.

TGA showed three distinct stages of the cured polymer binders of formulation **II** decomposition. A similar pattern of thermo-oxidative degradation is observed with the binders of formulation **I**. The sample with the 100% NPEL-128 resin (Figure 15) is more heat-resistant, with a noticeable weight loss observed in the range of 300–360 °C. The greatest weight loss also occurs in the second stage (360–400 °C), with the third stage completing at a temperature of 610–620 °C. For the sample with NPEB-400 (Figure 16), similarly to that for formulation **I**, the first (110–280 °C) and second (280–320 °C) stages begin and end earlier, with the third stage occurring in a wider temperature range (320–610 °C).

**Figure 17:**
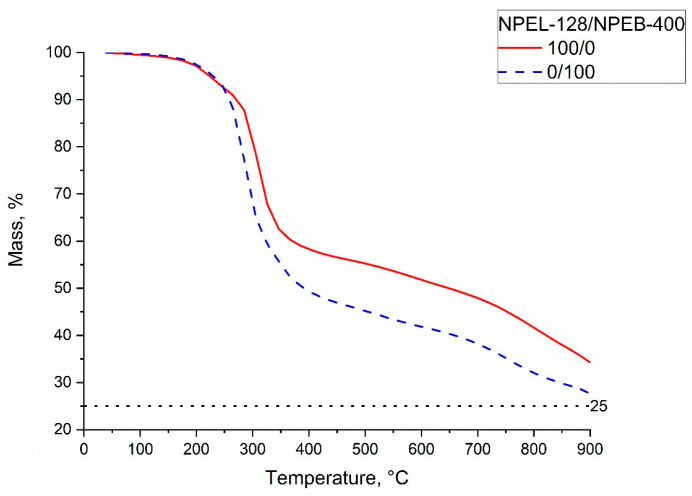
TGA curves of formulation **II** samples with NPEL-128/NPEB-400 resin ratios of 100/0 (red) and 0/100 (blue).

TGA of formulation **II** compounds with 100/0 and 0/100 resin ratios (Figure 17) showed that the thermal decomposition of the corresponding IFP materials occurs in three stages. In both cases, the first stage concludes at approximately 200 °C, followed by the second stage, which ends at 350 °C for the 100/0 sample and 400 °C for the 0/100 sample. At the end of the second stage, the residue of the sample containing the TBBA analogue is approximately 10% lower, a difference that persists throughout the third stage of decomposition up to 900 °C.

**Figure 18:**
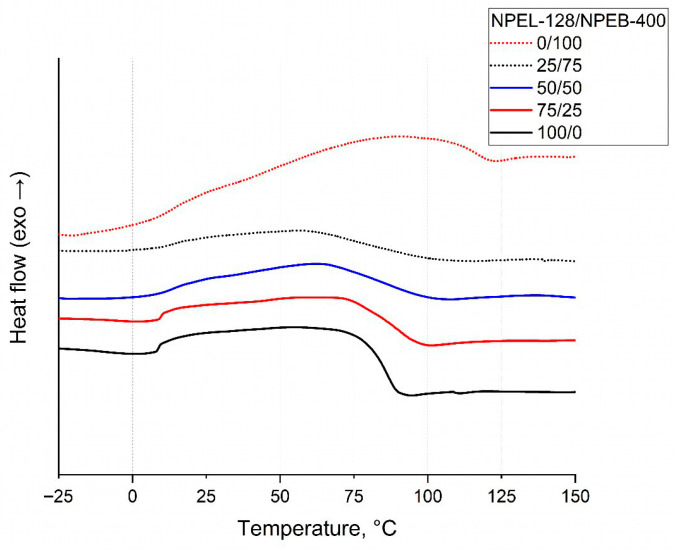
DSC analysis of binder samples from formulation **II** with varying NPEL-128/NPEB-400 ratios.

DSC analysis of the formulation **II** binders showed an endothermic transition between 60 and 125 °C. The temperature range of this effect increased and shifted toward higher temperatures (Figure 18, Table 10), resembling trends observed for formulation **I**. However, this thermal behavior does not correlate with the TGA data. Notably, the 0/100 sample exhibited an anomalous shift in the endothermic transition range, suggesting a distinct thermal response for the fully brominated binder.

### 3.3. Morphology and Chemical Composition of the Char Layer

The above data on the correlation between fire-retardant performance and the self-extinguishing behavior of IFP coatings for formulations **I** and **II** indicate that the incorporation of brominated resins consistently reduces flammability but negatively affects the fire-protective efficacy of these coatings for steel structures. To elucidate the underlying mechanisms, the morphology of the resulting foamed char was further examined using SEM, and the thermo-oxidative decomposition processes of the IFP coatings were investigated using IR spectroscopy.

SEM images of the foamed char layer (Figure 19 and Figures S45–S62) clearly show that replacing the non-brominated NPEL-128 resin with the brominated NPEB-400 resin alters the morphology of the char formed upon thermal exposure of the original coating. The porosity of the coating increases, and the pore size distribution becomes uneven. At the macroscopic level, a high content of brominated resin leads to non-uniform expansion of the fire-protective coating under thermal shock and the formation of large voids and caverns in the foamed char (Figures S69 and S70), which reduce the structural integrity of the char under prolonged thermal exposure and ultimately decrease the fire-protective performance of the coating.

**Figure 20:**
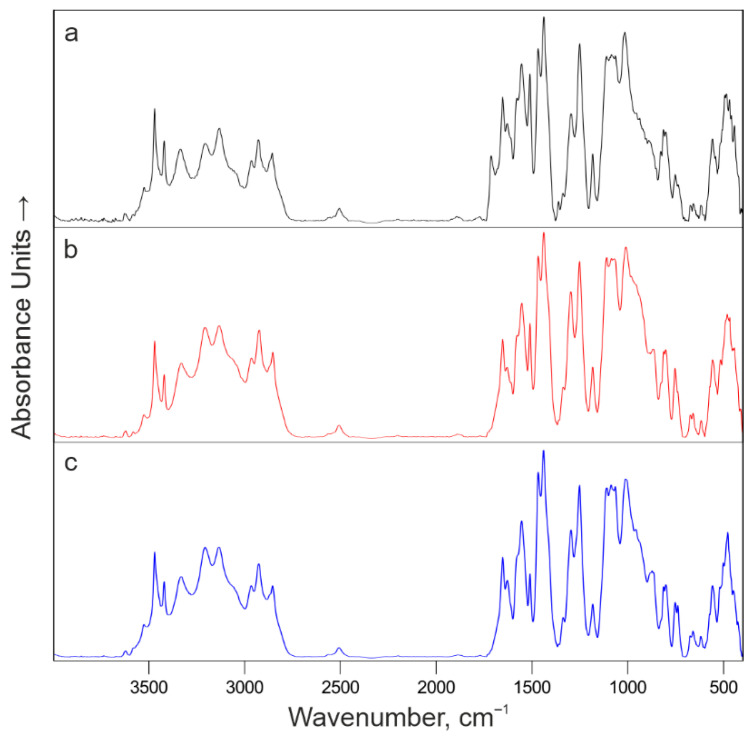
FTIR spectra of unexposed IFP coatings of formulation **I** with different NPEL-128/NPEB-400 ratios: (**a**) 100/0 (black), (**b**) 75/25 (red), and (**c**) 50/50 (blue).

To investigate the influence of bromine content on the thermo-oxidative degradation of the coatings, FTIR spectra were recorded for both the original IFP coatings and the resulting foamed char layers across a series of samples with varying brominated resin content of formulation **I**. The spectra of the unexposed coatings (Figure 20 and Figures S63–S65) are very similar, as the characteristic weak signals of brominated aromatic fragments in the 500–650 cm^−1^ region overlap with signals from the flame-retardant fillers, specifically ammonium polyphosphate (500–650 cm^−1^), titanium dioxide (450–650 cm^−1^), and aluminosilicate fibers (450–600 cm^−1^).

**Figure 21:**
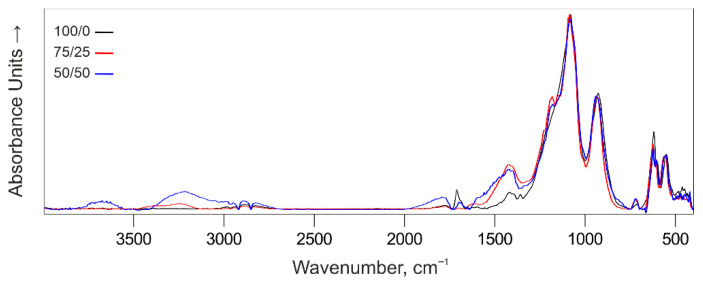
FTIR spectra of foamed char layers formed from IFP coatings of formulation **I** with different NPEL-128/NPEB-400 ratios: (a) 100/0 (black), (b) 75/25 (red), and (c) 50/50 (blue).

After thermo-oxidative degradation, the FTIR spectra of foamed char layers show distinct differences (Figure 21 and Figures S66–S68). In particular, with increasing brominated resin content, the intensity of signals in the 1400–1550, 2900–3400, and 3550–3750 cm^−1^ regions increases, indicating the formation of compounds containing oxygen- and/or nitrogen-containing groups (e.g., –OH, –COOH, –NH). Specifically, signals in the 1400–1550 cm^−1^ range correspond to symmetric and asymmetric vibrations of R–COO^−^ groups and N–H bending of amide linkages, while those in the 2900–3400 cm^−1^ range correspond to stretching vibrations of O–H and N–H bonds.

This behavior is likely associated with a change in the thermo-oxidative degradation mechanism of the resin with increasing bromine content, shifting toward radical-mediated pathways. Bromine radicals inhibit combustion in the gas phase, but simultaneously alter the pyrolysis of the condensed phase, promoting deeper oxidation of the polymer backbone. This leads to the formation of higher amounts of alcohols, phenols, and carboxylic acids in the char, which give rise to the intense O–H bands observed in the FTIR spectra.

Consequently, the foamed char derived from the brominated resin is likely more oxygenated, porous, and less graphitic, whereas the halogen-free NPEL-128 resin produces a more ordered, condensed char layer with fewer surface oxygen-containing functional groups. Its decomposition resembles “pure” pyrolysis with carbonization, forming a more graphitic, structurally uniform char.

## 4. Conclusions

This study demonstrates that partial replacement of diane epoxy resin in intumescent fire-protective coatings with a brominated analogue significantly affects coating flammability and fire-protective performance. For coatings that initially support flame propagation, the introduction of brominated epoxy resin imparts self-extinguishing behavior after flame removal. When the substitution level does not exceed 50%, this modification does not result in a substantial loss of fire-protective efficacy, indicating the presence of an optimal brominated resin content for balancing flame suppression and thermal protection.

For formulations that are inherently self-extinguishing, substitution of the halogen-free epoxy resin with its brominated analogue leads to a reduction in fire-protective performance. Thermal analysis and spectroscopic data show that brominated epoxy resins exhibit lower thermal stability and altered degradation pathways, promoting increased oxidation of the polymer matrix. As confirmed by SEM, these changes result in the formation of a less continuous and more irregularly porous foamed char with reduced mechanical integrity and thermal insulation capacity.

Overall, brominated epoxy resins exert a multidirectional influence on the behavior of intumescent coatings under fire exposure. While bromine-containing binders enhance self-extinguishing properties through gas-phase flame inhibition, they simultaneously impair the quality of the protective char formed during prolonged heating, thereby limiting fire-retardant efficacy.

From a practical perspective, partial substitution of epoxy resin with a brominated analogue is most suitable for intumescent coatings designed to provide moderate fire resistance (approximately 30–90 min). In this range, self-extinguishing behavior and improved adhesion to steel substrates can be achieved without increasing the total content of flame-retardant additives, which often adversely affects mechanical properties and durability. For applications requiring extended fire resistance (≥90 min), epoxy binders with a higher tendency toward graphitization are preferable, and the use of brominated epoxy resins is unlikely to be beneficial due to their negative impact on char continuity and stability.

## Figures and Tables

**Figure 4:**
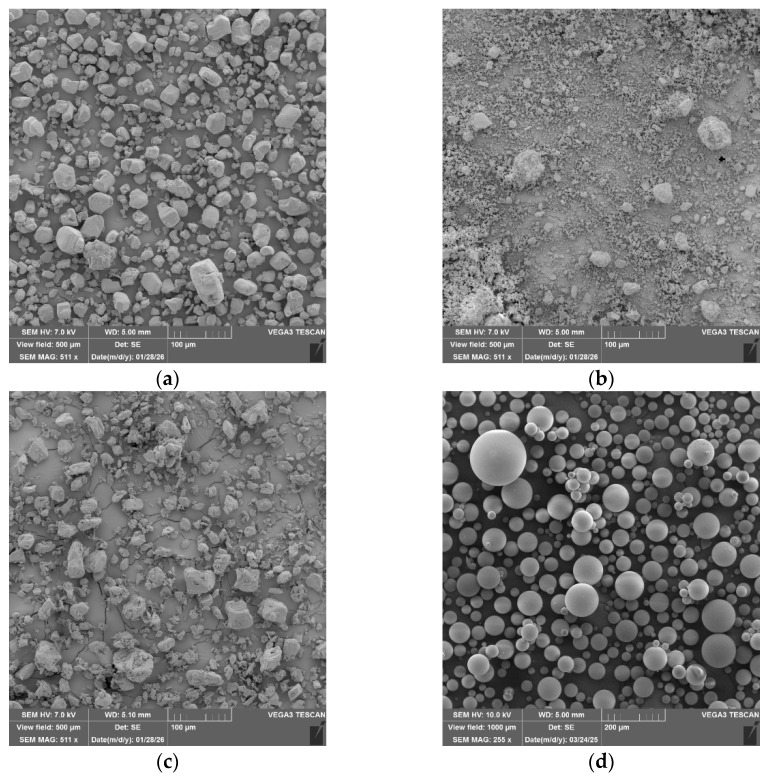
SEM images of the starting powder components used for the preparation of IFP formulations: (**a**) ammonium polyphosphate, (**b**) aluminum hydroxide, (**c**) melamine, and (**d**) glass microspheres.

**Figure 19:**
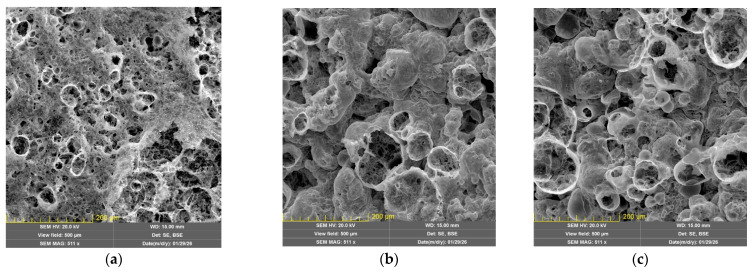
SEM images of the foamed char layer from formulation **I**, showing the effect of replacing halogen-free NPEL-128 resin with brominated NPEB-400 resin on pore structure and morphology ((**a**)—100/0, (**b**)—75/25, (**c**)—50/50).

**Table 1:** Technical characteristics of NPEL-128 epoxy resin.

Characteristic	Value
Physical state	Liquid
Epoxide equivalent weight (EEW), g/equiv	184–190
Viscosity (25 °C), cP	12,000–15,000
Hydrolyzed chlorine content, ppm	<1000

**Table 2:** Technical characteristics of NPEB-400 epoxy resin.

Characteristic	Value
Physical state	Solid
Epoxide equivalent weight (EEW), g/equiv	380–410
Softening point, °C	64–74
Hydrolyzed chlorine content, ppm	<200
Bromine content, %	46–50
Density (25 °C), g/cm^3^	1.81

**Table 3:** Composition of the first component (**A**—base) of the initial bromine-free formulations, **I** and **II**, based on NPEL-128 diane resin.

Ingredients	Content, wt. %	
	Formulation IA	Formulation IIA
Epoxy resin NPEL-128 (187 g/equiv)	21.6	27.2
Reactive diluent (280 g/equiv)	5.1	–
Reactive diluent (149 g/equiv)	–	5.4
Reactive diluent (236 g/equiv)	–	5.4
Ammonium polyphosphate	16.7	30.5
Zinc borate	21.6	9.7
Titanium dioxide	0.7	5.0
Chopped aluminosilicate fiber	5.9	4.4
Melamine	5.6	4.5
Aluminum hydroxide	2.4	–
Glass microspheres	8.4	–
Carbon fiber, 3 mm	–	1.5
Ethyl acetate	3.9	–
Functional additives	8.1	6.4
∑	100.0	100.0

**Table 4:** Composition of the second component (**B**—hardener) of formulations **I** and **II**.

Ingredients	Content, wt. %	
	Formulation IB	Formulation IIB
Hardener polyetheramine (60 g/equiv)	33.3	34.8
Hardener Mannich base (95 g/equiv)	66.7	–
Ammonium polyphosphate	–	14.5
Titanium dioxide	–	0.7
Pentaerythritol	–	47.1
Functional additives	–	2.9
∑	100.0	100.0

**Table 5:** Ratio of NPEL-128/NPEB-400 resins in formulations **IA** and **IIA**.

Parameter	Ratio					
Equivalent, %	100/0	87.5/12.5	75/25	50/50	25/75	0/100
Mass, %	100/0	76.8/23.2	58.6/41.3	32.1/67.9	13.6/86.4	0/100

**Table 6:** Characteristics of compounds based on NPEL-128 epoxy resin.

Characteristic	Formulation I	Formulation II
Self-extinguishing after removal from the combustion zone	–	+
Fire-retardant efficacy *, min	72 ± 2	74 ± 2
Pull-off force from substrate, MPa	4.5 ± 0.5	7 ± 1
Expansion coefficient	1.8 ± 0.1	17 ± 1

*—normalized to dry coating thickness 6.5 mm.

**Table 7:** The results for IFP coatings obtained from formulation **I**.

Characteristic	Value					
NPEL-128/NPEB-400 ratio *	100/0	87.5/12.5	75/25	50/50	25/75	0/100
Br content in the compound (% by weight)	0.0	2.4–2.5	4.6–4.8	8.8–9.2	12.7–13.2	16.1–16.8
Self-extinguishing ability						
Sum of *T_H_* for a series of 3 samples, s	0	0	0	0	0	0
Sum of *T_V_*_1_ for a series of 5 samples, s	0	0	0	0	0	0
Sum of *T_V_*_2_ for a series of 5 samples, s	259 ± 5	0	0	0	0	0
Sum of *T_V_*_3_ for a series of 5 samples, s	0	0	0	0	0	0
UL-94 rating	–	V0	V0	V0	V0	V0
Fire-retardant efficacy						
Fire-retardant efficacy **, min	72 ± 2	69 ± 2	69 ± 2	56 ± 2	52 ± 2	58 ± 2
Adhesion						
Pull-off force from substrate ***, MPa	4.5 ± 0.5	3.5 ± 0.5	5.0 ± 0.5	4.5 ± 0.5	5.5 ± 0.5	4.5 ± 0.5
Thermal expansion degree						
Expansion factor h_1_/h_0_	1.7 ± 0.1	3.3 ± 0.1	2.6 ± 0.1	3.2 ± 0.2	3.9 ± 0.1	5.9 ± 0.2

*—based on the equivalent weight of epoxy groups in the composition. **—normalized to dry fire-protective coating thickness of 6.5 mm; ***—all IFP coatings underwent cohesive failure.

**Table 8:** Characteristic glass transition temperatures of binder samples from formulation **I** with varying NPEL-128/NPEB-400 ratios.

Resin Ratio	100/0	75/25	50\50	25/75	0/100
T_eig_, °C	83	80	78	82	85
T_mg_, °C	89	86	90	92	91
T_efg_, °C	94	92	99	114	114

**Table 9:** The results for IFP coatings obtained from formulation **II**.

Characteristic	Value				
NPEL-128/NPEB-400 ratio *	100/0	75/25	50/50	25/75	0/100
Br content in the compound (% by weight)	0.0	4.8–5.0	9.2–9.6	13.1–13.7	16.7–17.4
Self-extinguishing ability					
Sum of *T_H_* for a series of 3 samples, s	0	0	0	0	0
Sum of *T_V_*_1_ for a series of 5 samples, s	0	0	0	0	0
Sum of *T_V_*_2_ for a series of 5 samples, s	0	0	0	0	0
Sum of *T_V_*_3_ for a series of 5 samples, s	0	0	0	0	0
UL-94 rating	V0	V0	V0	V0	V0
Fire-retardant efficacy					
Fire-retardant efficacy **, min	74 ± 2	59 ± 2	56 ± 2	63 ± 2	62 ± 2
Adhesion					
Pull-off force from substrate ***, MPa	6 ± 1	9 ± 1	7 ± 1	9 ± 1	8 ± 1
Thermal expansion degree					
Expansion factor *h*_1_/*h*_0_	17 ± 1	15.5 ± 1.7	14.0 ± 1.8	9.0 ± 1.9	9.2 ± 1.9

*—based on the equivalent weight of epoxy groups in the composition; **—normalized to dry fire-protective coating thickness of 6.5 mm; ***—all IFP coatings underwent cohesive failure.

**Table 10:** Characteristic glass transition temperatures for binders of formulation **II**.

Resin Ratio	100/0	75/25	50\50	25/75	0/100
T_eig_, °C	79	77	72	66	110
T_mg_, °C	86	91	88	72	116
T_efg_, °C	90	97	100	102	121

## Data Availability

The original contributions presented in this study are included in the article/Supplementary Material. Further inquiries can be directed to the corresponding author.

## Supplementary Materials

The following supporting information can be downloaded at: https://www.mdpi.com/article/10.3390/polym18040484/s1. Additional data on thermal analysis, SEM, and FTIR spectroscopy are available in the online Supplementary Materials accompanying this article.

## Abbreviations

The following abbreviations are used in this manuscript:

APP	Ammonium Polyphosphate
BS	British Standard
DGEBA	Diglycidyl Ether of Bisphenol A
DGETBBA	Diglycidyl Ether of Tetrabromobisphenol A
DSC	Differential Scanning Calorimetry
EEW	Epoxy Equivalent Weight
HB	Horizontal Burning (UL-94 test)
IFP	Intumescent Fire-Protective
ISO	International Organization for Standardization
TGA	Thermogravimetric Analysis
TBBA	Tetrabromobisphenol A
UL	Underwriters Laboratories
VB	Vertical Burning (UL-94 test)
