# Supplementary material for: Effect of Brominated Epoxy Resin Content on Thermophysical and Mechanical Properties of Intumescent Fire-Protective Coatings
Source: Polymers (Basel). 2026 Feb 14;18(4):484. doi: 10.3390/polym18040484 (PMC12944359; doi:10.3390/polym18040484)
Supplement: Supplementary file 1 [file polymers-18-00484-s001.zip › polymers-4125887-supplementary.pdf]

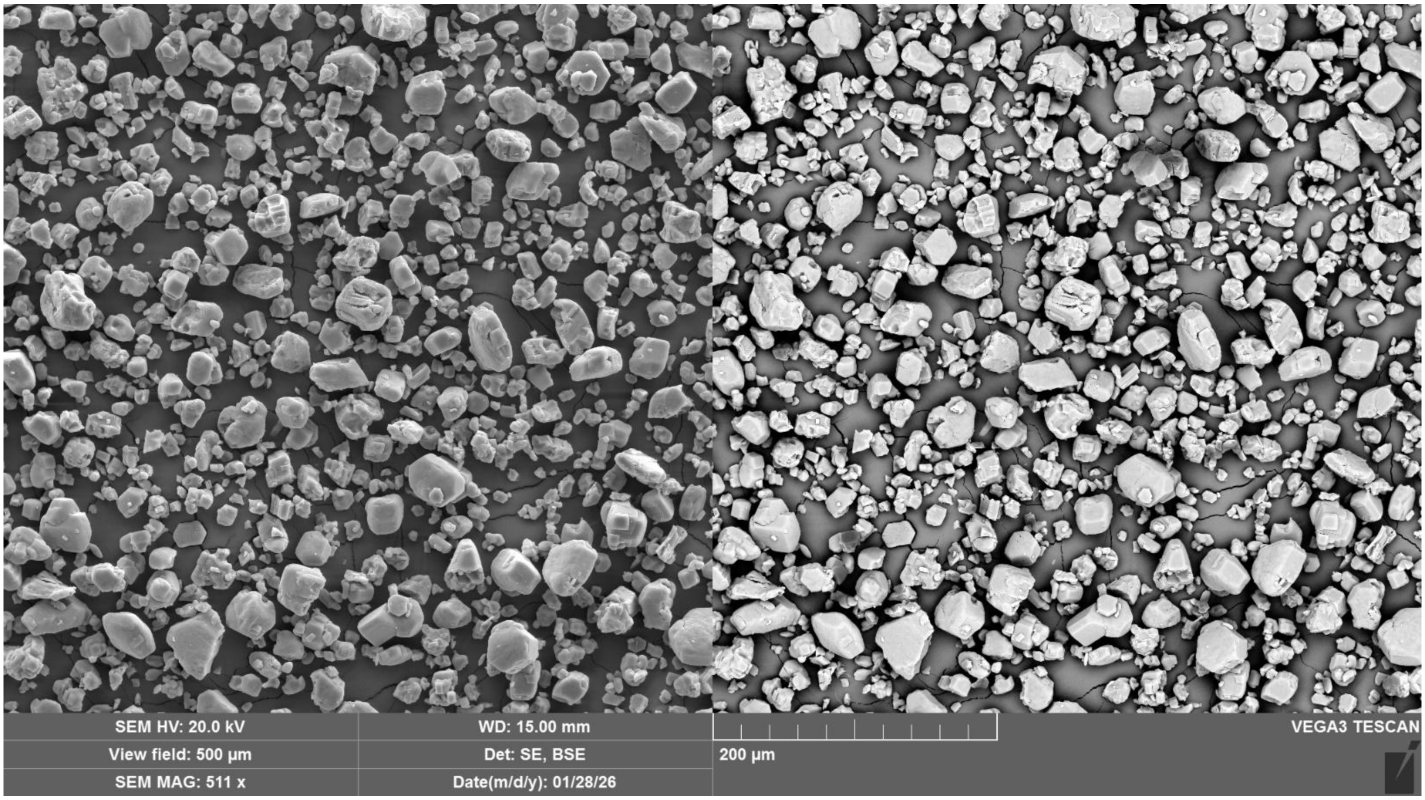

Figure S1. SEM APP

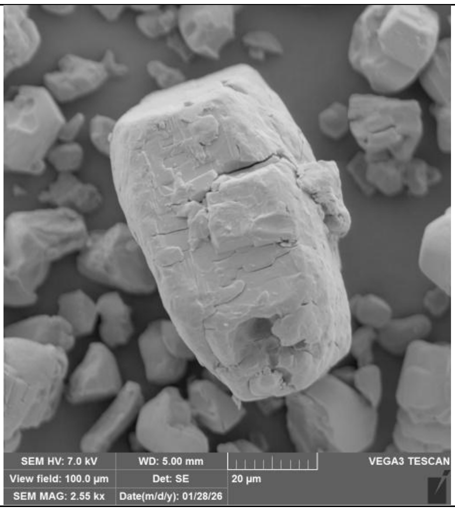

Figure S2. SEM APP

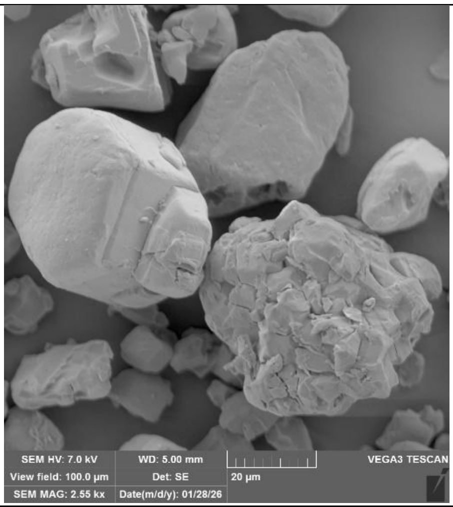

Figure S3. SEM APP

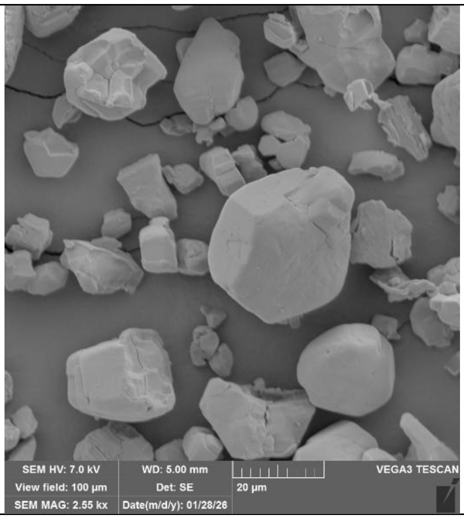

Figure S4. SEM APP

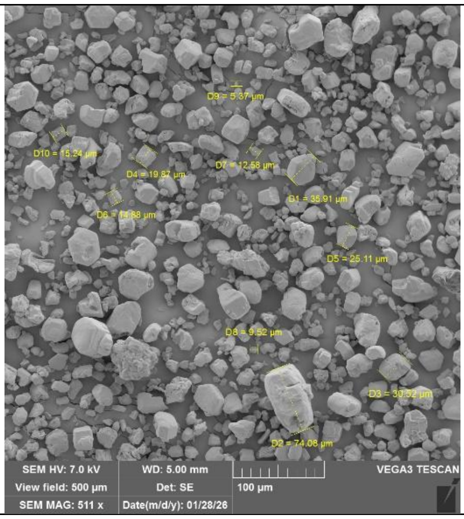

Figure S5. SEM APP

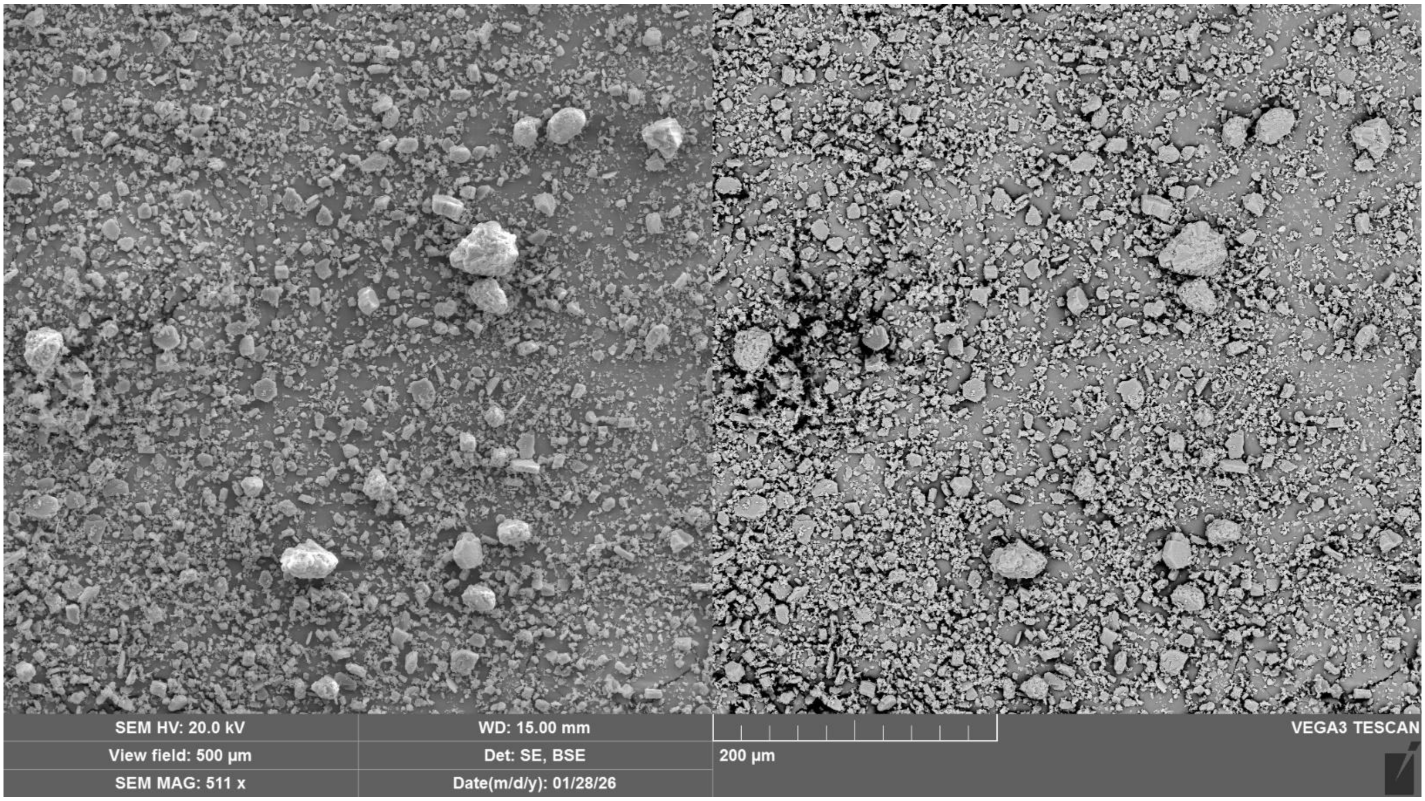

Figure S6. SEM Aluminum hydroxide

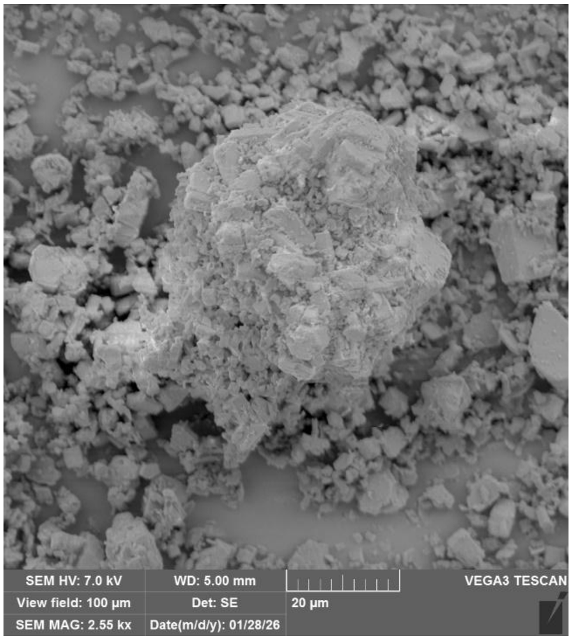

Figure S7. SEM Aluminum hydroxide

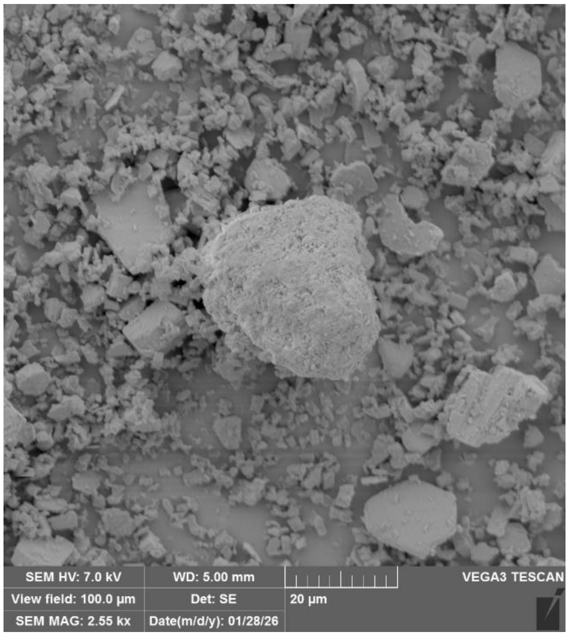

Figure S8. SEM Aluminum hydroxide

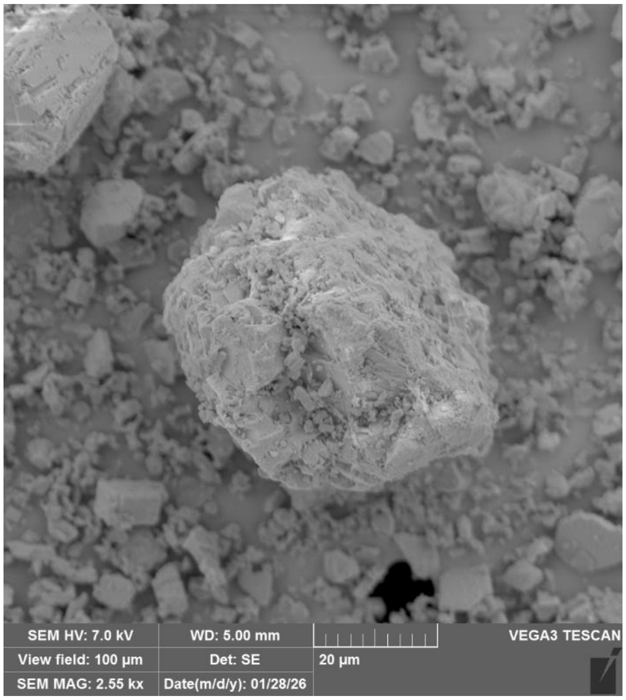

Figure S9. SEM Aluminum hydroxide

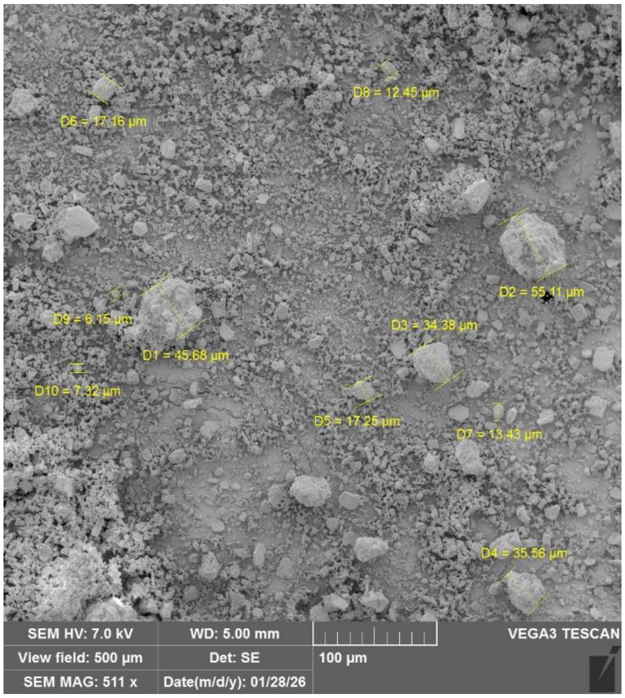

Figure S10. SEM Aluminum hydroxide

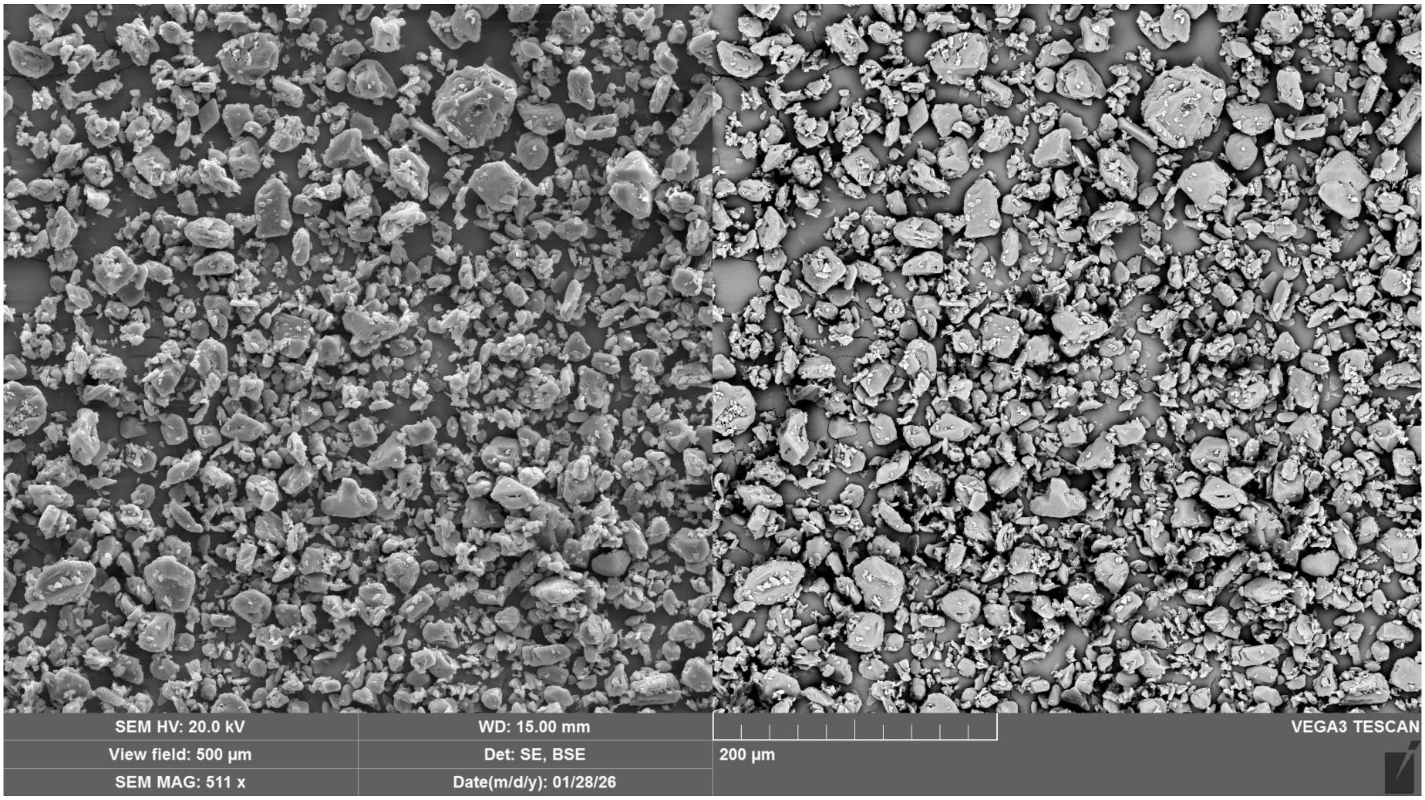

Figure S11. SEM Melamine

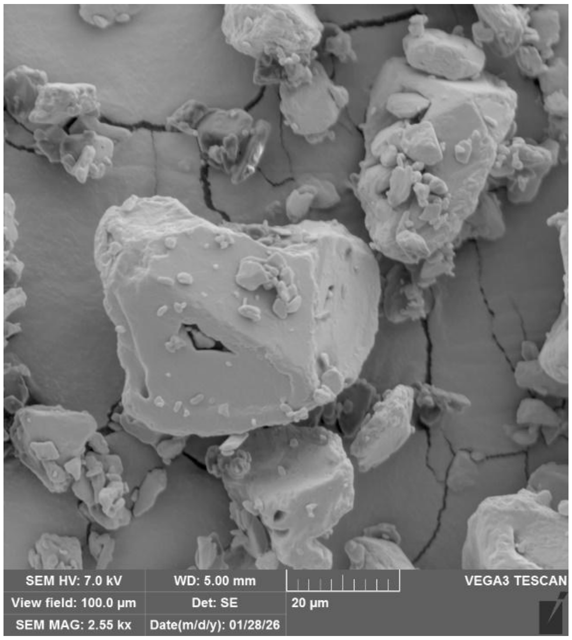

Figure S12. SEM Melamine

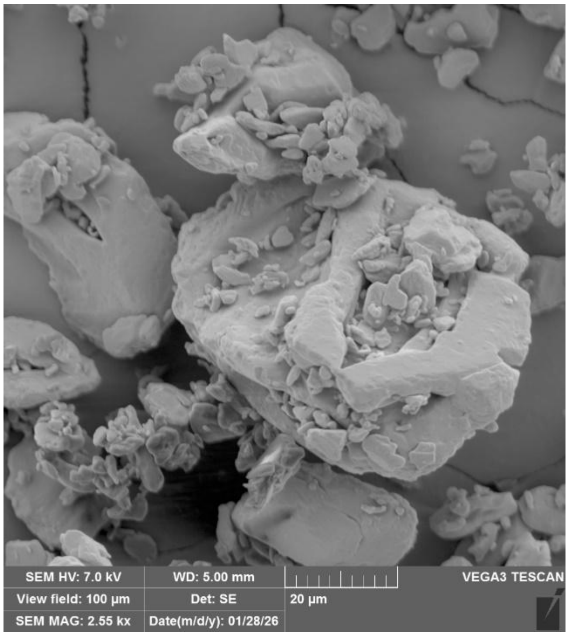

Figure S13. SEM Melamine

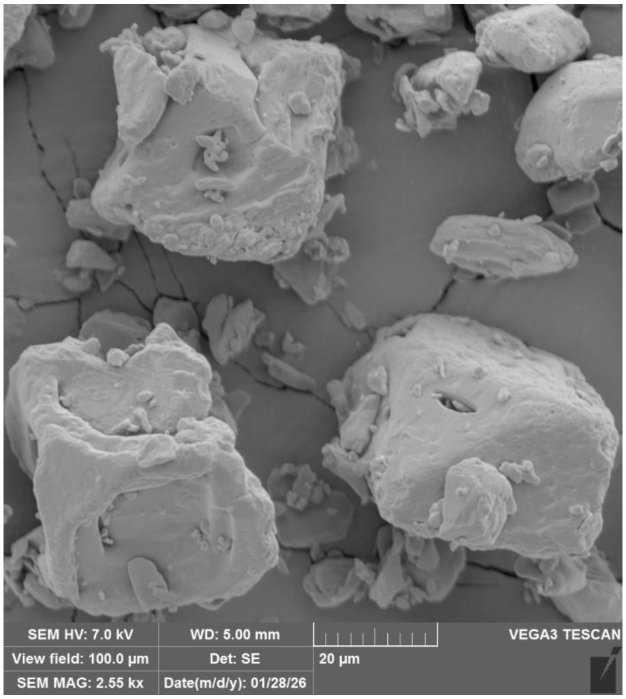

Figure S14. SEM Melamine

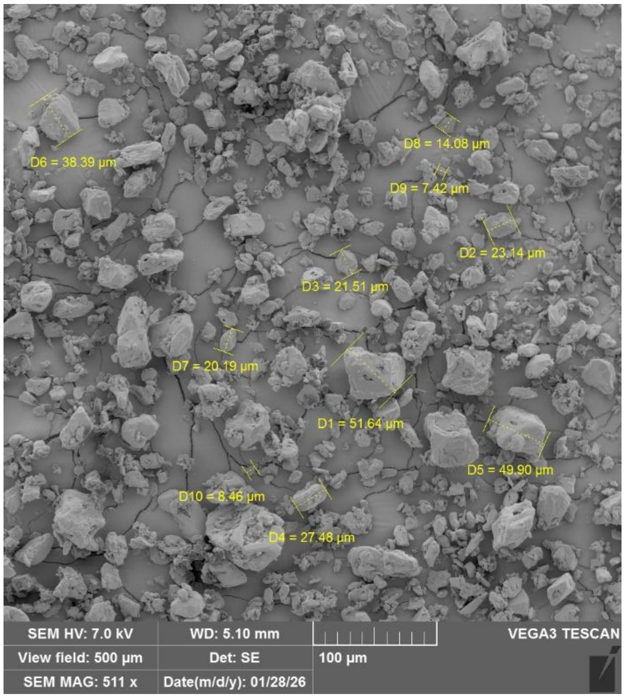

Figure S15. SEM Melamine

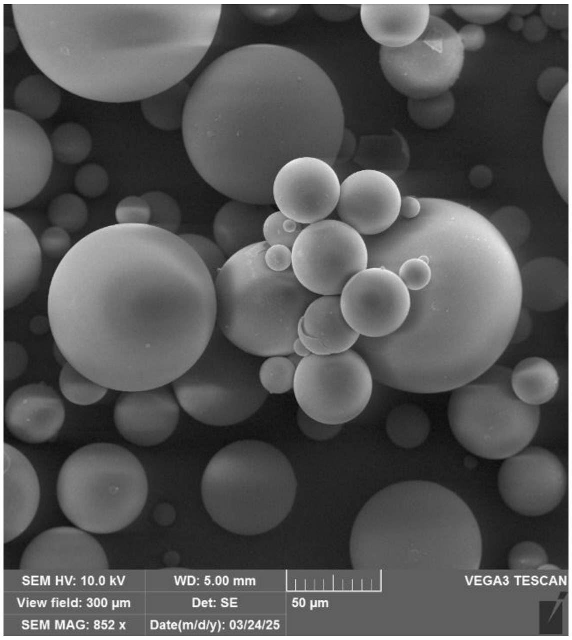

Figure S16. SEM glass microspheres

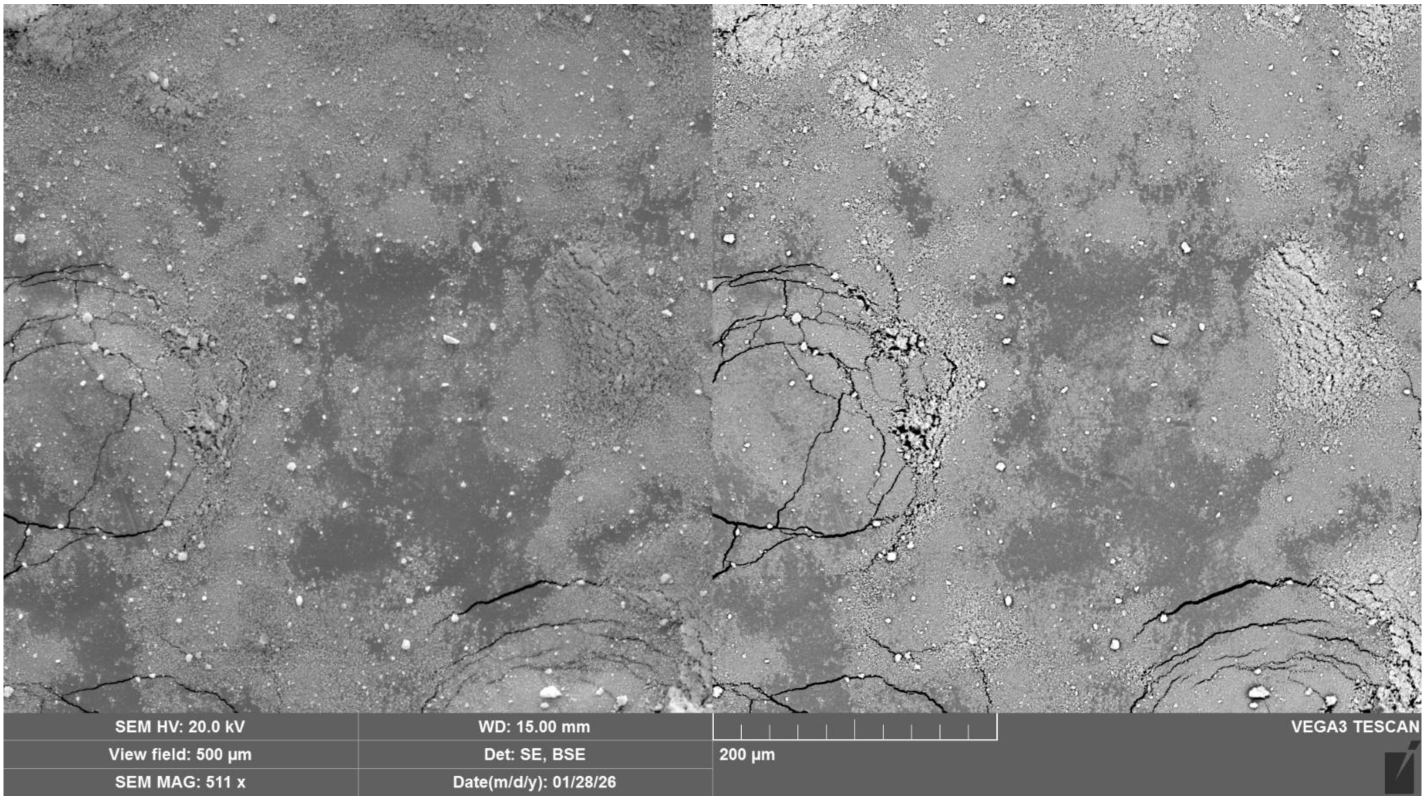

Figure S17. SEM titanium dioxide

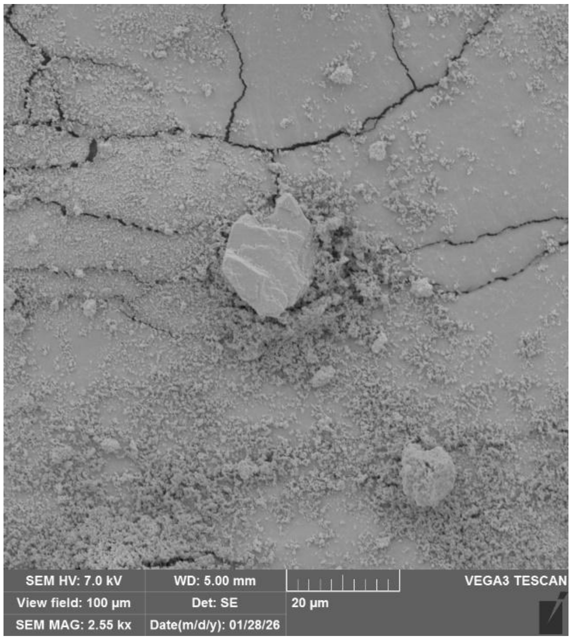

Figure S18. SEM titanium dioxide

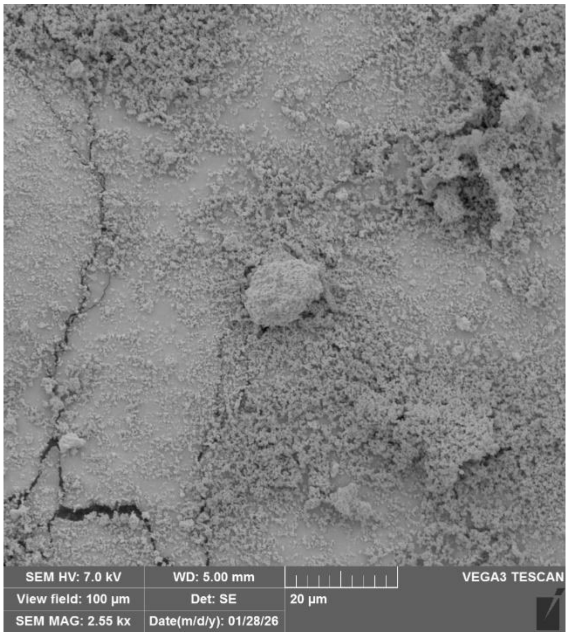

Figure S19. SEM titanium dioxide

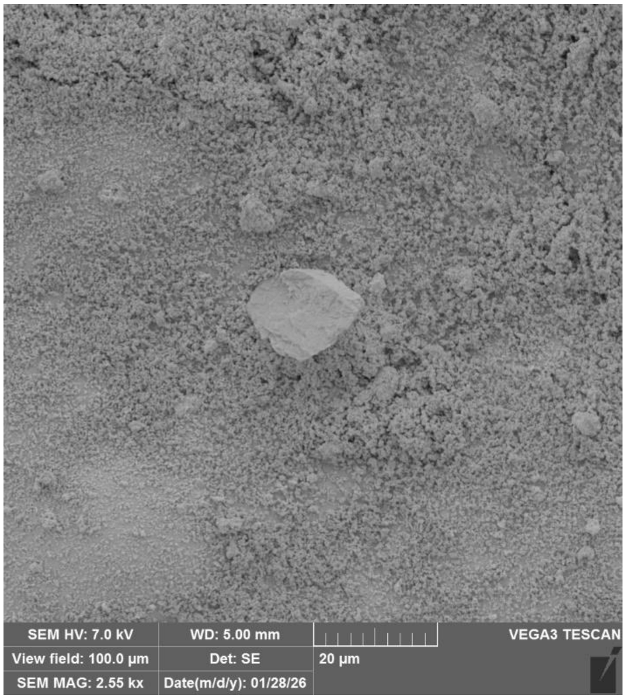

Figure S20. SEM titanium dioxide

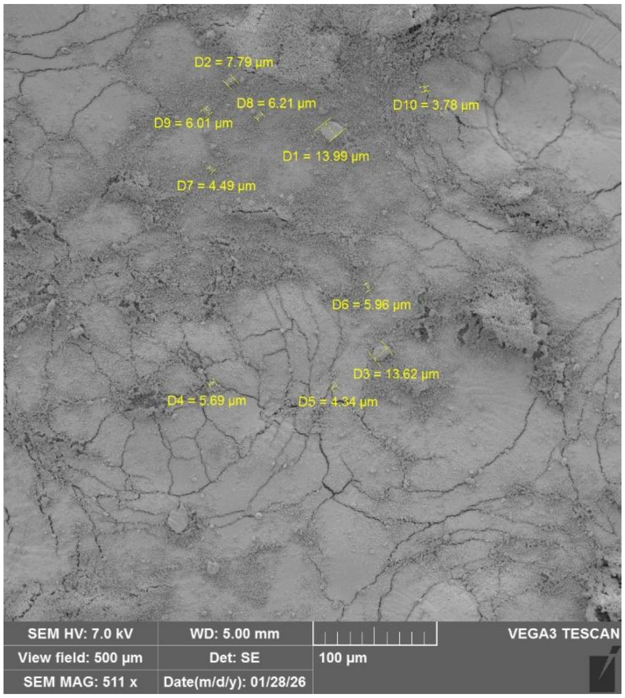

Figure S21. SEM titanium dioxide

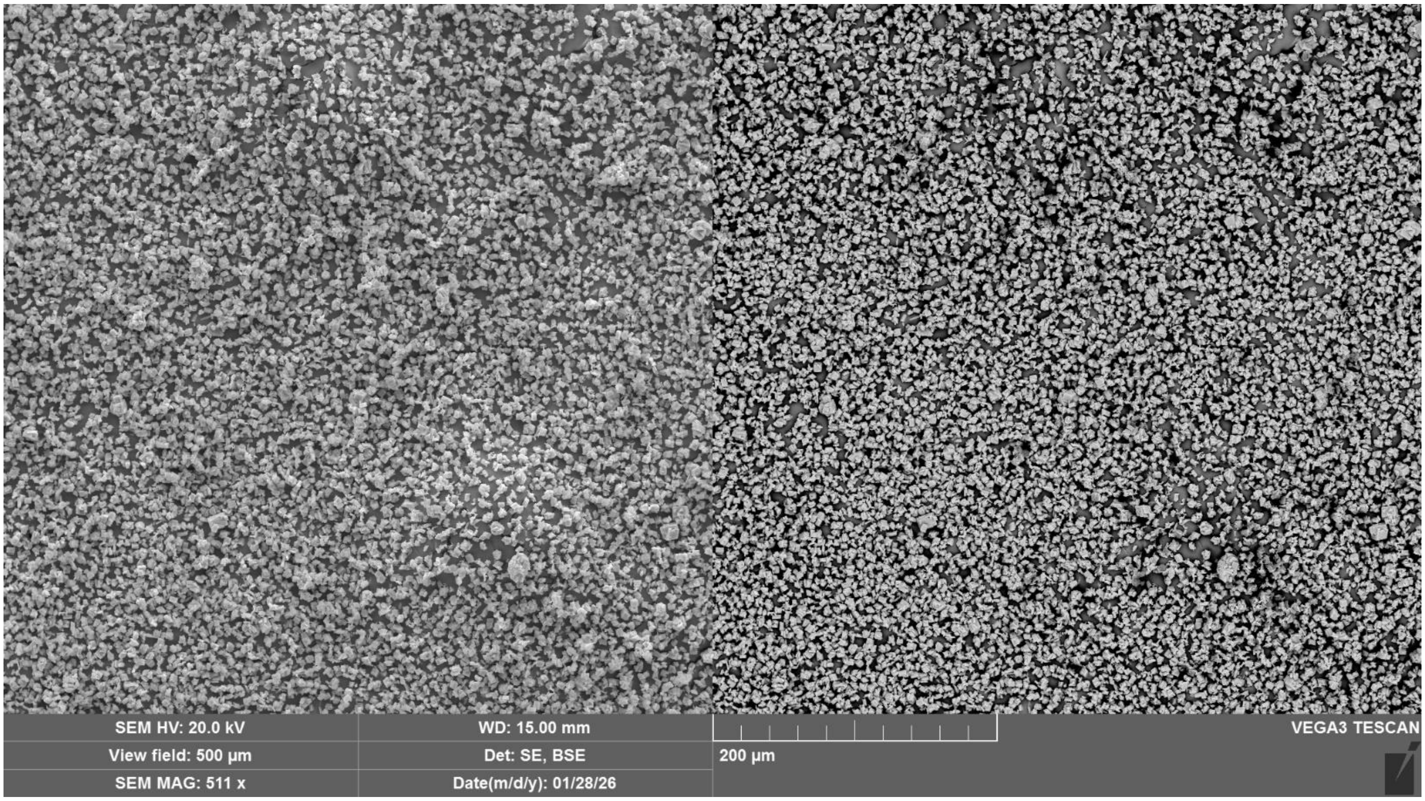

Figure S22. SEM zinc borate

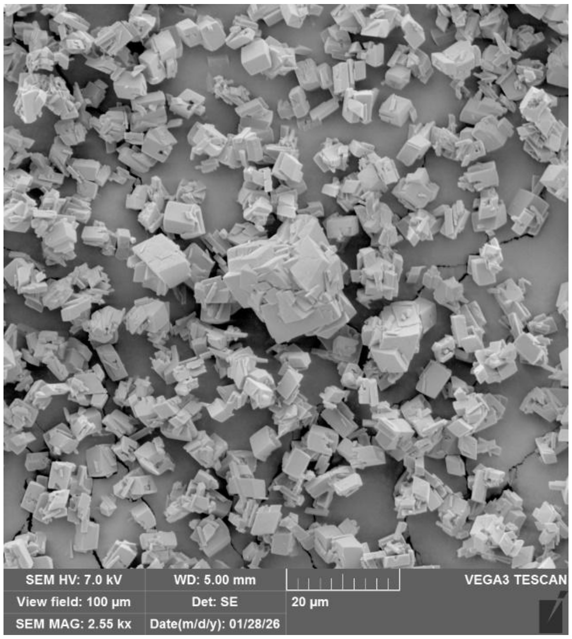

Figure S23. SEM zinc borate

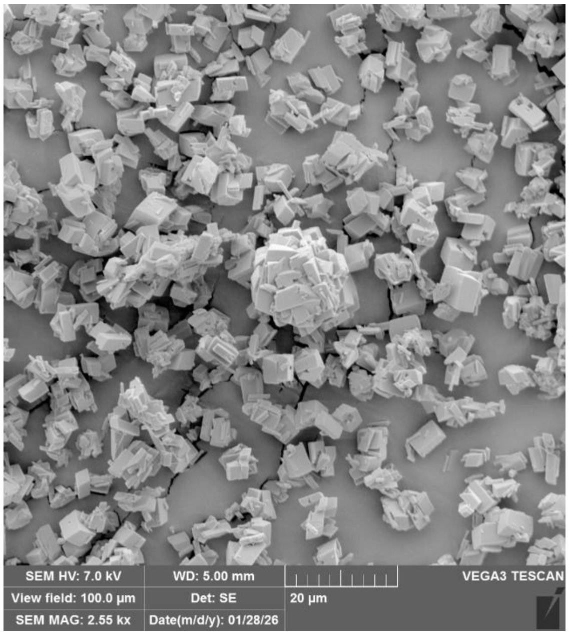

Figure S24. SEM zinc borate

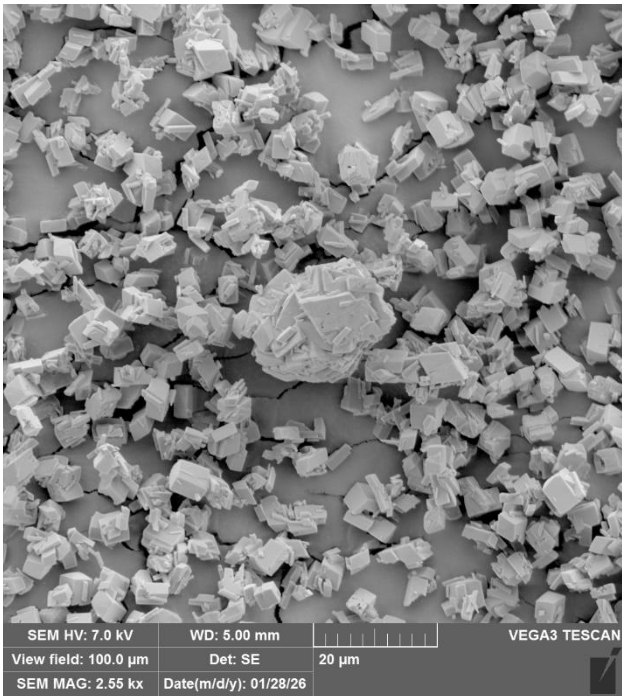

Figure S25. SEM zinc borate

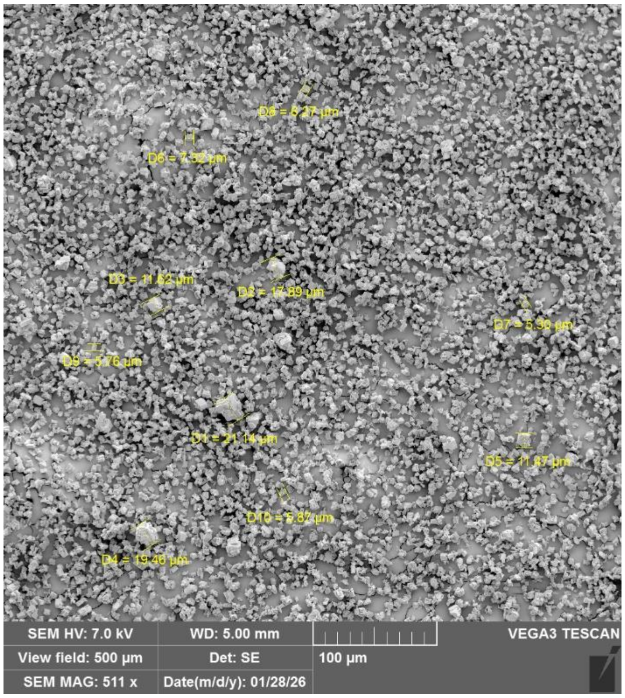

Figure S26. SEM zinc borate

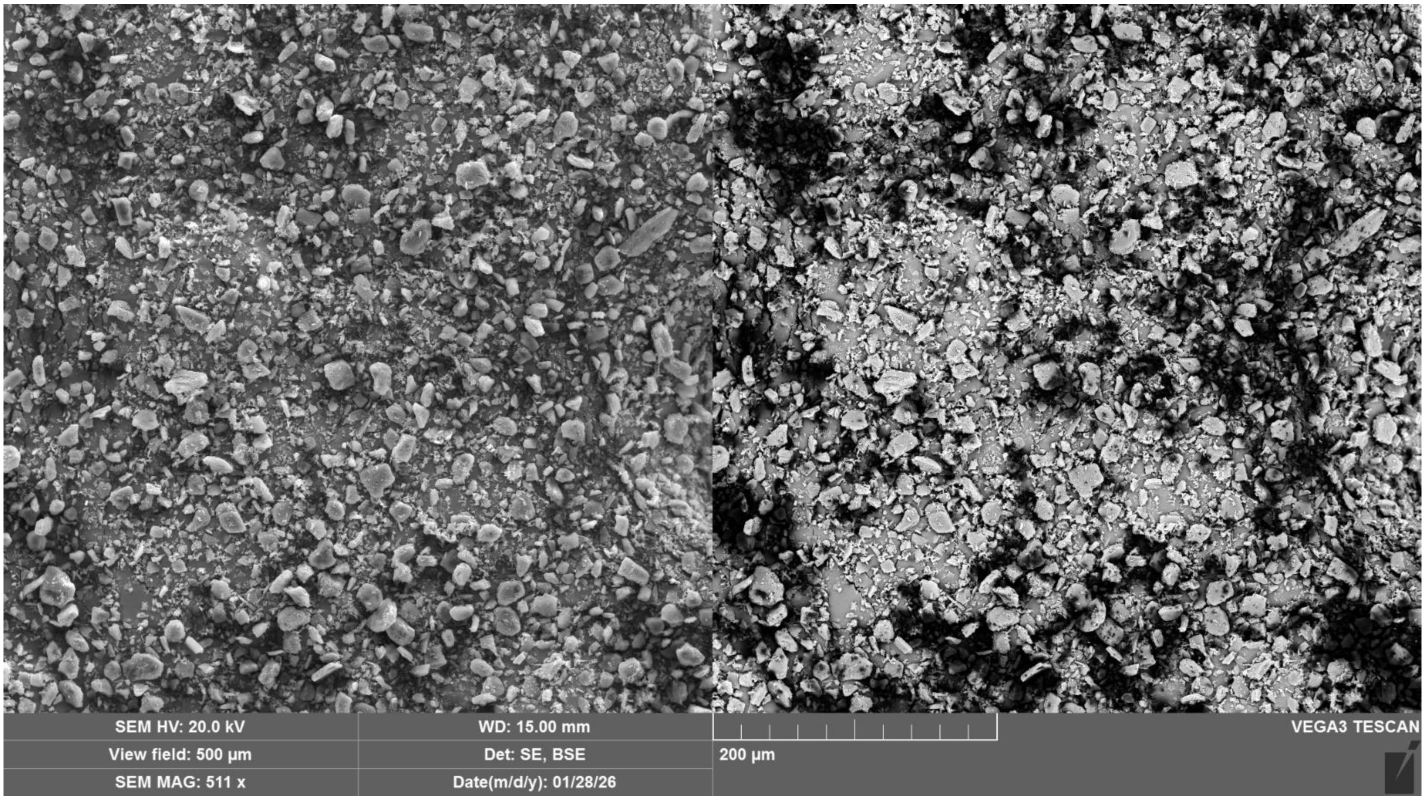

Figure S27. SEM pentaerythritol

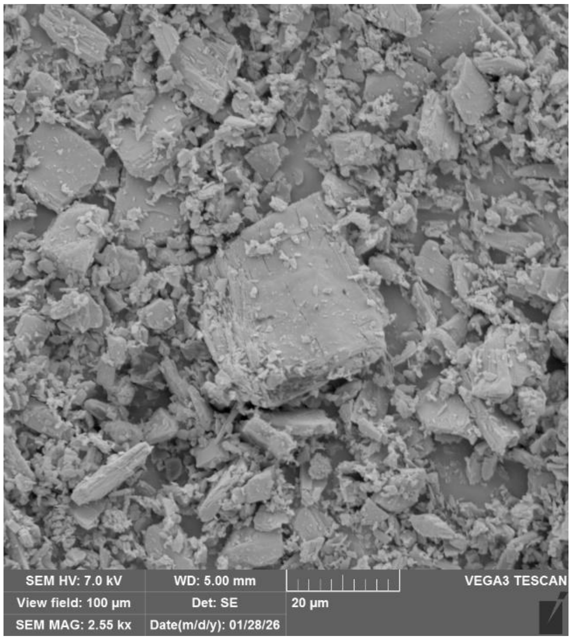

Figure S28. SEM pentaerythritol

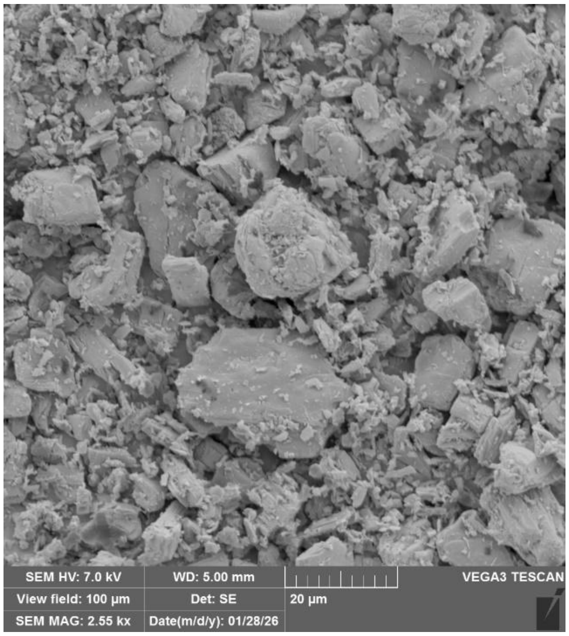

Figure S29. SEM pentaerythritol

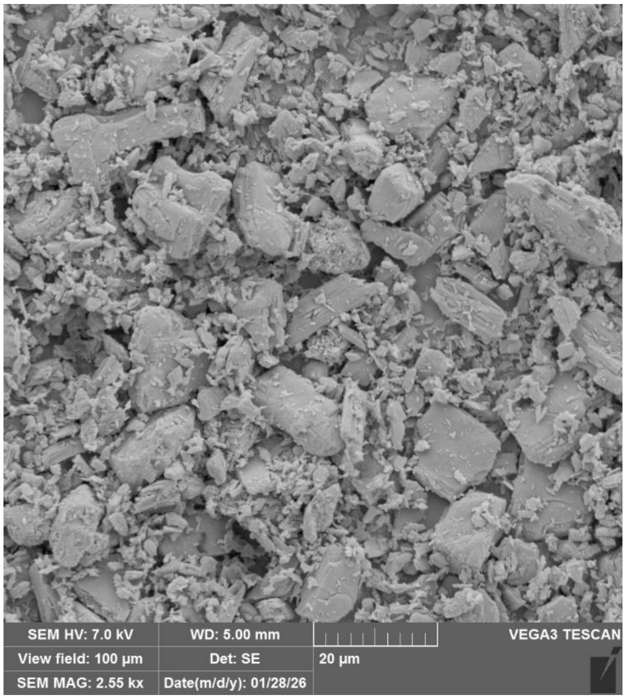

Figure S30. SEM pentaerythritol

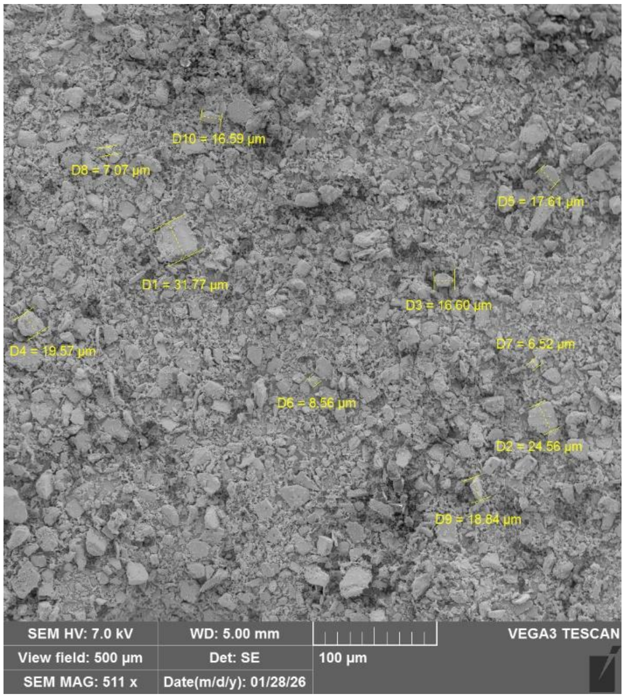

Figure S31. SEM pentaerythritol

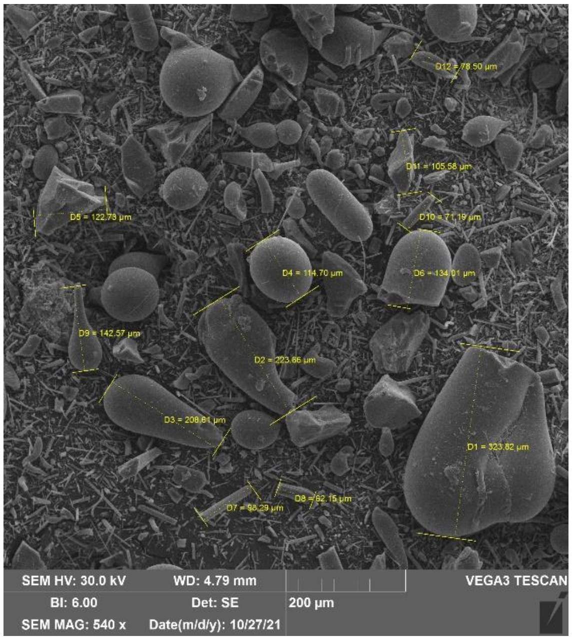

Figure S32. SEM chopped aluminosilicate fiber

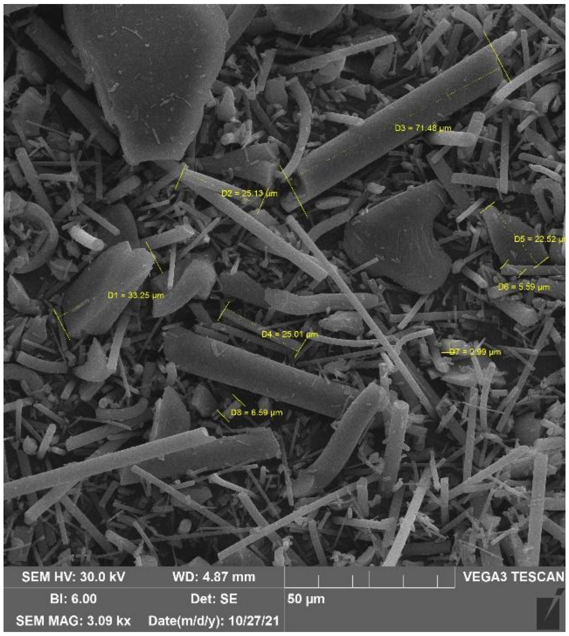

Figure S33. SEM chopped aluminosilicate fiber

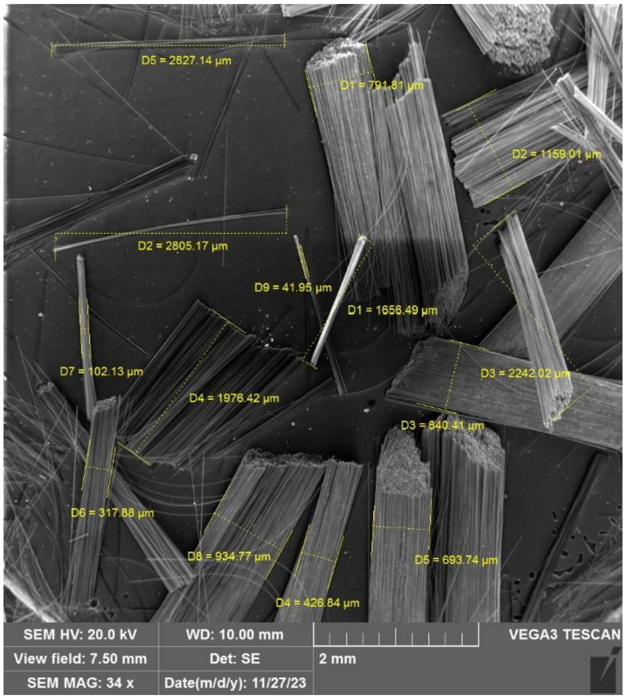

Figure S34. SEM carbon fiber

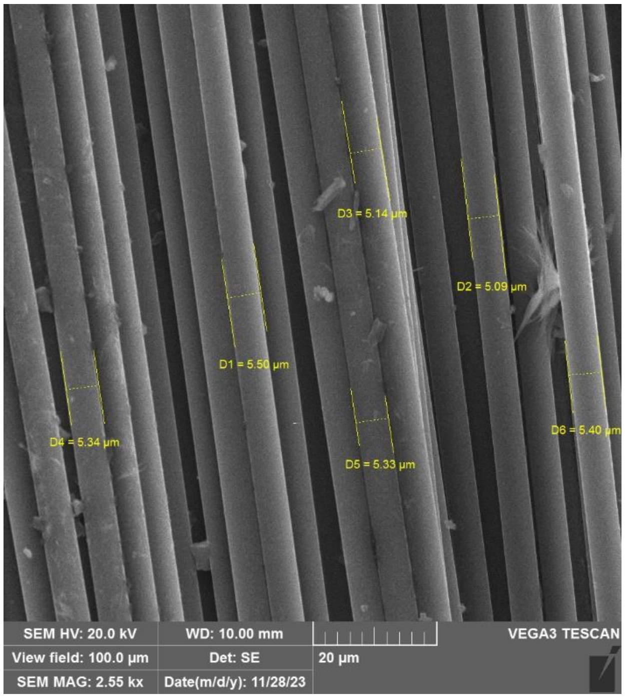

Figure S35. SEM carbon fiber

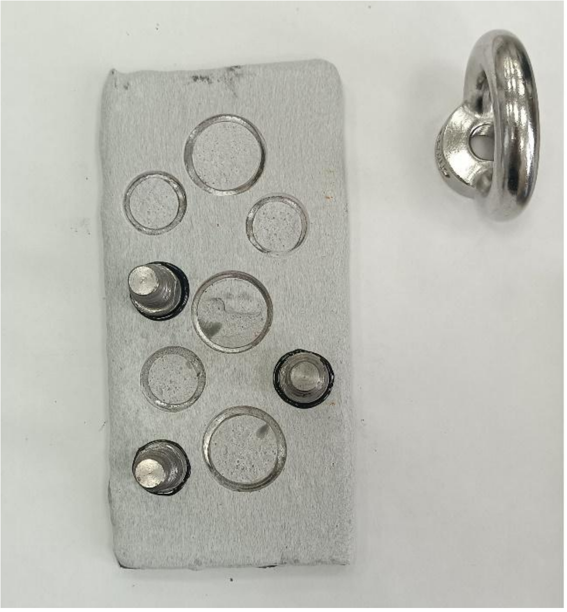

Figure S36. Pull-off adhesion testing sample

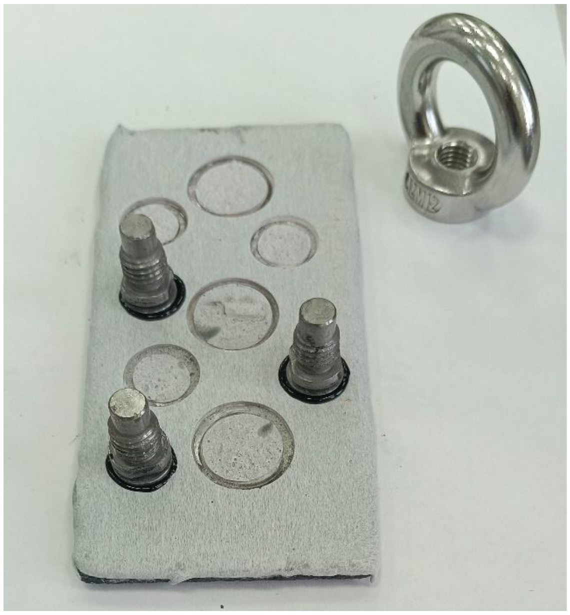

Figure S37. Pull-off adhesion testing sample

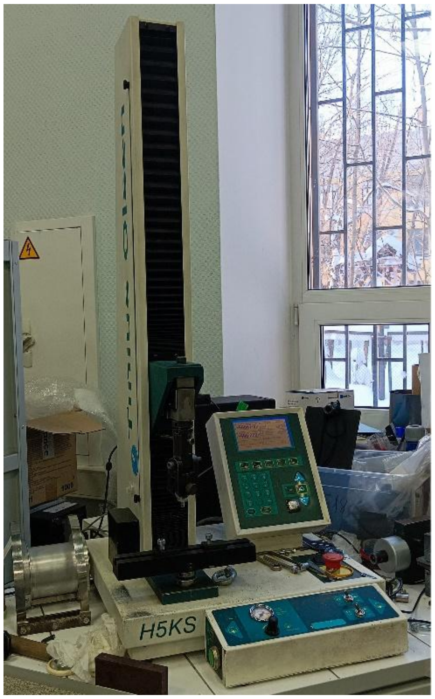

Figure S38. Pull-off adhesion testing machine

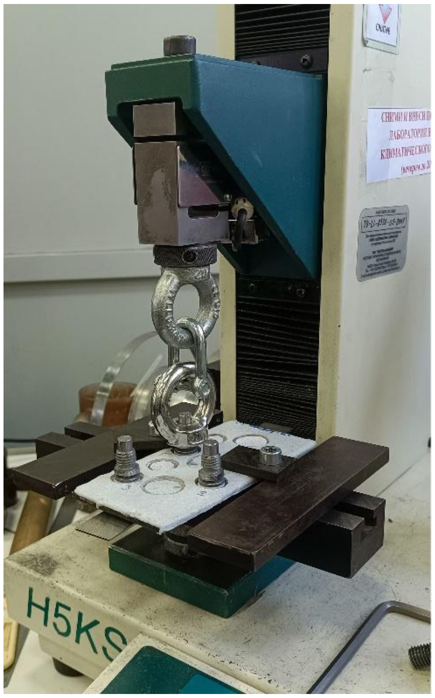

Figure S39. Pull-off adhesion testing setup

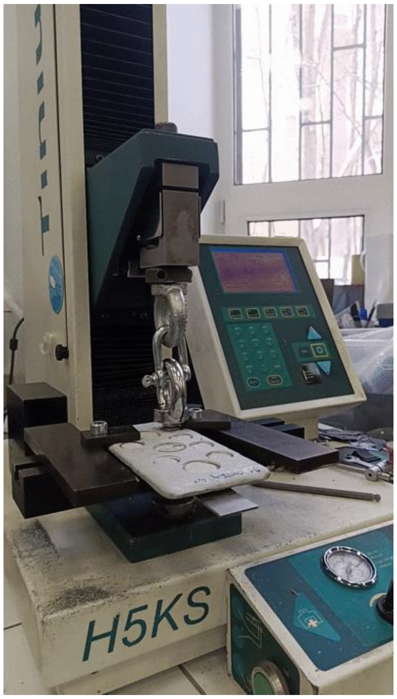

Figure S40. The moment of coating failure during pull-off adhesion testing

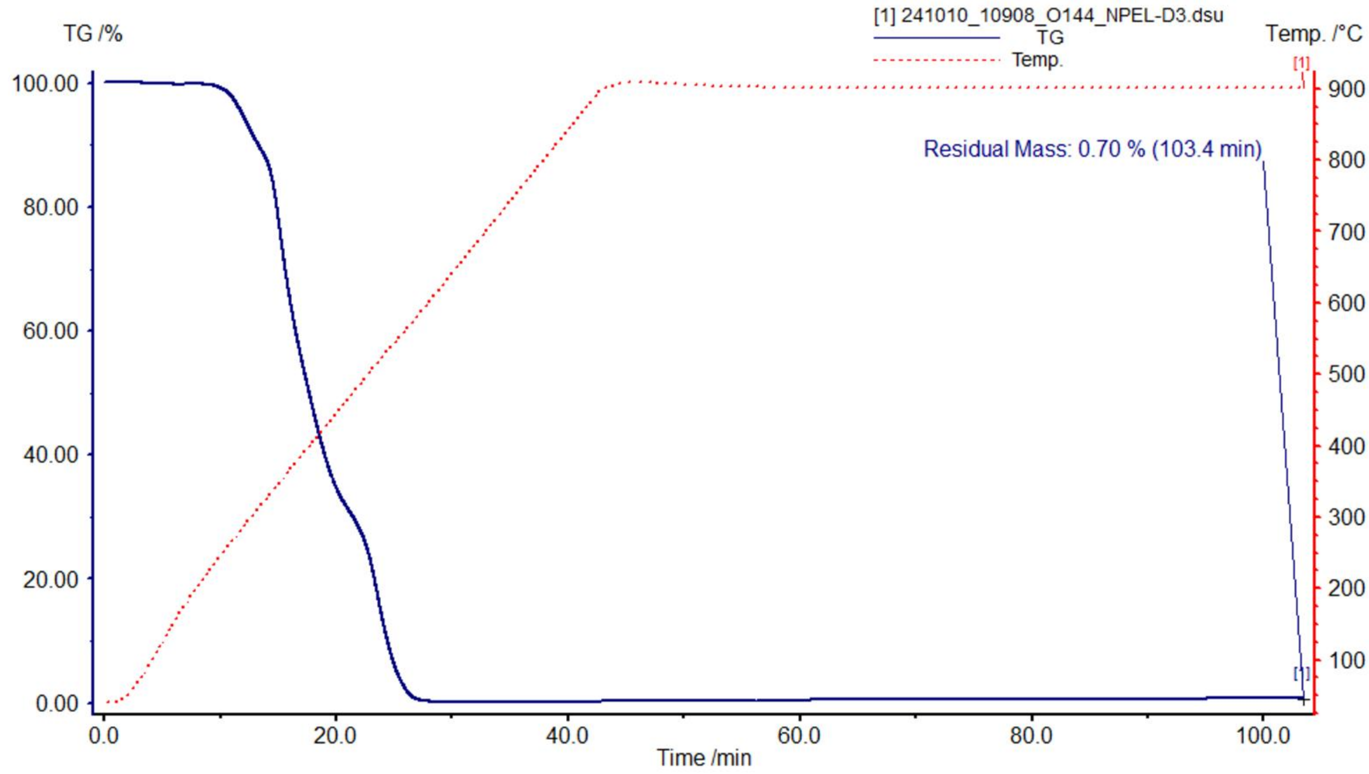

Figure S41. TG-curve of sample formulation **I** NPEL-128/NPEB-400=100/0 at the isothermal section

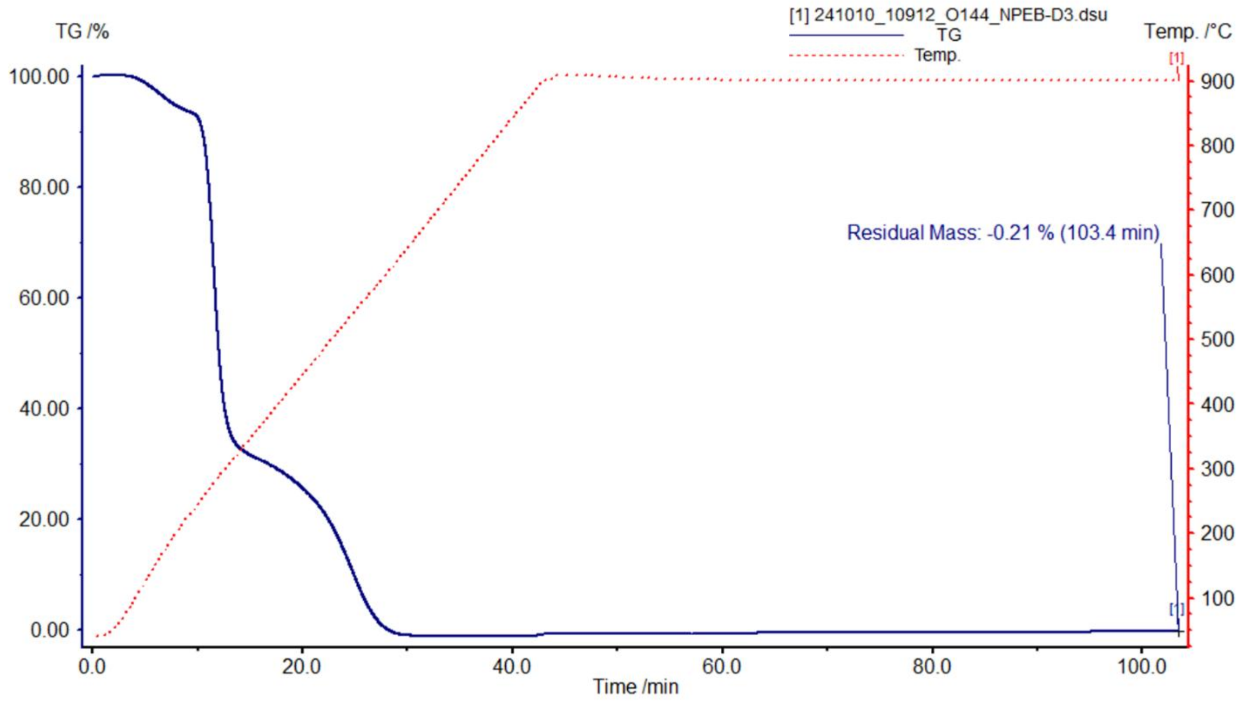

Figure S42. TG-curve of sample formulation **I** NPEL-128/NPEB-400=0/100 at the isothermal section

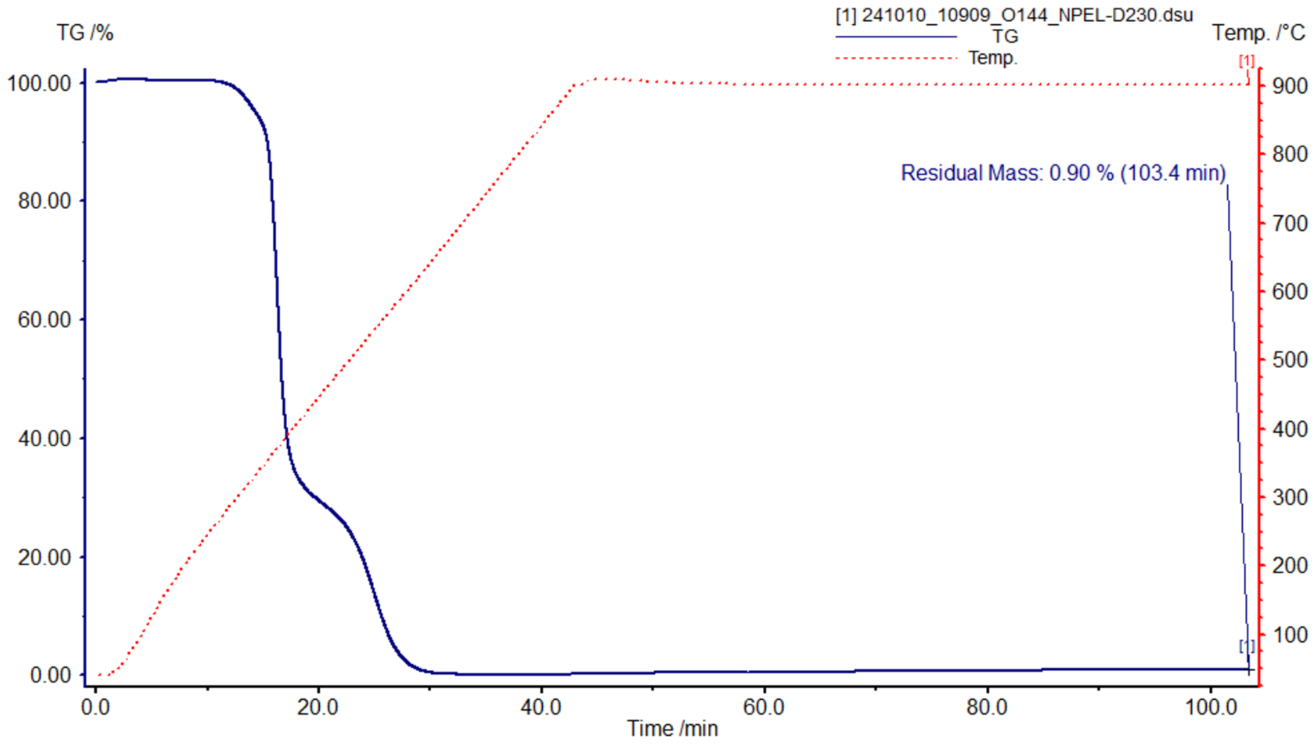

Figure S43. TG-curve of sample formulation **II** NPEL-128/NPEB-400=100/0 at the isothermal section

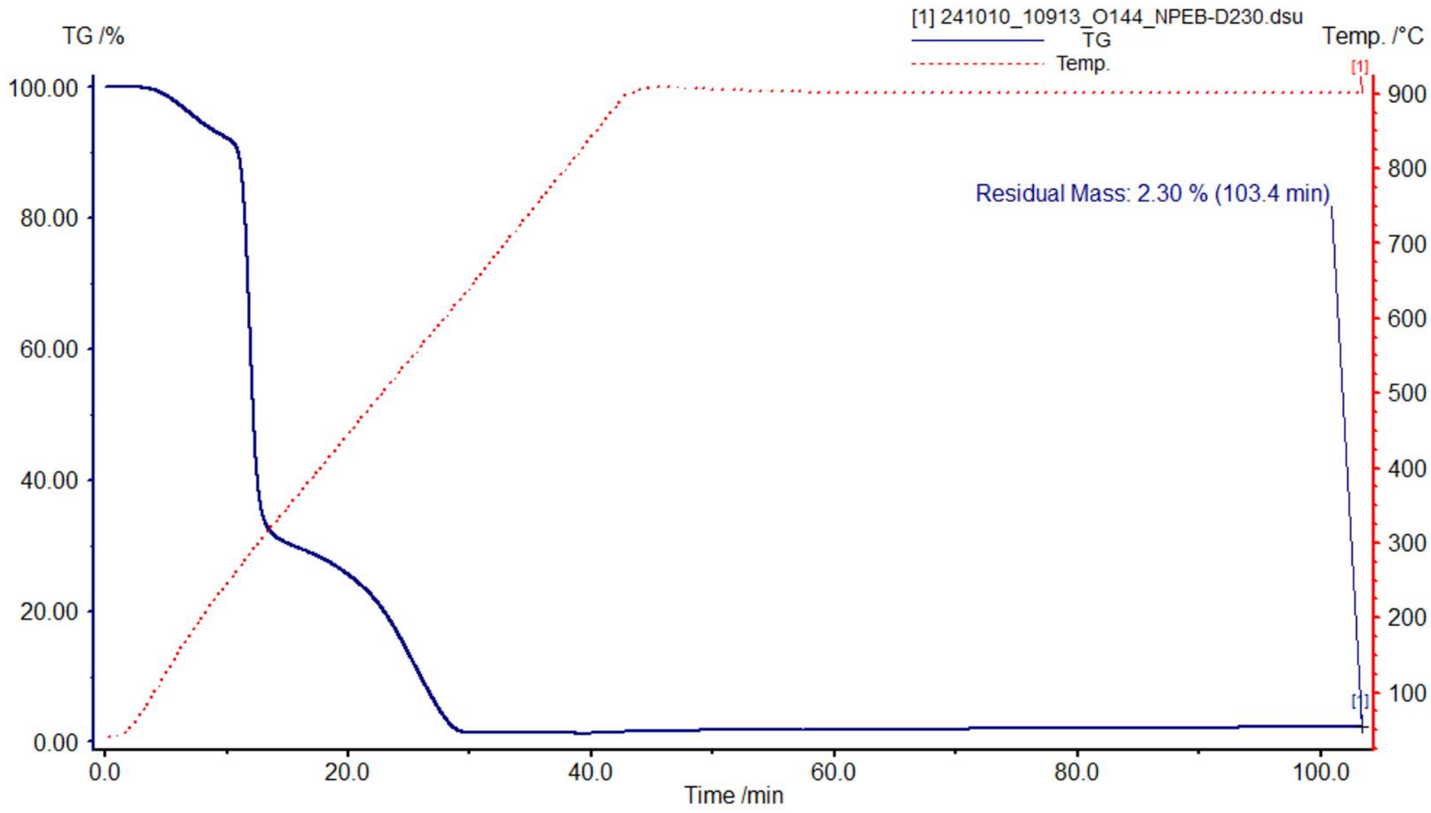

Figure S44. TG-curve of sample formulation **II** NPEL-128/NPEB-400=0/100 at the isothermal section

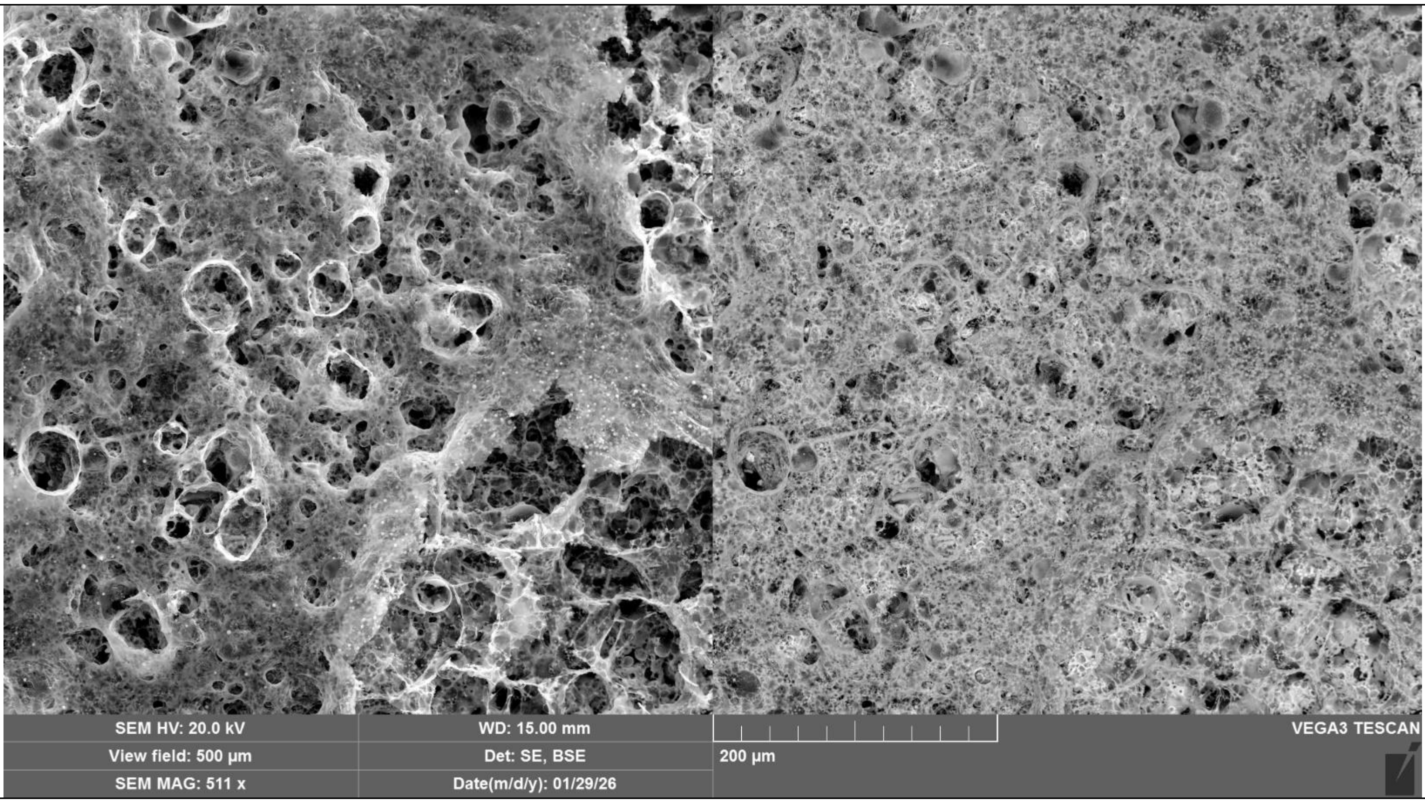

Figure S45. SEM of foamed char layer of formulation I 100/0 sample

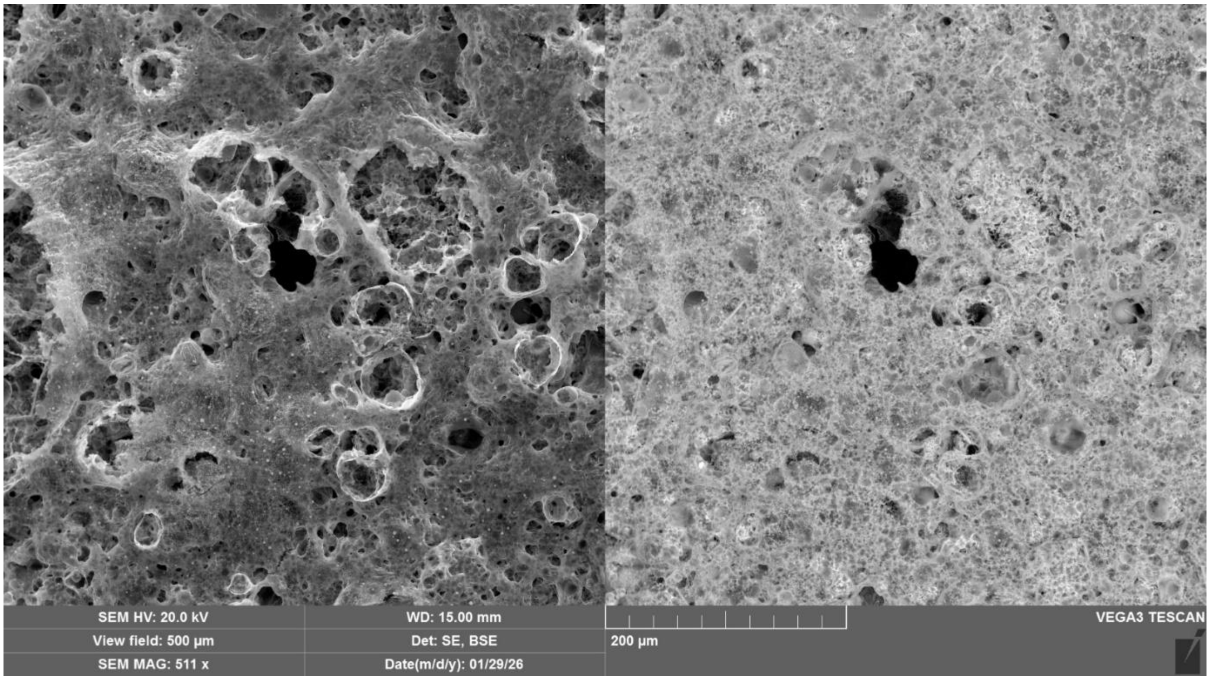

Figure S46. SEM of foamed char layer of formulation I 100/0 sample

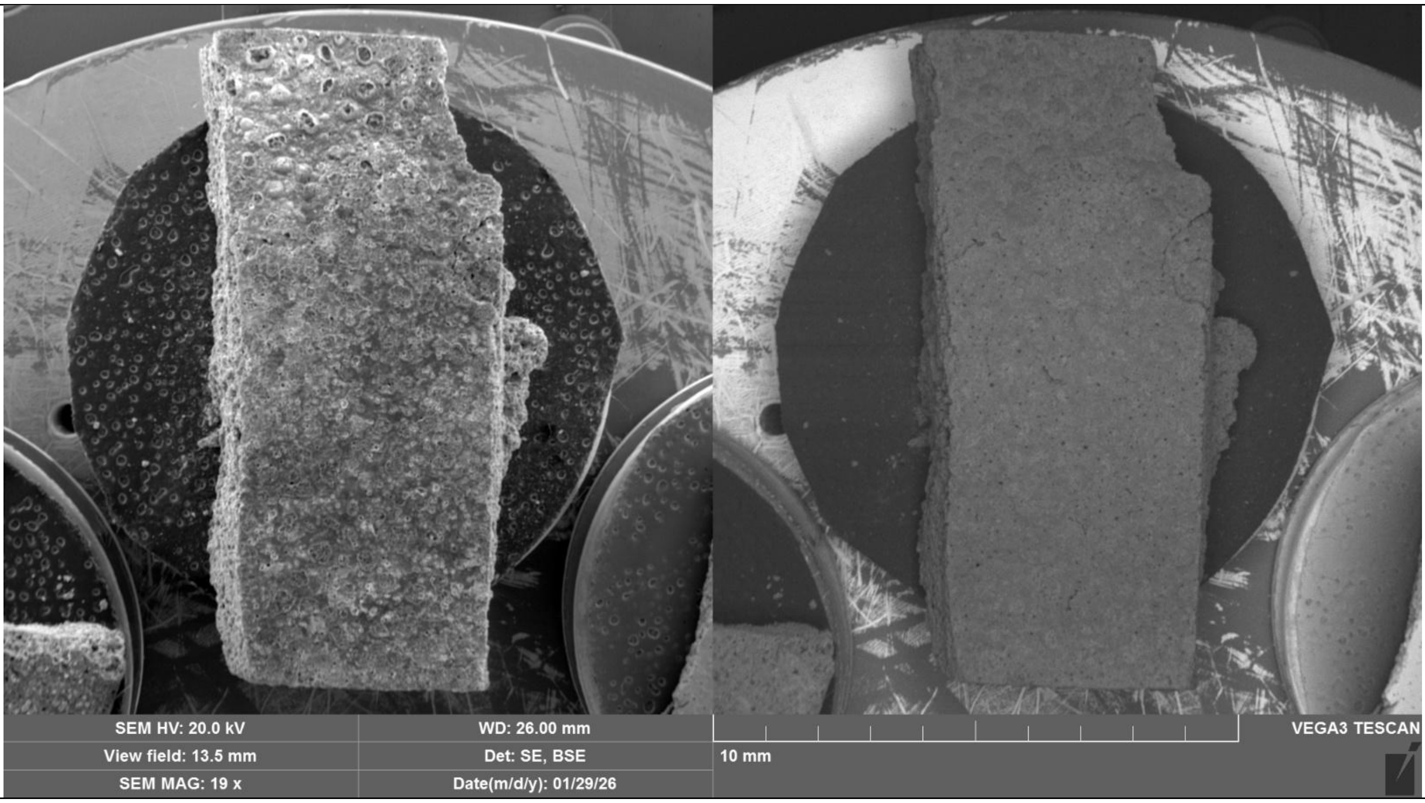

Figure S47. SEM of foamed char layer of formulation I 100/0 sample

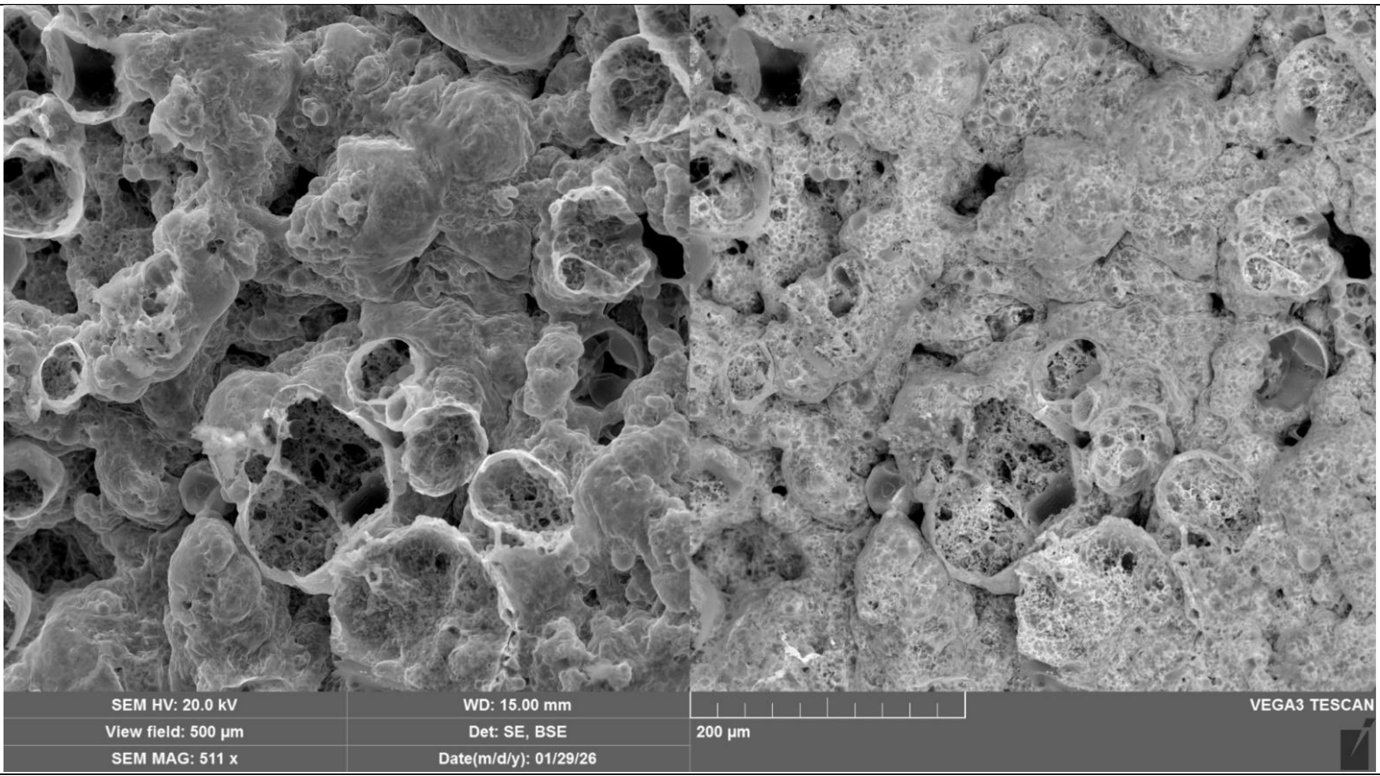

Figure S48. SEM of foamed char layer of formulation I 75/25 sample

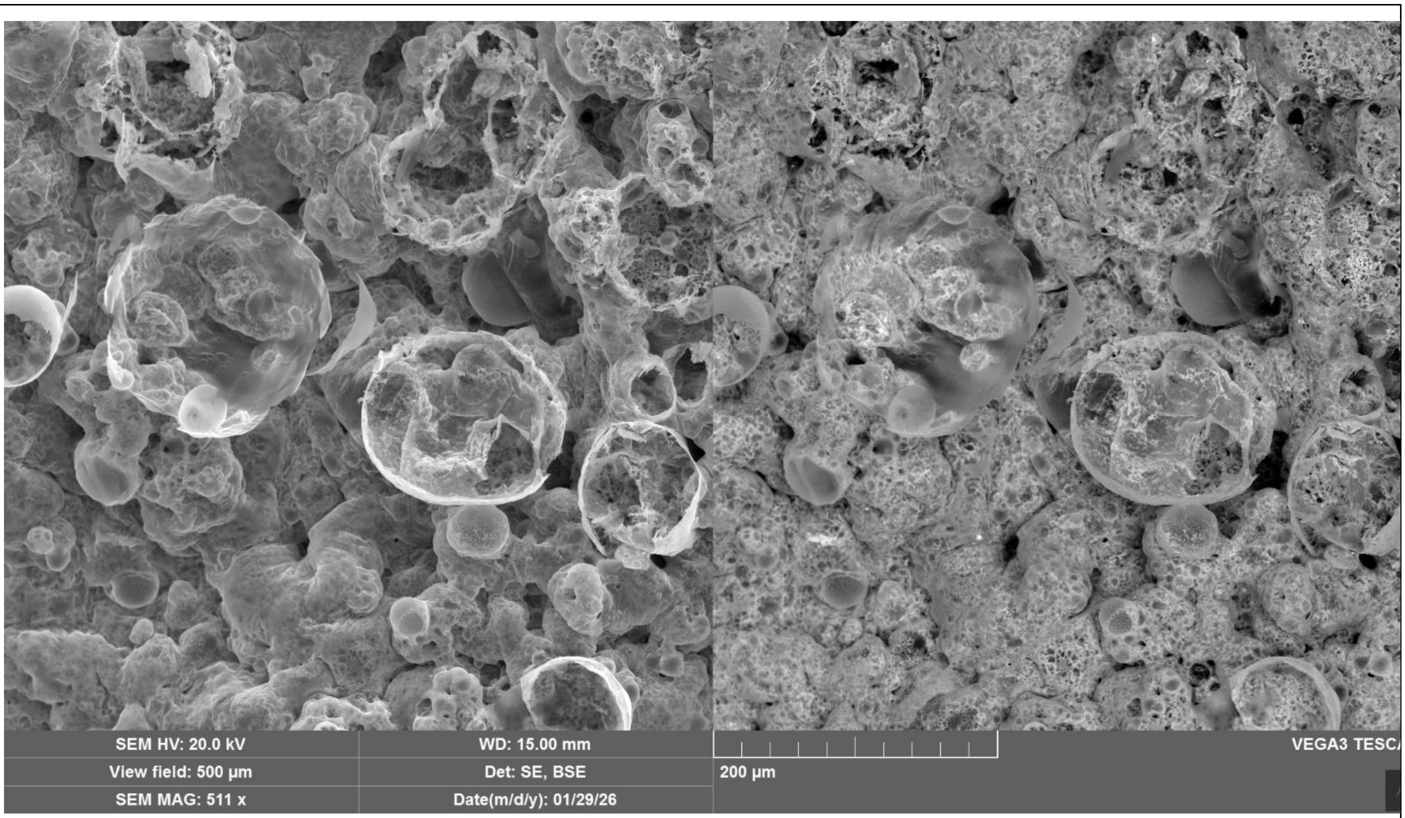

Figure S49. SEM of foamed char layer of formulation **I** 75/25 sample

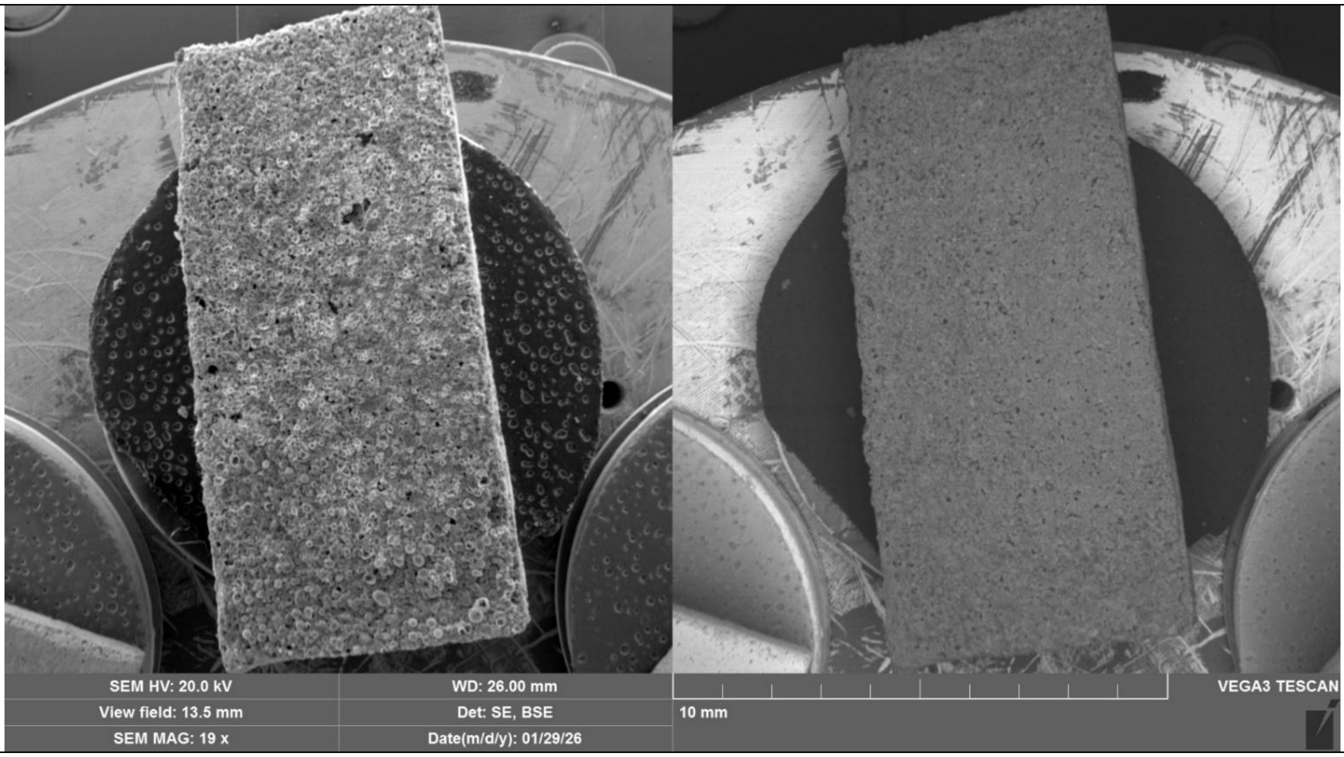

Figure S50. SEM of foamed char layer of formulation **I** 75/25 sample

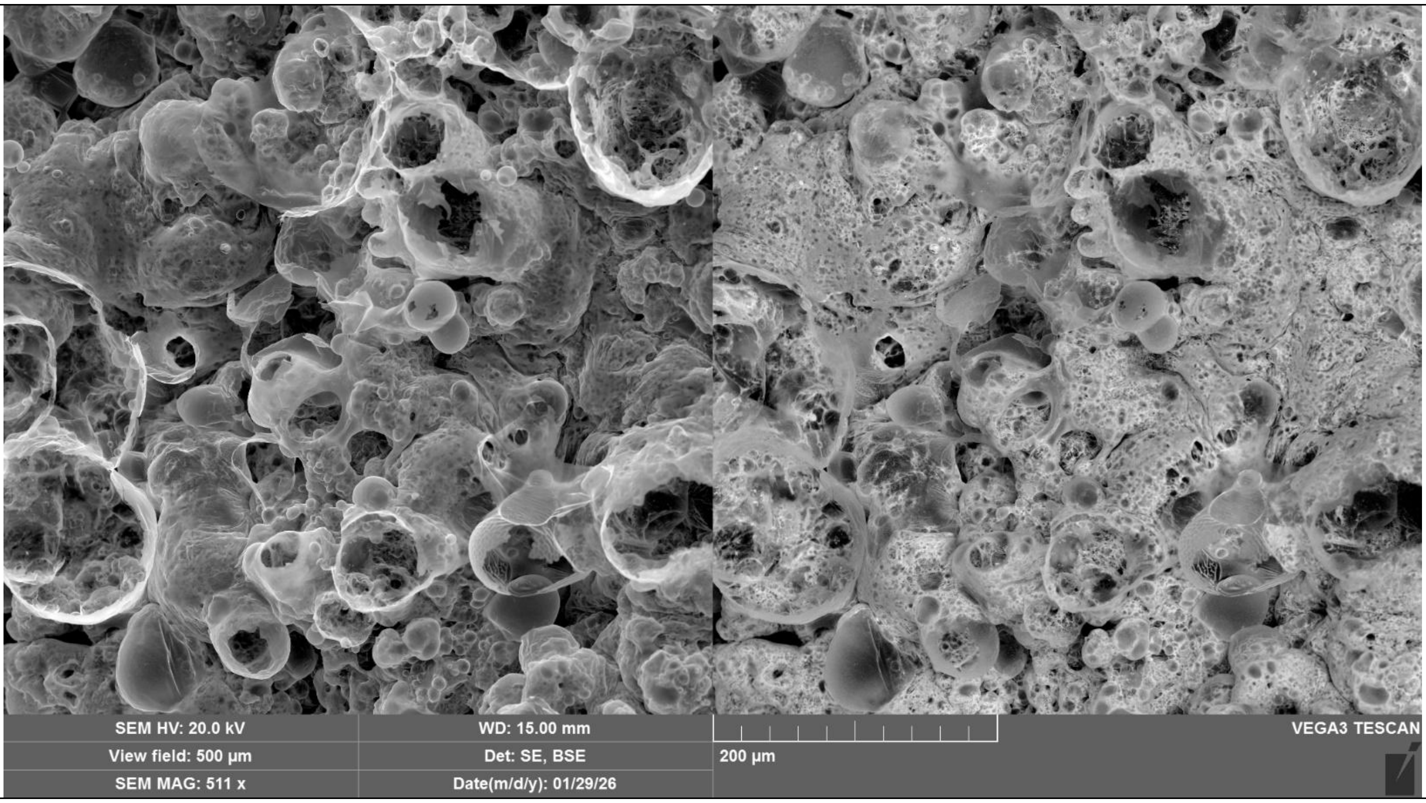

Figure S51. SEM of foamed char layer of formulation **I** 50/50 sample

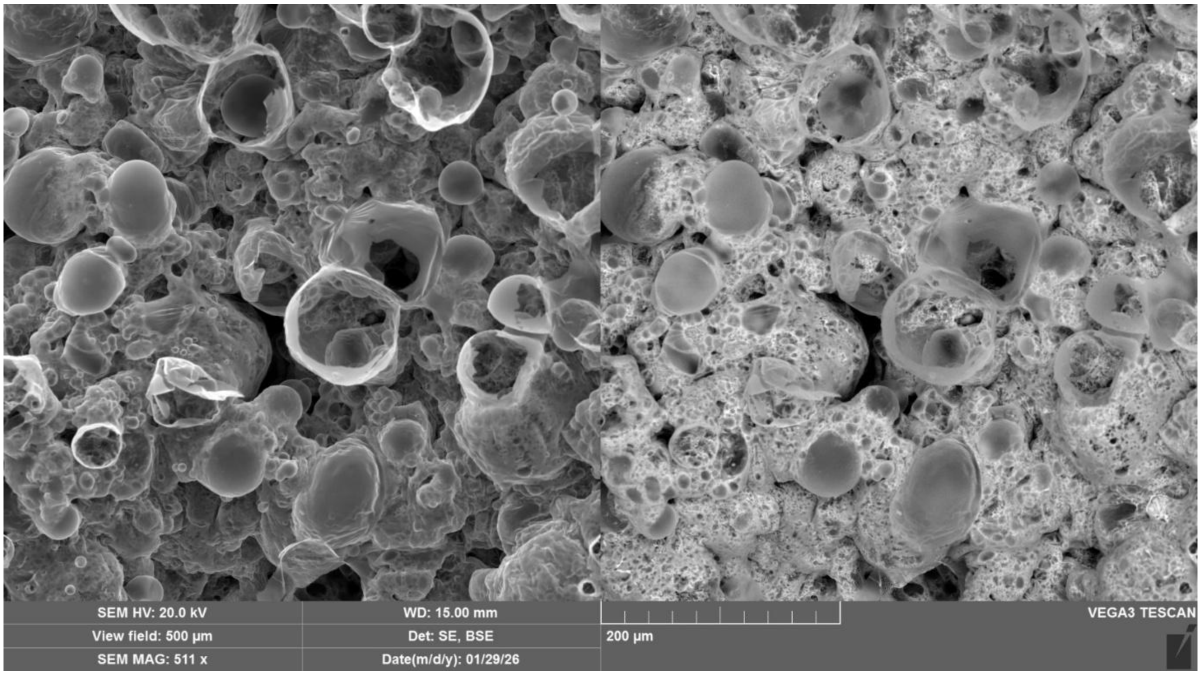

Figure S52. SEM of foamed char layer of formulation I 50/50 sample

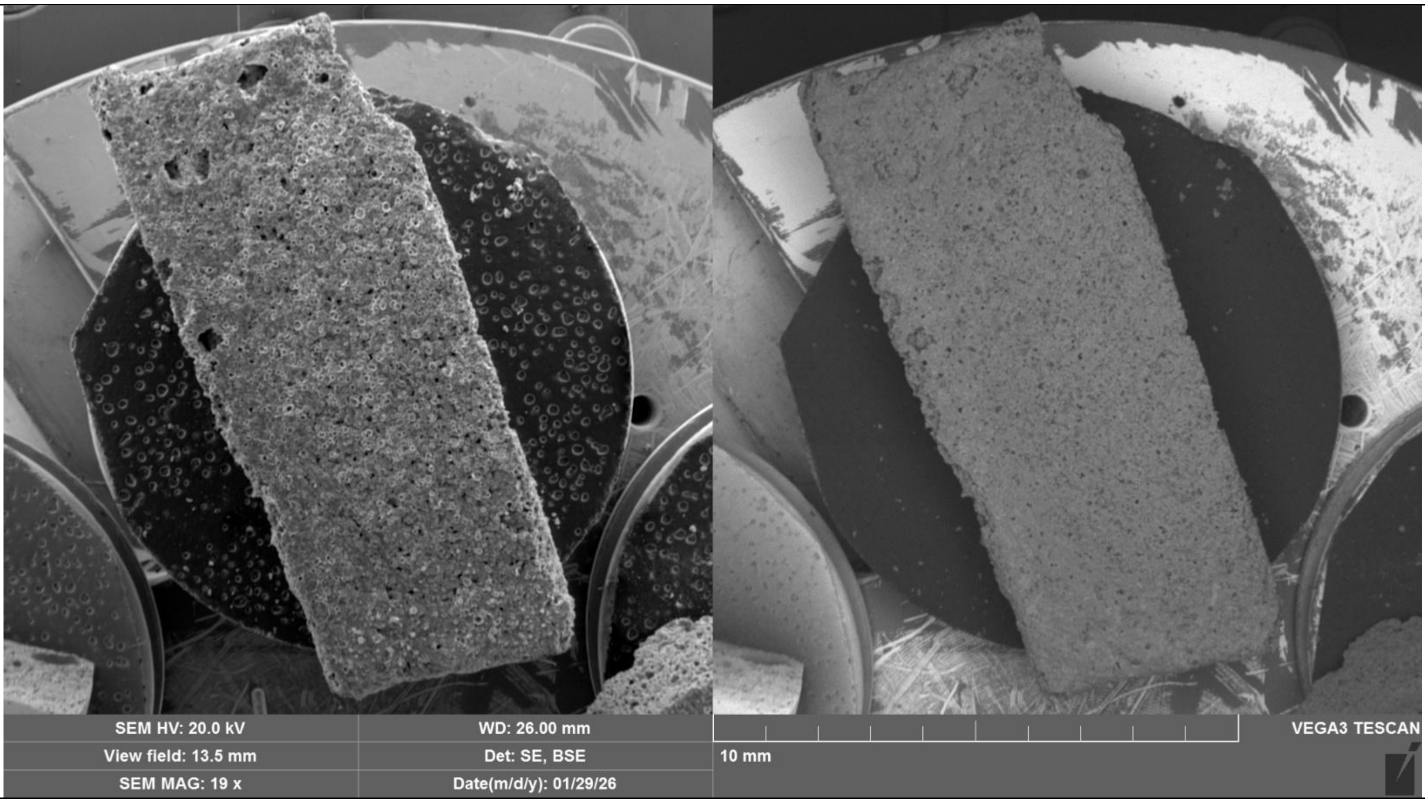

Figure S53. SEM of foamed char layer of formulation **I** 50/50 sample

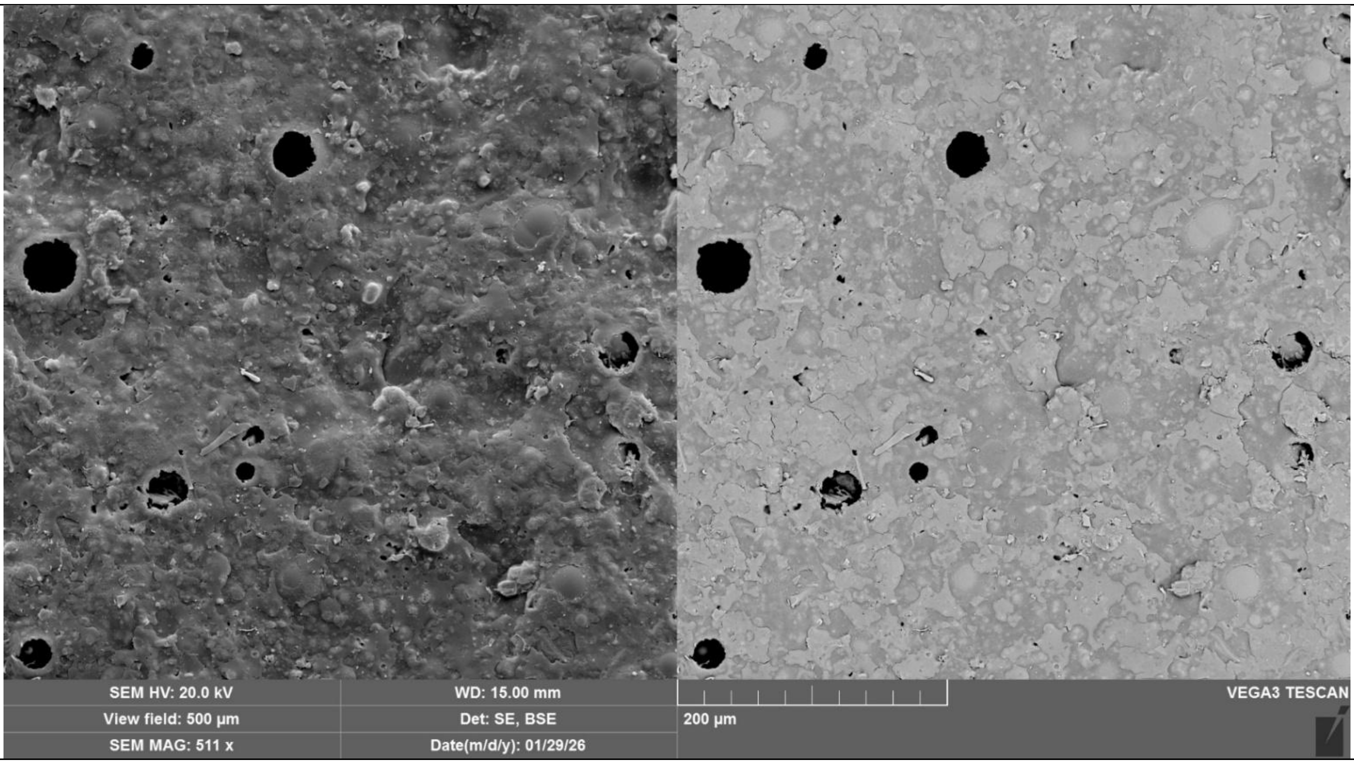

Figure S54. SEM of IFP coating of formulation **I** 100/0 sample

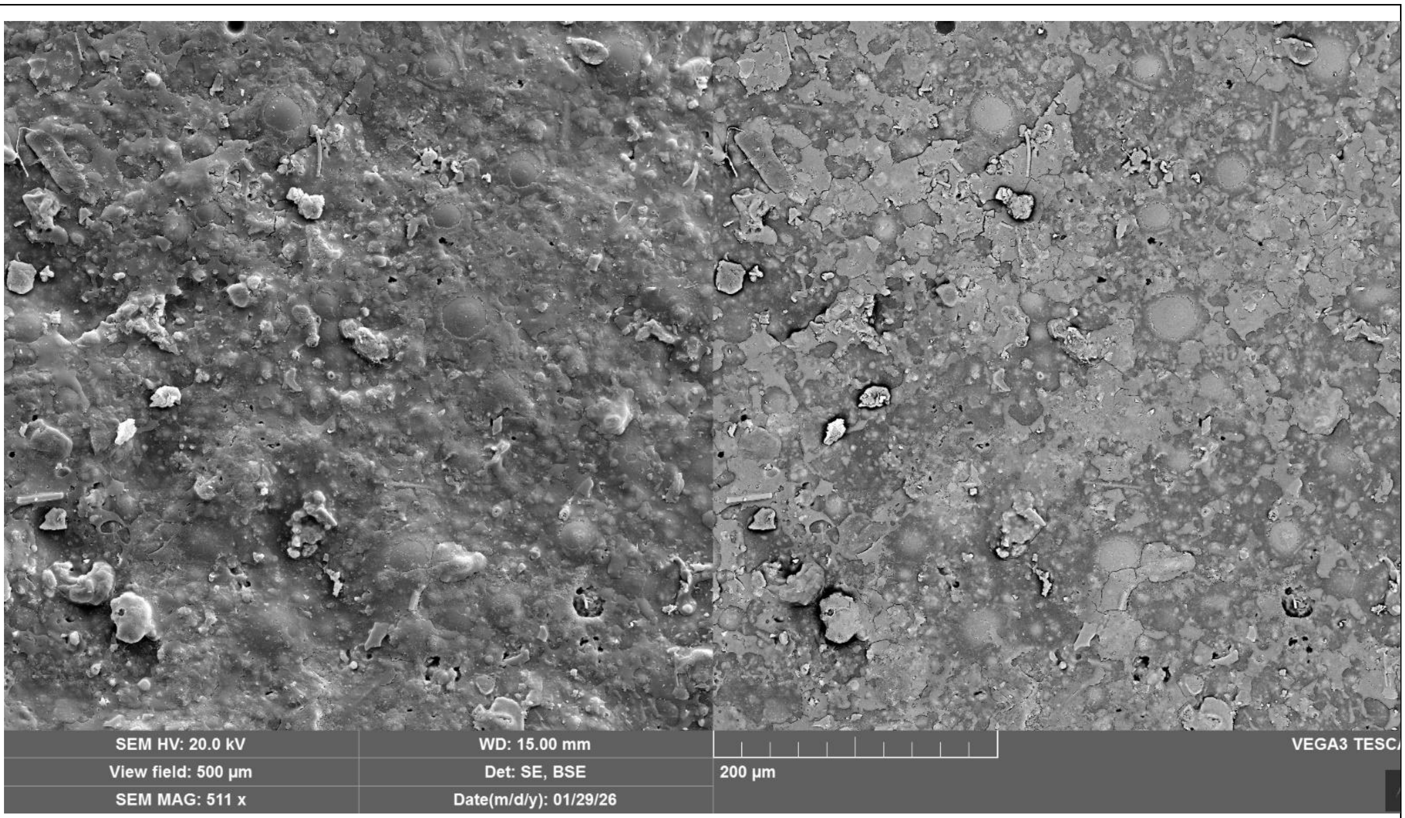

Figure S55. SEM of IFP coating of formulation **I** 100/0 sample

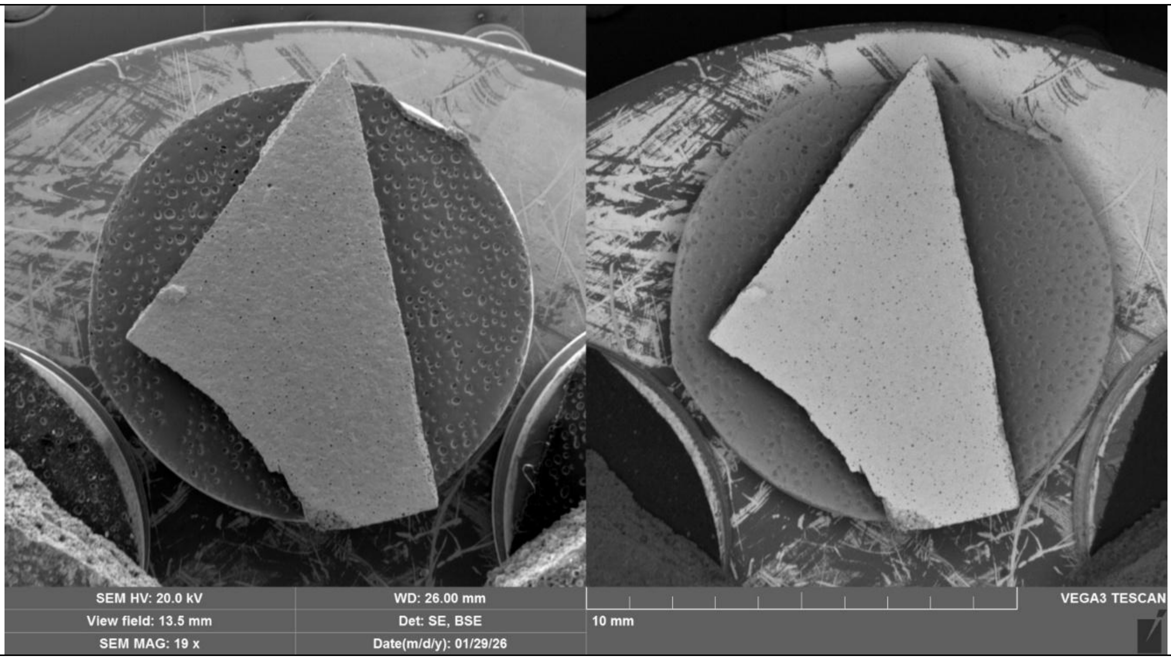

Figure S56. SEM of IFP coating of formulation **I** 100/0 sample

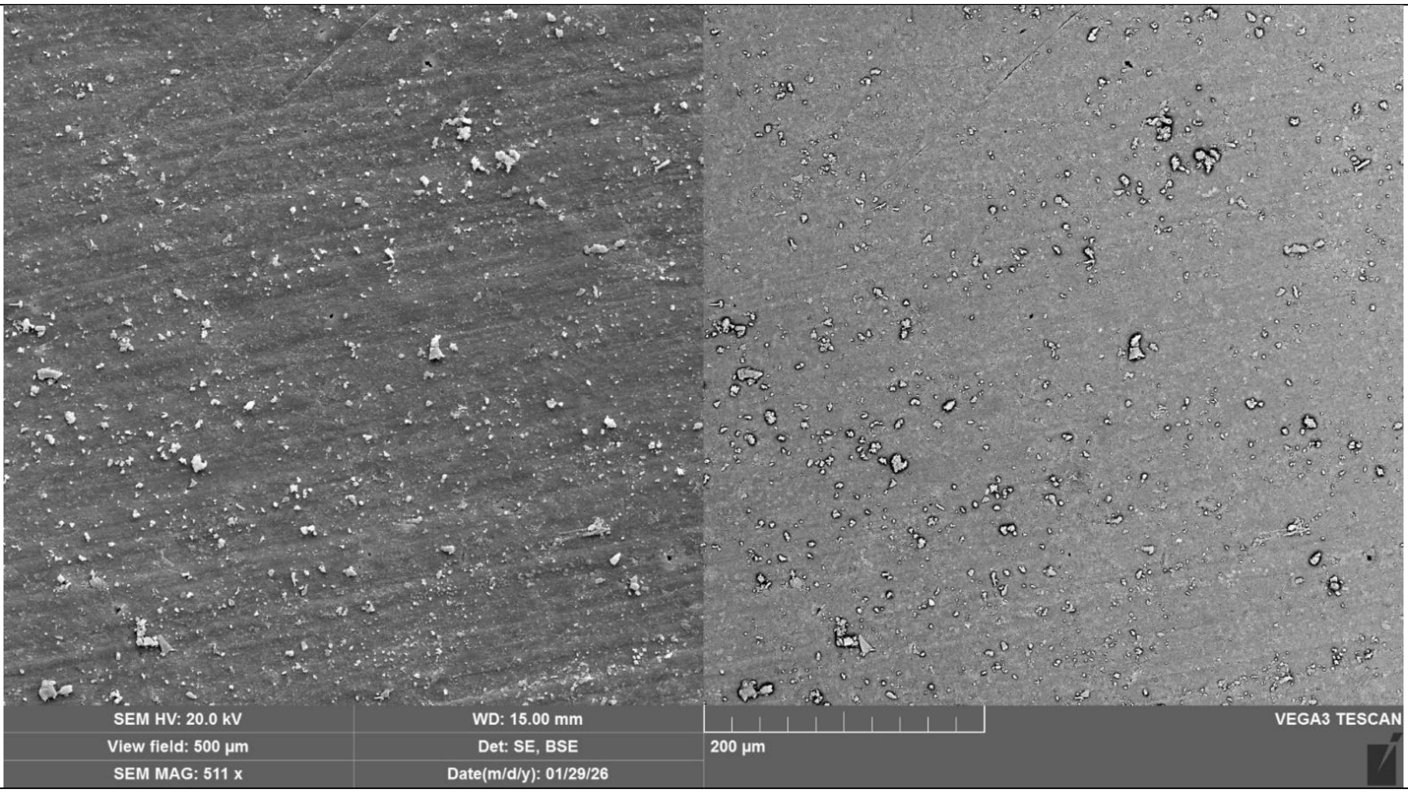

Figure S57. SEM of IFP coating of formulation **I** 75/25 sample

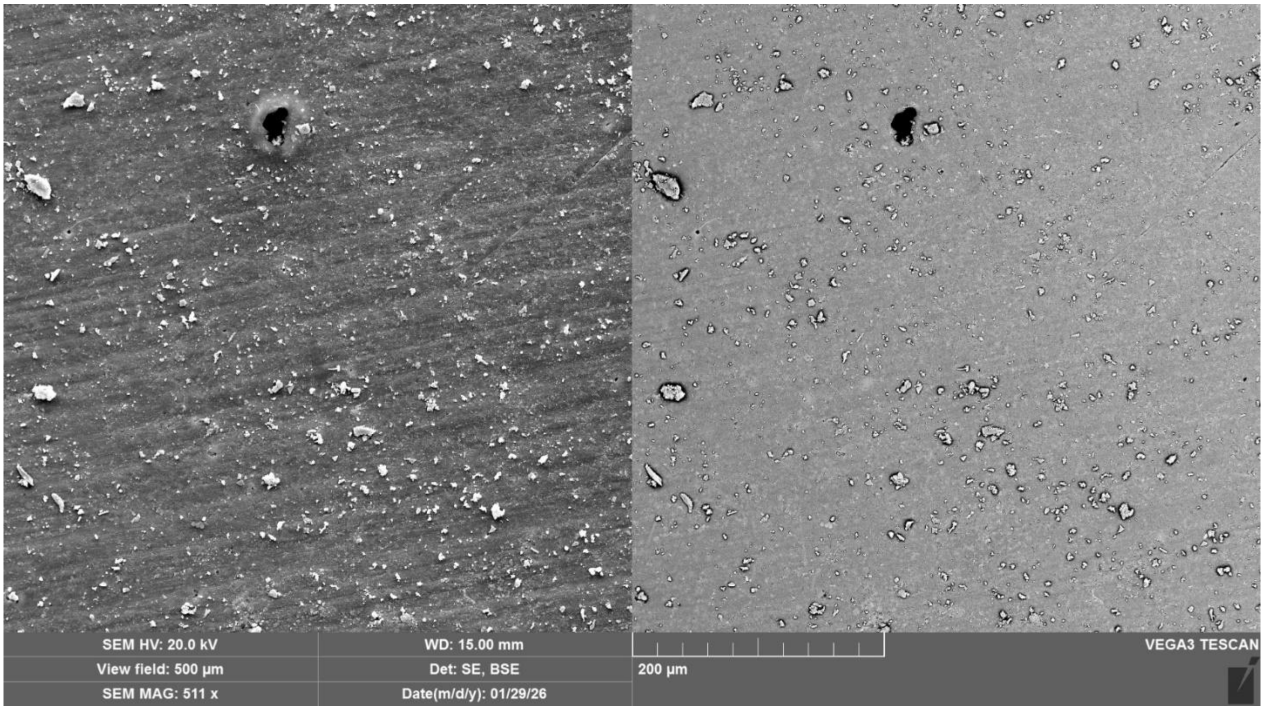

Figure S58. SEM of IFP coating of formulation **I** 75/25 sample

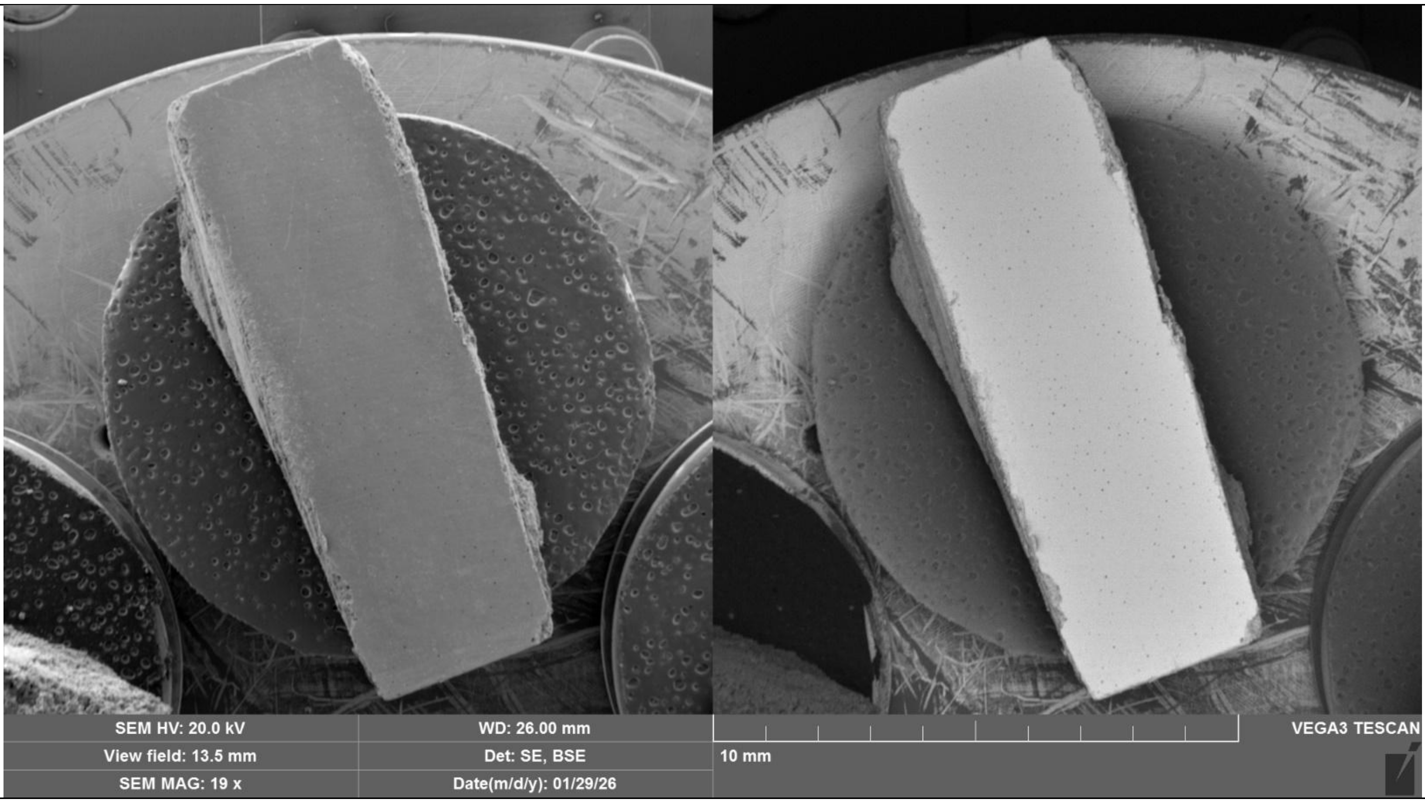

Figure S59. SEM of IFP coating of formulation **I** 75/25 sample

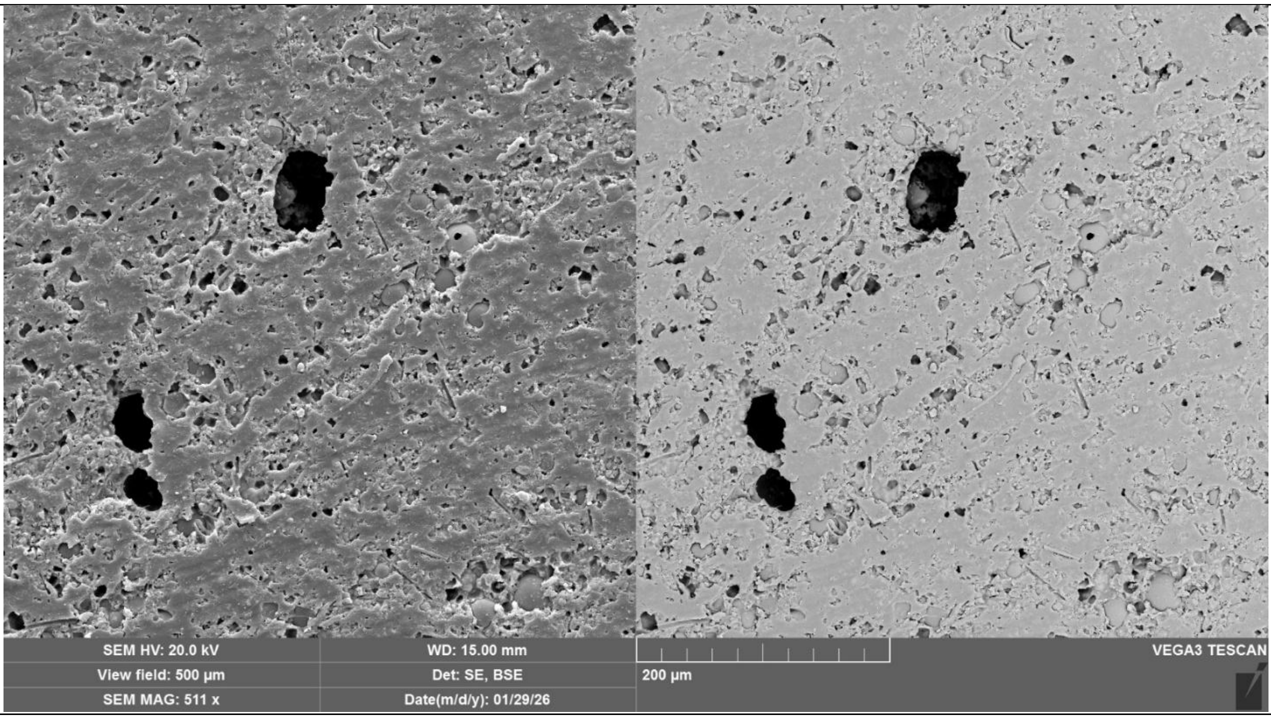

Figure S60. SEM of IFP coating of formulation **I** 50/50 sample

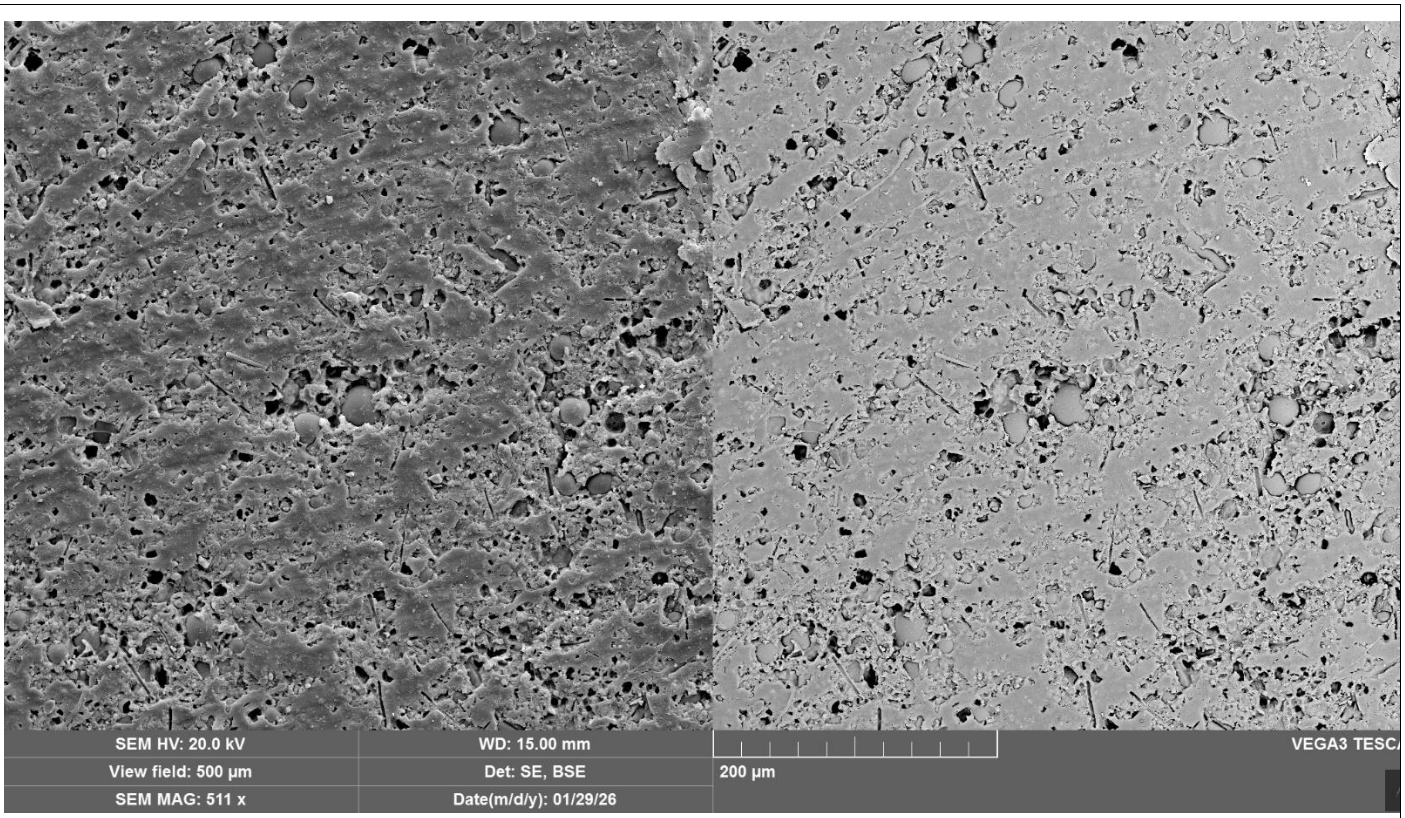

Figure S61. SEM of IFP coating of formulation **I** 50/50 sample

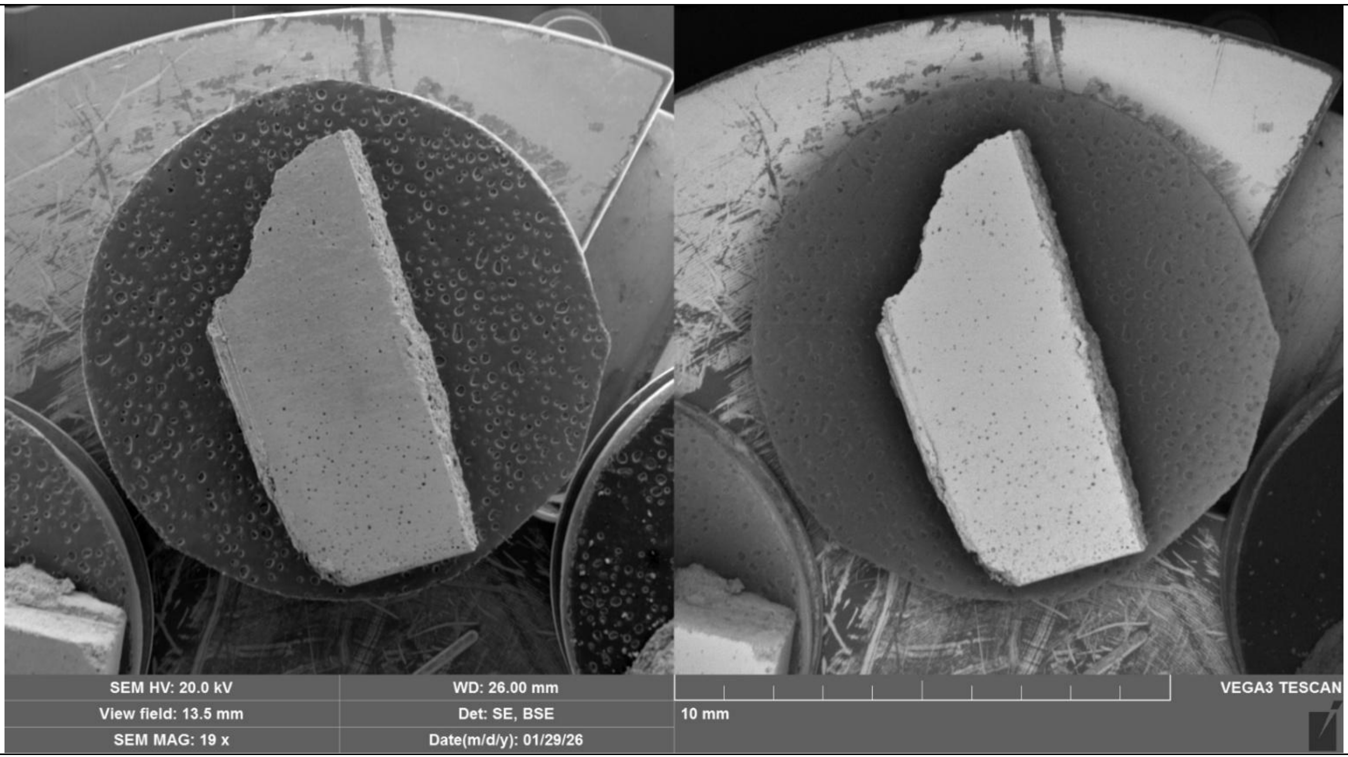

Figure S62. SEM of IFP coating of formulation **I** 50/50 sample

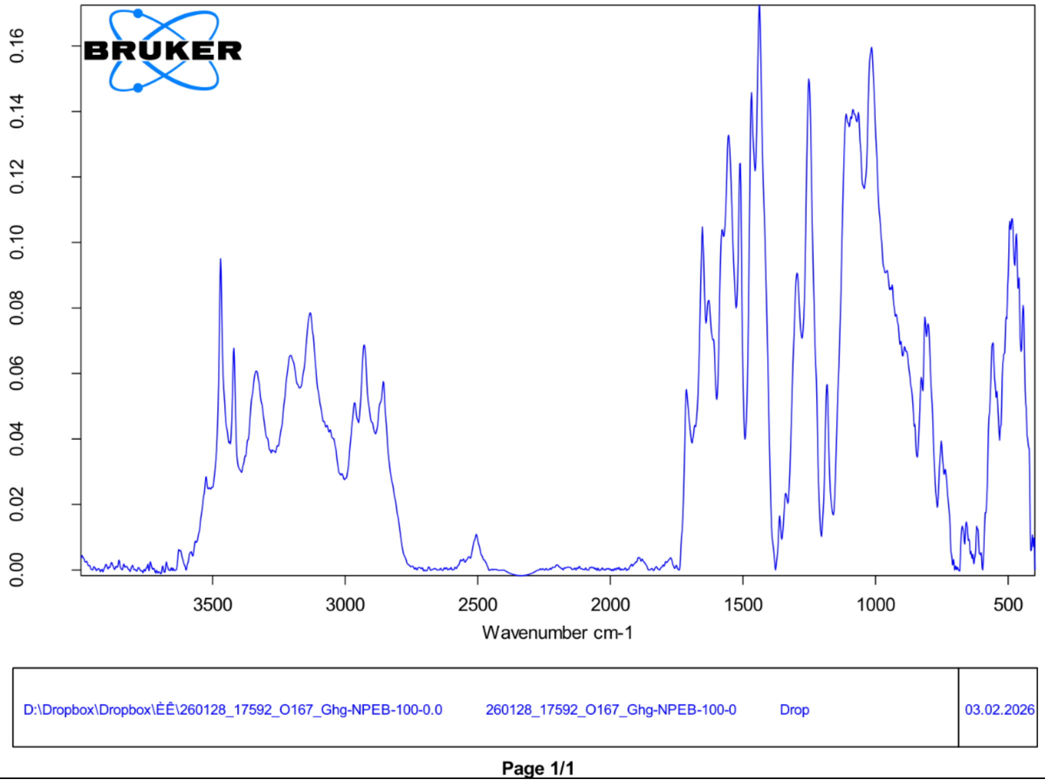

Figure S63. IR spectra for 100/0 IFP coating sample of formulation I

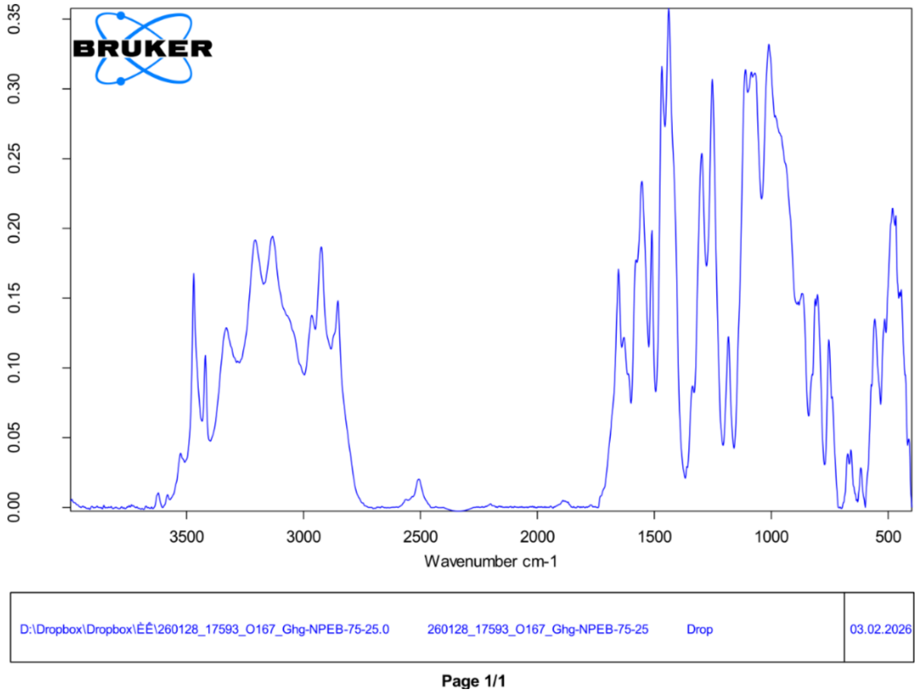

Figure S64. IR spectra for 75/25 IFP coating sample of formulation I

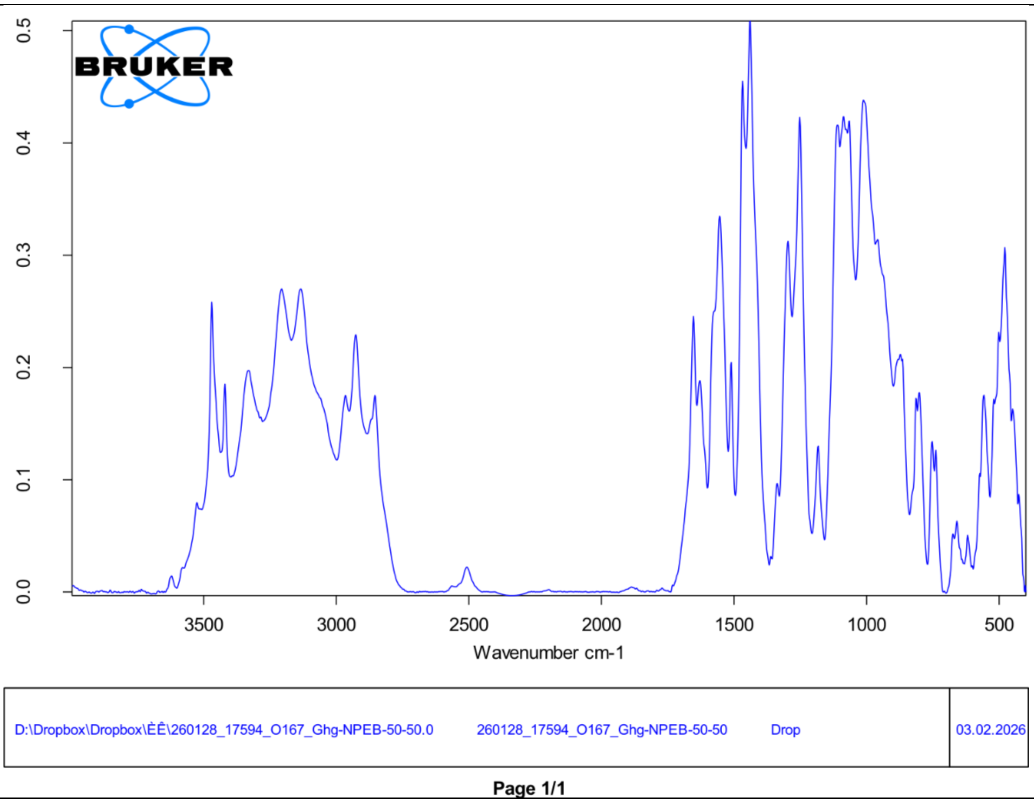

Figure S65. IR spectra for 50/50 IFP coating sample of formulation I

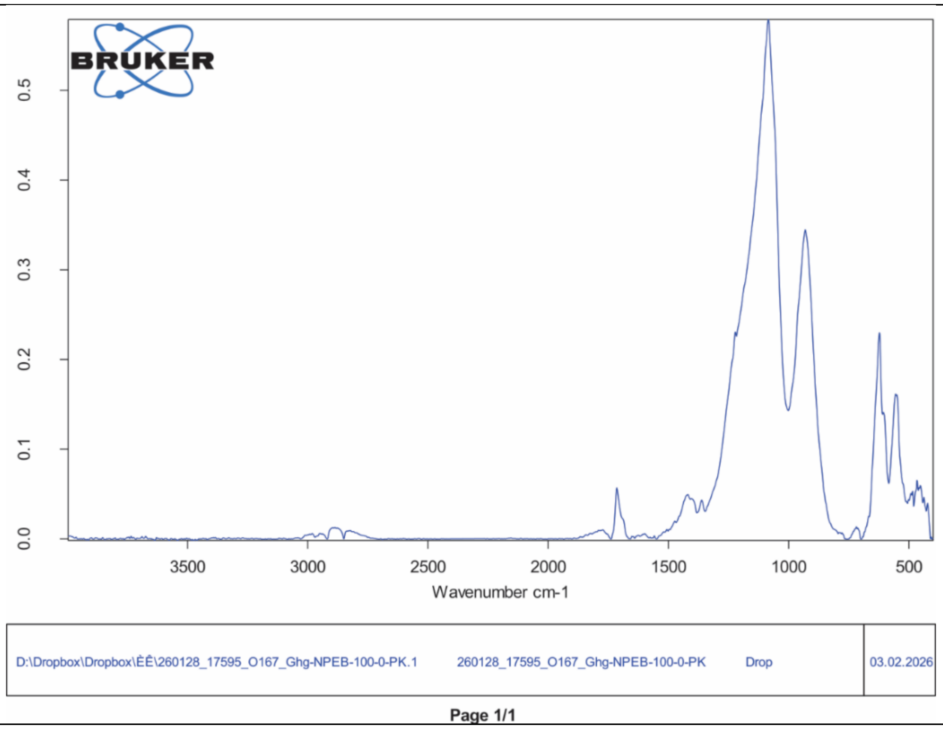

Figure S66. IR spectra of foamed char layer obtained from 100/0 sample of formulation I

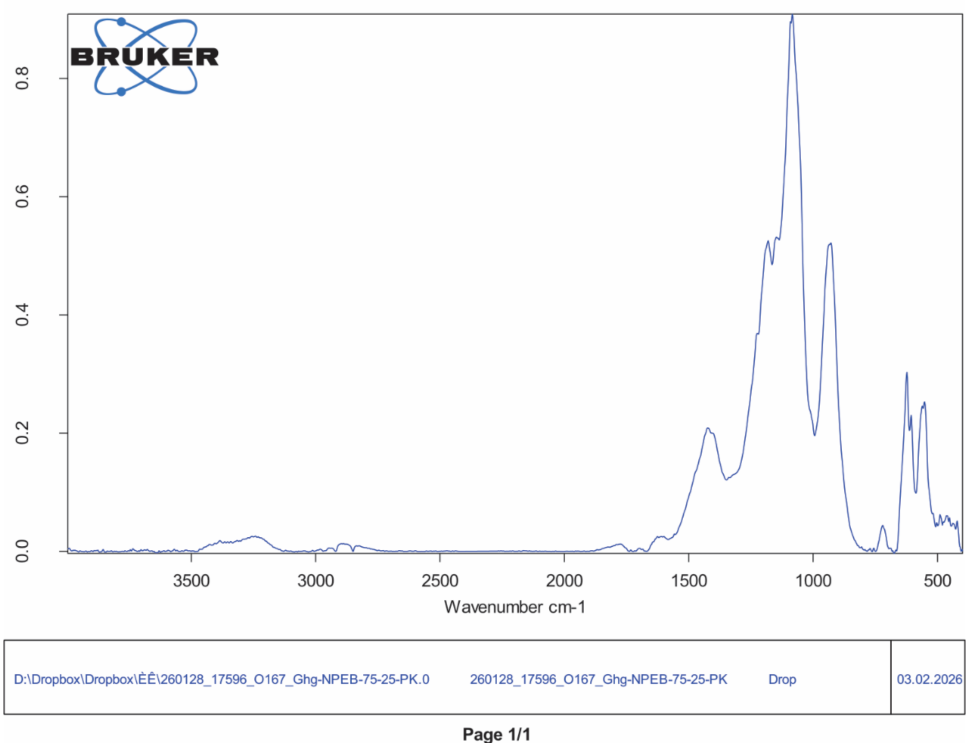

Figure S67. IR spectra of foamed char layer obtained from 75/25 sample of formulation I

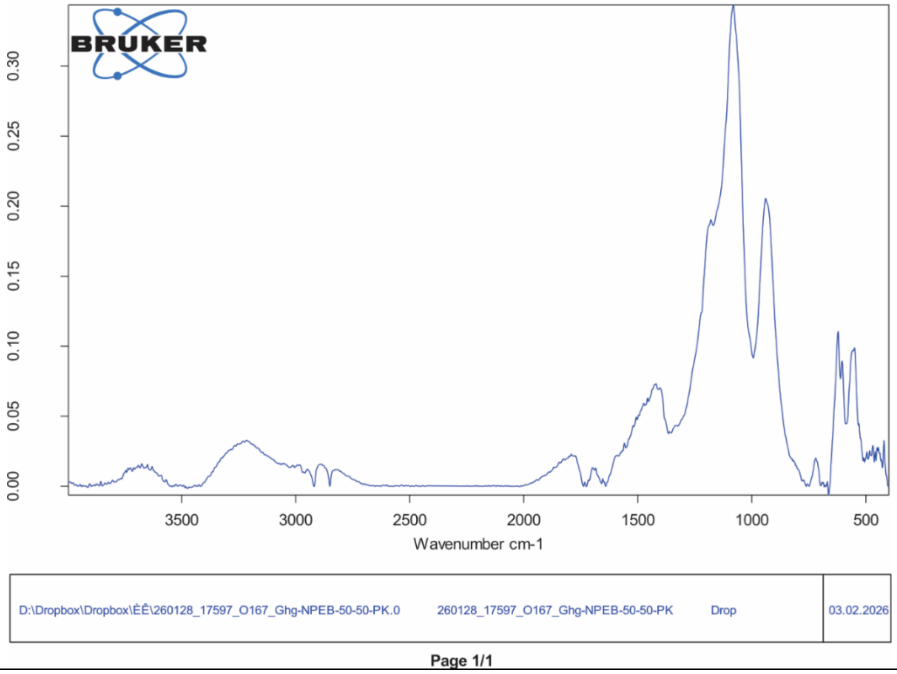

Figure S68. IR spectra of foamed char layer obtained from 50/50 sample of formulation **I**

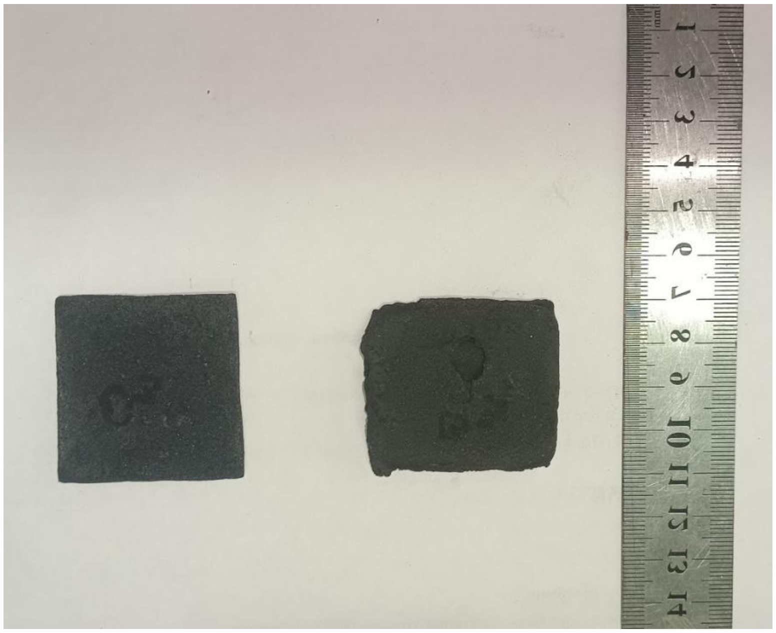

Figure S69. NPEL-128/NPEB-400=100/0 (left) and 0/100 (right) Composition I foam coke samples after the coefficient of thermal expansion of coatings determination

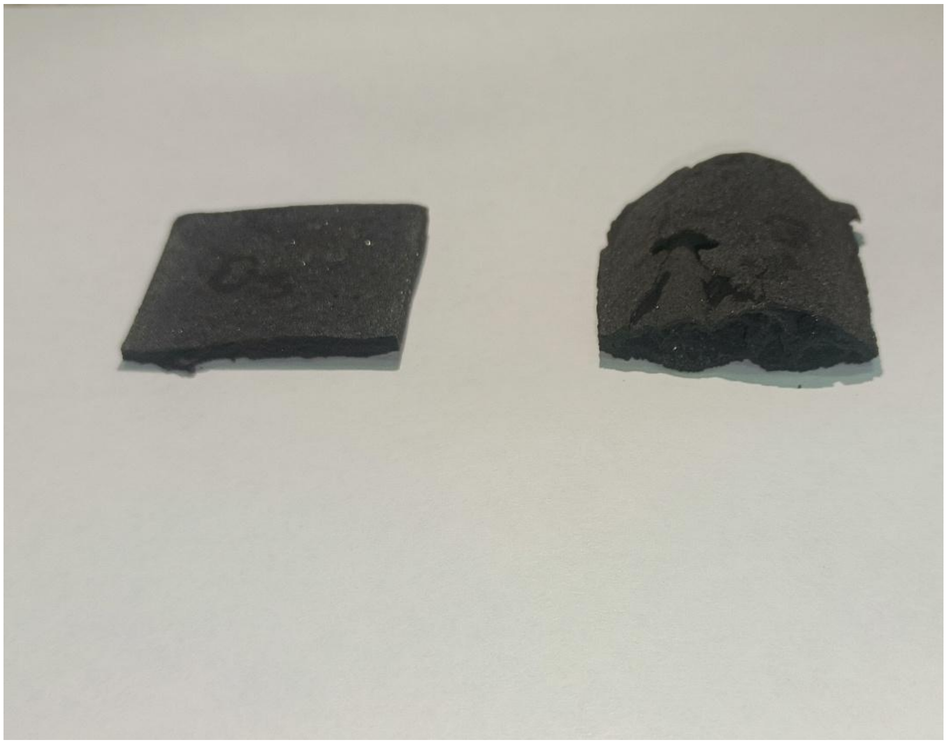

Figure S70. NPEL-128/NPEB-400=100/0 (left) and 0/100 (right) foam coke samples of the first composition a determining the coefficient of thermal expansion of coatings (in the section)
